# Supplementary material for: Relief of excited-state antiaromaticity enables the smallest red emitter
Source: Nat Commun. 2021 Sep 13;12:5409. doi: 10.1038/s41467-021-25677-2 (PMC8438045; doi:10.1038/s41467-021-25677-2)
Supplement: Supplementary file 1 — Supplementary Information [file 41467_2021_25677_MOESM1_ESM.pdf]

Supplementary Information

Relief of Excited-State Antiaromaticity Enables the Smallest Red Emitter

Heechan Kim,[1,†] Woojin Park,[2,†] Younghun Kim,[1] Michael Filatov,[2,*] Cheol Ho Choi,[2,*] and Dongwhan Lee[1,*]

[1]*Department of Chemistry, Seoul National University, 1 Gwanak-ro, Gwanak-gu, Seoul 08826, Korea*
[2]*Department of Chemistry, Kyungpook National University, 80 Daehak-ro, Daegu 41566, Korea*

Experimental Section

General Considerations. All reagents were purchased from commercial suppliers and used as received unless otherwise noted. Acetonitrile, THF, and Et_2O used for spectroscopic studies were saturated with argon and purified by passage through activated Al_2O_3 columns under argon (Innovative Technology SPS PureSolv MD4). Spectroscopic grade cyclohexane, toluene, CH_2Cl_2, $CHCl_3$, EtOAc, DMSO, and EtOH were used for solvent-dependent photophysical measurements. All air-sensitive manipulations were carried out under argon atmosphere by standard Schlenk-line techniques. The compounds 3,6-bis((trimethylsilyl)ethynyl)benzene-1,2-diamine[1], 1,3-bis(acetamido)-4,6-bis(trimethylsilylethynyl)-benzene[2], and 2,5-bis((trimethylsilyl)ethynyl)benzene-1,4-diamine[3] were prepared according to the literature procedures or their slight modifications.

1,1′-(2,3-Diamino-1,4-phenylene)bis(ethan-1-one) (*o*-DAPA). A 100 mL 2-neck round-bottom flask was charged with 3,6-bis((trimethylsilyl)ethynyl)benzene-1,2-diamine (0.580 g, 2.16 mmol) and *p*-toluenesulfonic acid monohydrate (4.2 g, 22 mmol) under an argon atmosphere. A portion of argon-saturated anhydrous ethanol (16 mL) was added, followed by H_2O (4 mL). The reaction mixture was heated at reflux for 15 h. After cooling to r.t., the reaction mixture was neutralized by addition of sat'd aq. $NaHCO_3$, and concentrated under reduced pressure. The residual material was extracted into CH_2Cl_2 (50 mL × 3), and the combined organic layer was dried over anhyd $MgSO_4$, filtered, and concentrated under reduced pressure. Flash column chromatography on SiO_2 (hexane:EtOAc = 9:1 to 1:1, v/v) furnished ***o*-DAPA** as a orange solid (0.185 g, 0.962 mmol, yield = 45%). ^1H NMR (500 MHz, $CDCl_3$, 298 K): δ 7.17 (s, 2H), 6.16 (br, 4H), 2.62 (s, 6H). ^{13}C NMR (125 MHz, $CDCl_3$, 298 K) δ 201.55, 140.72, 120.05, 118.53, 28.63. FT-IR (ATR, cm^{-1}): 3463, 3418, 3314, 2999, 1599, 1505, 1466, 1446, 1355, 1338, 1224. 1019, 995, 965, 914, 879. HRMS (ESI) calcd for $C_{10}H_{13}N_2O_2$ $[M + H]^+$ 193.0972, found 193.0971.

1,1′-(4,6-Diamino-1,3-phenylene)bis(ethan-1-one) (*m*-DAPA). The synthesis of ***m*-DAPA** was reported previously[4], but both the spectroscopic and X-ray crystallogrpahic data provided there are consistent with 4,6-bis(1-iminoethyl)benzene-1,3-diol, rather than ***m*-DAPA**. We thus provide a correct synthetic protocol, along with full characterization data here. A 50 mL 2-neck round-bottom flask was charged with 1,3-bis(acetamido)-4,6-bis(trimethylsilylethynyl)benzene (0.310 g, 0.806 mmol) and *p*-toluenesulfonic acid monohydrate (4.7 g, 25 mmol) under an argon atmosphere. A portion of argon-saturated anhydrous ethanol (8 mL) was added, followed by H_2O (2 mL). The reaction mixture was heated at reflux for 15 h. After cooling to r.t., the reaction mixture was neutralized by addition of sat'd aq. $NaHCO_3$, and concentrated under reduced pressure. The residual material was extracted into CH_2Cl_2 (50 mL × 3), and the combined organic layer was dried over anhyd $MgSO_4$, filtered, and concentrated under reduced pressure. Flash column chromatography on SiO_2 (hexane:EtOAc = 9:1 to 1:2, v/v) furnished ***m*-DAPA** as an off-white solid (0.108 g, 0.562 mmol, yield = 70%). ^1H NMR (500 MHz, $CDCl_3$, 298 K): δ 8.24 (s, 1H), 6.58 (br, 4H), 5.64 (s, 1H), 2.54 (s, 6H). ^{13}C NMR (125 MHz, $CDCl_3$, 298 K): δ 198.03, 154.56, 140.14, 111.05, 98.33, 26.93. FT-IR (ATR, cm^{-1}): 3447, 3334, 3412, 1616, 1571, 1532, 1490, 1450, 1362, 1318, 1259, 1245, 1047, 938, 848. HRMS (ESI) calcd for $C_{10}H_{13}N_2O_2$ $[M + H]^+$ 193.0972, found 193.0972.

1,1′-(2,5-Diamino-1,4-phenylene)bis(ethan-1-one) (*p*-DAPA). An oven-dried 100 mL 2-neck round-bottom flask was charged with 2,5-bis((trimethylsilyl)ethynyl)benzene-1,4-diamine (0.427 g, 1.42 mmol) and *p*-toluenesulfonic acid monohydrate (2.73 g, 14.3 mmol) under an argon atmosphere. A portion of argon-saturated anhydrous ethanol (12 mL) was added, foloowed by H_2O (3 mL). The reaction mixture was heated at reflux for 14 h. After cooling to r.t., the reaction mixture

was neutralized by addition of sat'd aq. $NaHCO_3$, and concentrated under reduced pressure. The residual material was extracted into EtOAc (200 mL) and the organic layer was washed with water (75 mL × 3), dried over anhyd Na_2SO_4, filtered, and concentrated under reduced pressure. Flash column chromatography on SiO_2 (hexane:EtOAc = 9:1 to 2:8, v/v) furnished ***p*-DAPA** as a dark purple solid (0.171 g, 0.891 mmol, yield = 63%). ^1H NMR (500 MHz, $CDCl_3$, 298 K): δ 7.09 (s, 2H), 5.49 (br, 4H), 2.57 (s, 6H). ^{13}C NMR (125 MHz, $CDCl_3$, 298 K): δ 200.66, 139.95, 124.21, 119.78, 28.26. FT-IR (ATR, cm^{-1}): 3455, 3337, 3006, 2962, 2920, 2852, 1633, 1575, 1496, 1421, 1358, 1337, 1289, 1203, 1021, 947, 870, 658. HRMS (ESI) calcd for $C_{10}H_{13}N_2O_2$ $[M + H]^+$ 193.0972, found 193.0971.

3,3′-(2,5-Diacetyl-1,4-phenylene)bis(6,6-dimethyl-3-azabicyclo[3.1.0]hexane-2,4-dione) (4). A mixture of ***p*-DAPA** (148 mg, 0.767 mmol) and 6,6-dimethyl-3-oxabicyclo[3.1.0]hexane-2,4-dione (237 mg, 1.70 mmol) in AcOH (0.7 mL) was heated at reflux for overnight. After the reaction was complete, the mixture was cooled to r.t., poured into water (20 mL), and extracted into CH_2Cl_2 (40 mL × 3). The combined extracts were dried over anhyd $MgSO_4$, filtered, and concentrated under reduced pressure. The residual material was purified by flash column chromatography on SiO_2 (hexane:EtOAc = 1:0 to 1:1, v/v). The isolated solid material was dissolved in CH_2Cl_2 (5 mL), and hexane (30 mL) was added to induce precipitation. The precipitate was isolated by filtration to furnish **4** as an off-white solid (122 mg, 0.280 mmol, yield = 36%). ^1H NMR (500 MHz, $CDCl_3$, 298 K): δ 7.53 (br, 2H), 2.55 (s, 6H), 2.52 (s, 4H), 1.48 (s, 6H), 1.32 (s, 6H). ^{13}C NMR (125 MHz, $CDCl_3$, 298 K): δ 197.50, 172.20, 139.09, 128.47, 127.96, 35.99, 34.28, 28.38, 26.53, 16.23. FT-IR (ATR, cm^{-1}): 3077, 2934, 1771, 1710, 1707, 1503, 1409, 1359, 1337, 1294, 1249, 1207, 1181, 1170, 1065, 994, 959, 914, 894, 867, 801. HRMS (ESI) calcd for $C_{24}H_{24}N_2O_6Na$ $[M + Na]^+$ 459.1527, found 459.1526.

***N,N′*-(2,5-Diacetyl-1,4-phenylene)diacetamide (5).** An oven-dried 100 mL 2-neck round-bottom flask was charged with ***p*-DAPA** (70.0 mg, 0.364 mmol), dry CH_2Cl_2 (7 mL), and dry Et_3N (0.13 mL, 0.91 mmol) under an argon atmosphere. The solution was stirred at 0 °C, and acetyl chloride (80 μL, 1.1 mmol) was added dropwise over a period of 5 min. The reaction mixture was stirred at r.t. for 28 h, diluted with EtOAc (100 mL), and washed with water (25 mL × 3). The combined organic layer was dried over anhyd Na_2SO_4, filtered, and concentrated under reduced pressure. Flash column chromatography on SiO_2 (hexane:EtOAc = 9:1 to 2:3, v/v) furnished **5** as a yellow solid (75.5 mg, 0.273 mmol, yield = 75%). ^1H NMR (500 MHz, $CDCl_3$, 298 K): δ 11.36 (s, 2H), 9.45 (s, 2H), 2.72 (s, 6H), 2.24 (s, 6H). ^{13}C NMR (125 MHz, $CDCl_3$, 298 K): δ 203.13, 169.60, 134.94, 125.41, 123.53, 28.95, 25.45. FT-IR (ATR, cm^{-1}): 3239, 3132, 2921, 2852, 1686, 1659, 1551, 1422, 1396, 1361, 1313, 1243, 1226, 1191, 1014, 953, 906, 750, 654. HRMS (ESI) calcd for $C_{14}H_{16}N_2O_4Na$ $[M + Na]^+$ 299.1002, found 299.1002.

Di-*tert*-butyl (2,5-diacetyl-1,4-phenylene)dicarbamate (6). A 100 mL round-bottom flask was charged with ***p*-DAPA** (83.1 mg, 0.432 mmol), Boc_2O (0.7 mL, 3.0 mmol) and EtOH (10 mL). After heating at reflux for 1 d, an additional amount of Boc_2O (0.3 mL, 1.3 mmol) was added. The mixture was heated at reflux for 1 d. After cooling to r.t., the mixture was diluted with CH_2Cl_2 (50 mL) and H_2O (50 mL). The aqueous phase was extracted into CH_2Cl_2 (50 mL × 3), and the combined extracts were dried over anhyd $MgSO_4$, filtered, and concentrated under reduced pressure. Flash column chromatography on SiO_2 (hexane:EtOAc = 10:0 to 8:2, v/v) furnished **6** as a yellow solid (72.0 mg, 0.183 mmol, yield = 42%). ^1H NMR (500 MHz, $CDCl_3$, 298 K): δ 10.49 (s, 2H), 9.08 (s, 2H), 2.72 (s, 6H), 1.53 (s, 18H). ^{13}C NMR (125 MHz, $CDCl_3$, 298 K): δ 202.56, 153.26, 134.55, 125.43, 122.12, 80.63, 28.95, 28.31. FT-IR (ATR, cm^{-1}): 3296, 2980, 2927, 1727, 1713, 1664,

1653, 1543, 1408, 1392, 1367, 1321, 1241, 1227, 1151, 1054, 1028, 952, 897, 843. HRMS (ESI) calcd for $C_{20}H_{28}N_2O_6Na$ [M + Na]$^+$ 415.1840, found 415.1840.

1,1′-(2,5-Diacetyl-1,4-phenylene)bis(3,3-dimethylurea) (7). An oven-dried 100 mL Schlenk flask was charged with ***p*-DAPA** (174 mg, 0.904 mmol), Et_3N (0.380 mL, 2.73 mmol), and anhydrous CH_2Cl_2 (10 mL). With stirring at r.t., dimethylcarbamoyl chloride (0.640 mL, 6.95 mmol) was added dropwise over a period of 10 min. The reaction mixture was stirred at 40 °C for 2 d. After the reaction was complete, the mixture was poured into water (30 mL), and extracted into CH_2Cl_2 (30 mL × 3). The combined extracts were dried over anhyd $MgSO_4$, filtered, and concentrated under reduced pressure. The residual material was purified by flash column chromatography on SiO_2 (hexane:EtOAc = 2:1 to 0:1, v/v) to furnish **7** as an orange solid (115 mg, 0.345 mmol, yield = 38%). ^{1}H NMR (500 MHz, $CDCl_3$, 298 K): δ 11.01 (s, 2H), 9.38 (s, 2H), 3.11 (s, 12H), 2.74 (s, 6H). ^{13}C NMR (125 MHz, CD_2Cl_2, 298 K): δ 203.79, 156.06, 135.53, 125.00, 122.71, 36.33, 28.98. FT-IR (ATR, cm^{-1}): 3275, 3209, 3136, 3000, 2928, 1656, 1643, 1561, 1484, 1403, 1352, 1320, 1223, 1195, 1178, 955, 902, 854. HRMS (ESI) calcd for $C_{16}H_{23}N_4O_4$ [M + H]$^+$ 335.1714, found 335.1714.

***N*-(2,5-Diacetyl-4-aminophenyl)acetamide (8).** An oven-dried 50 mL 2-neck round-bottom flask was charged with ***p*-DAPA** (58.1 mg, 0.302 mmol), dry CH_2Cl_2 (6 mL), and Et_3N (50 μL, 0.40 mmol) under an argon atmosphere. The solution was cooled to 0 °C, and acetic anhydride (19 μL, 0.20 mmol) was added dropwise over a period of 5 min with stirring. The reaction mixture was stirred at r.t. for 4 h, diluted with EtOAc (100 mL), and washed with water (25 mL × 3). The organic layer was dried over anhyd Na_2SO_4, filtered, and concentrated under reduced pressure. Flash column chromatography on SiO_2 pre-treated with 2% Et_3N in hexane (hexane:EtOAc:MeOH = 9:1:0 to 15:4:1, v/v/v) furnished **8** as an orange solid (33.2 mg, 0.0725 mmol, yield = 47%). ^{1}H NMR (500 MHz, $CDCl_3$, 298 K): δ 10.84 (s, 1H), 9.18 (s, 1H), 7.16 (s, 1H), 6.07 (br, 2H), 2.64 (s, 3H), 2.64 (s, 3H), 2.20 (s, 3H). ^{13}C NMR (125 MHz, CD_2Cl_2, 298 K): δ 202.39, 201.26, 168.93, 144.66, 128.96, 127.42, 124.59, 121.58, 119.58, 28.72, 28.32, 25.26. FT-IR (ATR, cm^{-1}): 3430, 3312, 3279, 2920, 2851, 1652, 1627, 1567, 1530, 1416, 1372, 1350, 1310, 1263, 1249, 1223, 1022, 951, 900, 865, 751, 655. HRMS (ESI) calcd for $C_{12}H_{15}N_2O_3$ [M + H]$^+$ 235.1077, found 235.1077.

***tert*-Butyl (2,5-diacetyl-4-aminophenyl)carbamate (9).** A 20 mL vial was charged with ***p*-DAPA** (20.0 mg, 0.104 mmol), Boc_2O (60 μL, 0.26 mmol), and EtOH (3 mL). The mixture was heated at 40 °C overnight. After cooling to r.t., the mixture was diluted with CH_2Cl_2 (15 mL) and H_2O (15 mL). The aqueous phase was extracted into CH_2Cl_2 (15 mL × 3) and the combined extracts were dried over anhyd $MgSO_4$, filtered, and concentrated under reduced pressure. Flash column chromatography on SiO_2 (hexane:EtOAc = 9:1 to 2:1, v/v) furnished **9** as a red solid (20.0 mg, 0.0684 mmol, yield = 66%). ^{1}H NMR (500 MHz, $CDCl_3$, 298 K): δ 9.99 (s, 1H), 8.84 (s, 1H), 7.13 (s, 1H), 5.95 (br, 2H), 2.66 (s, 3H), 2.61 (s, 3H), 1.52 (s, 9H). ^{13}C NMR (125 MHz, CD_2Cl_2, 298 K): δ 201.73, 201.27, 153.43, 143.67, 129.77, 127.32, 123.07, 122.12, 119.54, 80.15, 28.65, 28.37. FT-IR (ATR, cm^{-1}): 3461, 3347, 2979, 2932, 1717, 1654, 1556, 1517, 1418, 1364, 1266, 1224, 1157, 1053, 950, 890, 840. HRMS (ESI) calcd for $C_{15}H_{20}N_2O_4Na$ [M + Na]$^+$ 315.1315, found 315.1315.

3-(2,5-Diacetyl-4-aminophenyl)-1,1-dimethylurea (10). This compound was isolated from the reaction mixture described in the synthesis of compound **7**. Column chromatography on SiO_2 afforded **10** as red solid (55.0 mg, 0.209 mmol, 23%). ^{1}H NMR (500 MHz, $CDCl_3$, 298 K): δ 10.54 (s, 1H), 9.12 (s, 1H), 7.16 (s, 1H), 5.90 (br, 2H), 3.08 (s, 6H), 2.65 (s, 3H), 2.63 (s, 3H). ^{13}C NMR (125 MHz, CD_2Cl_2, 298 K): δ 202.67, 201.65, 156.24, 143.08, 131.61, 126.34, 123.61, 122.64, 119.39, 36.26, 28.70, 28.43. FT-IR (ATR, cm^{-1}): 3451, 3335, 2926, 1646, 1635, 1562, 1524, 1492, 1416,

1362, 1208, 1180, 1069, 1022, 950, 892, 849. HRMS (ESI) calcd for $C_{13}H_{18}N_3O_3$ $[M + H]^+$ 264.1343, found 264.1343.

***N,N′*-(2,5-Diacetyl-1,4-phenylene)bis(2,2,2-trichloroacetamide) (11).** A 100 mL round-bottom flask was charged with ***p*-DAPA** (100 mg, 0.521 mmol), trichloroacetic anhydride (210 μL, 1.15 mmol), Et_3N (220 μL, 1.58 mmol), and CH_2Cl_2 (8 mL). The mixture was heated at 40 °C overnight. After cooling to r.t., the mixture was diluted with CH_2Cl_2 (15 mL) and H_2O (15 mL). The aqueous phase was extracted into CH_2Cl_2 (15 mL × 3), and the combined extracts were dried over anhyd $MgSO_4$, filtered, and concentrated under reduced pressure. Flash column chromatography on SiO_2 (hexane:EtOAc = 100:1 to 10:1, v/v) furnished **11** as a green solid (39.0 mg, 0.0808 mmol, yield = 15%). ^{1}H NMR (500 MHz, $CDCl_3$, 298 K): δ 12.96 (s, 2H), 9.51 (s, 2H), 2.82 (s, 6H). ^{13}C NMR (125 MHz, CD_2Cl_2, 298 K): δ 202.76, 161.06, 134.82, 126.48, 123.68, 77.25, 77.00, 76.74, 28.74. FT-IR (ATR, cm^{-1}): 3412, 3090, 2925, 1738, 1710, 1658, 1552, 1445, 1427, 1402, 1361, 1233, 1217, 1187, 1024, 958, 813. HRMS (ESI) calcd for $C_{14}H_{10}Cl_6N_2O_4Na$ $[M + Na]^+$ 504.8640, found 504.8638.

X-ray Crystallographic Studies on *o*-DAPA. Single crystals of ***o*-DAPA** were prepared by slow diffusion of pentane into a $CHCl_3$ solution of this material. An orange crystal (approximate dimensions 0.219 × 0.149 × 0.115 mm^3) was placed onto a nylon loop with Paratone-N oil, and mounted on an XtaLAB AFC12 (RINC): Kappa dual home/near diffractometer. The data collection was carried out using Cu Kα radiation and the crystal was kept at T = 93 K. A total of 24185 reflections were measured (6.674° $\leq 2\theta \leq$ 159.23°). The structure was solved with SHELXT[5] using direct methods, and refined with SHELXL[6] refinement package of OLEX2[7]. A total of 3939 unique reflections were used in all calculations. The final $R1$ was 0.0492 ($I \geq 2\sigma(I)$) and $wR2$ was 0.1542 (all data). CCDC 2054917 contains the supplementary crystallographic data for this structure.

X-ray Crystallographic Studies on *m*-DAPA. Single crystals of ***m*-DAPA** were prepared by slow diffusion of pentane into a $CHCl_3$ solution of this material. A colorless crystal (approximate dimensions 0.1 × 0.02 × 0.02 mm^3) was placed onto a nylon loop with Paratone-N oil, and mounted on an XtaLAB AFC12 (RINC): Kappa dual home/near diffractometer. The data collection was carried out using Cu Kα radiation and the crystal was kept at T = 93 K. A total of 7158 reflections were measured (6.25° $\leq 2\theta \leq$ 157.34°). The structure was solved with SHELXT[5] using direct methods, and refined with SHELXL[6] refinement package of OLEX2[7]. A total of 2185 unique reflections were used in all calculations. The final $R1$ was 0.0481 ($I \geq 2\sigma(I)$) and $wR2$ was 0.1291(all data). CCDC 2054916 contains the supplementary crystallographic data for this structure.

X-ray Crystallographic Studies on *p*-DAPA. Single crystals of ***p*-DAPA** were prepared by slow diffusion of pentane into a $CHCl_3$ solution of this material. A red crystal (approximate dimensions 0.279 × 0.159 × 0.107 mm^3) was placed onto a nylon loop with Paratone-N oil, and mounted on an XtaLAB AFC12 (RINC): Kappa dual home/near diffractometer. The data collection was carried out using Cu Kα radiation and the crystal was kept at T = 93 K. A total of 5782 reflections were measured (8.63° $\leq 2\theta \leq$ 158.096°). The structure was solved with SHELXT[5] using direct methods, and refined with SHELXL[6] refinement package of OLEX2[7]. A total of 1903 unique reflections were used in all calculations. The final $R1$ was 0.0814 ($I \geq 2\sigma(I)$) and $wR2$ was 0.2716 (all data). CCDC 2054922 contains the supplementary crystallographic data for this structure.

X-ray Crystallographic Studies on 4. Single crystals of **4** were prepared by slow diffusion of pentane into a $CHCl_3$ solution of this material. A colorless crystal (approximate dimensions 0.627 × 0.562 × 0.240 mm^3) was placed onto a nylon loop with Paratone-N oil, and mounted on an XtaLAB AFC12 (RINC): Kappa dual home/near diffractometer. The data collection was carried out using Cu Kα radiation and the crystal was kept at T = 93 K. A total of 20018 reflections were measured (8.936° $\leq 2\theta \leq$ 158.488°). The structure was solved with SHELXT[5] using direct methods, and refined with SHELXL[6] refinement package of OLEX2[7]. A total of 4689 unique reflections were used in all calculations. The final $R1$ was 0.0368 ($I \geq 2\sigma(I)$) and $wR2$ was 0.0963 (all data). CCDC 2054920 contains the supplementary crystallographic data for this structure.

X-ray Crystallographic Studies on 5. Single crystals of **5** were prepared by slow diffusion of pentane into a $CHCl_3$ solution of this material. An orange crystal (approximate dimensions 0.627 × 0.562 × 0.240 mm^3) was coated with Parabar 10312 (Hampton Research Inc.) to mount on the micro-loop under cold nitrogen stream at T = 100 K. The diffraction data was measured using synchrotron radiation (λ = 0.71073 Å) employing a PLSII-2D SMC on a Rayonix MX225HS CCD area detector with high precision one-axis goniostat at Pohang Accelerator Laboratory, Korea. The PAL BL2D-SMDC program[8] was used for data collection, and HKL3000sm (Ver.717)[9] was used for cell refinement, reduction, and absorption correction. A total of 3636 reflections were measured (4.414° $\leq 2\theta \leq$ 67.196°). The structure was solved with SHELXT[5] using direct methods, and refined

with SHELXL[6] refinement package of OLEX2[7]. A total of 2065 unique reflections were used in all calculations. The final $R1$ was 0.0990 ($I \geq 2\sigma(I)$) and $wR2$ was 0.2960 (all data). CCDC 2054914 contains the supplementary crystallographic data for this structure.

X-ray Crystallographic Studies on 6. Single crystals of **6** were prepared by slow diffusion of pentane into a $CHCl_3$ solution of this material. A yellow crystal (approximate dimensions 0.513 × 0.066 × 0.057 mm^3) was placed onto a nylon loop with Paratone-N oil, and mounted on an XtaLAB AFC12 (RINC): Kappa dual home/near diffractometer. The data collection was carried out using Cu Kα radiation and the crystal was kept at T = 93 K. A total of 13880 reflections were measured (7.66° $\leq 2\theta \leq$ 158.63°). The structure was solved with SHELXT[5] using direct methods, and refined with SHELXL[6] refinement package of OLEX2[7]. A total of 4367 unique reflections were used in all calculations. The final $R1$ was 0.0425 ($I \geq 2\sigma(I)$) and $wR2$ was 0.1129 (all data). CCDC 2054915 contains the supplementary crystallographic data for this structure.

X-ray Crystallographic Studies on 7. Single crystals of **7** were prepared by slow diffusion of pentane into a $CHCl_3$ solution of this material. An orange crystal (approximate dimensions 0.666 × 0.156 × 0.121 mm^3) was placed onto a nylon loop with Paratone-N oil, and mounted on an XtaLAB AFC12 (RINC): Kappa dual home/near diffractometer. The data collection was carried out using Cu Kα radiation and the crystal was kept at T = 93 K. A total of 3893 reflections were measured (9.226° $\leq 2\theta \leq$ 156.14°). The structure was solved with SHELXT[5] using direct methods, and refined with SHELXL[6] refinement package of OLEX2[7]. A total of 1616 unique reflections were used in all calculations. The final $R1$ was 0.0615 ($I \geq 2\sigma(I)$) and $wR2$ was 0.1785 (all data). CCDC 2054921 contains the supplementary crystallographic data for this structure.

X-ray Crystallographic Studies on 8. Single crystals of **8** were prepared by slow diffusion of pentane into a $CHCl_3$ solution of this material. An orange crystal (approximate dimensions 0.100 × 0.040 × 0.030 mm^3) was coated with Parabar 10312 (Hampton Research Inc.) to mount on the micro-loop under cold nitrogen stream at T = 100 K. The diffraction data was measured using synchrotron radiation (λ = 0.71073 Å) employing a PLSII-2D SMC on a Rayonix MX225HS CCD area detector with high precision one-axis goniostat at Pohang Accelerator Laboratory, Korea. The PAL BL2D-SMDC program[8] was used for data collection, and HKL3000sm (Ver.717)[9] was used for cell refinement, reduction, and absorption correction. A total of 7574 reflections were measured (3.81° $\leq 2\theta \leq$ 50.996°). The structure was solved with SHELXT[5] using direct methods, and refined with SHELXL[6] refinement package of OLEX2[7]. A total of 2019 unique reflections were used in all calculations. The final $R1$ was 0.0470 ($I \geq 2\sigma(I)$) and $wR2$ was 0.1417 (all data). CCDC 2054918 contains the supplementary crystallographic data for this structure.

X-ray Crystallographic Studies on 10. Single crystals of **10** were prepared by slow diffusion of pentane into a $CHCl_3$ solution of **10**. A red crystal (approximate dimensions 0.596 × 0.261 × 0.186 mm^3) was placed onto a nylon loop with Paratone-N oil, and mounted on an XtaLAB AFC12 (RINC): Kappa dual home/near diffractometer. The data collection was carried out using Cu Kα radiation and the crystal was kept at T = 93 K. A total of 10650 reflections were measured (4.8° $\leq 2\theta \leq$ 158.936°). The structure was solved with SHELXT[5] using direct methods, and refined with SHELXL[6] refinement package of OLEX2[7]. A total of 3851 unique reflections were used in all calculations. The final $R1$ was 0.0650 ($I \geq 2\sigma(I)$) and $wR2$ was 0.1818 (all data). CCDC 2054919 contains the supplementary crystallographic data for this structure.

Hammett Relationship. The term $\Sigma\sigma_p$ in **Supplementary Fig. 9** is the sum of the Hammett constant σ_p of each N-donor group. See **Supplementary Equations 1–8** below. The Hammett constant for each donor group was taken from the reference[10].

$$\Sigma\sigma_p\ (\mathbf{4}) = 2 \times \sigma_p\ (\mathrm{N(COMe)_2}) \tag{1}$$

$$\Sigma\sigma_p\ (\mathbf{5}) = 2 \times \sigma_p\ (\mathrm{NHCOMe}) \tag{2}$$

$$\Sigma\sigma_p\ (\mathbf{6}) = 2 \times \sigma_p\ (\mathrm{NHCOOMe}) \tag{3}$$

$$\Sigma\sigma_p\ (\mathbf{7}) = 2 \times \sigma_p\ (\mathrm{NHCONH_2}) \tag{4}$$

$$\Sigma\sigma_p\ (\mathbf{8}) = \sigma_p\ (\mathrm{NHCOMe}) + \sigma_p\ (\mathrm{NH_2}) \tag{5}$$

$$\Sigma\sigma_p\ (\mathbf{9}) = \sigma_p\ (\mathrm{NHCOOMe}) + \sigma_p\ (\mathrm{NH_2}) \tag{6}$$

$$\Sigma\sigma_p\ (\mathbf{10}) = \sigma_p\ (\mathrm{NHCONH_2}) + \sigma_p\ (\mathrm{NH_2}) \tag{7}$$

$$\Sigma\sigma_p\ (\boldsymbol{p}\textbf{-DAPA}) = 2 \times \sigma_p\ (\mathrm{NH_2}) \tag{8}$$

Lippert–Mataga Equation. Supplementary Fig. 30 was plotted using the Lippert–Mataga equation (**Supplementary Equation 9**), which relates the Stokes shift $\Delta\nu$ with the solvent orientation polarizability Δf.

$$\Delta\nu = \frac{1}{4\pi\varepsilon_0}\frac{2(\mu_\mathrm{e} - \mu_\mathrm{g})^2}{hca^3}\Delta f + constant \tag{9}$$

Here, ε_0 is the dielectric constant of vacuum, h is the Planck constant, c is the speed of light, a is the Onsager cavity radius, and μ_e and μ_g are the dipole moment in the excited and ground states, respectively. The Onsager cavity radii for ***o*-DAPA**, ***p*-DAPA**, and **5** are 4.60, 4.68, and 5.85 Å, respectively, which were calculated with DFT/B3LYP/6-31G* level at MRSF/BH&HLYP/6-31G* optimized geometry.

Theoretical Section

Computational Methodology. Mixed-reference spin-flip (MRSF) not only alleviates the problem of spin-contamination of spin-flip time-dependent density functional theory (SF-TDDFT)[11], but also removes the problematic identification process of spin state. In addition, two completely decoupled linear response equations for singlet and triplet states of MRSF are computationally as efficient as SF-TDDFT. An important advantage of MRSF over the usual linear-response time-dependent density functional theory (LR-TDDFT)[12] is that MRSF enables proper computation of the S_1/S_0 conical intersections (since both states belong to the same response states in MRSF),[13] which is crucial for the accurate description of the dynamics of the excited states. Additionally, MRSF not only includes singly-excited, but also some important doubly-excited configurations in its approximate wavefunctions. The latter is completely missing in the LR-TDDFT, limiting its descriptions of excited states.

Detailed discussion of MRSF-TDDFT can be found elsewhere[14,15]. The main concept is summarized below. Derivation of the MRSF-TDDFT methodology[20] is based on the density-matrix formulation of time-dependent Kohn-Sham theory[16]. In MRSF-TDDFT, the zeroth-order mixed-reference reduced density matrix (MR-RDM), $\rho_0^{\text{MR}}(x, x')$, is defined by satisfying an idempotence relation,

$$\rho_0^{\text{MR}}(x, x') = \int \rho_0^{\text{MR}}(x, x'')\rho_0^{\text{MR}}(x'', x')dx'', \tag{10}$$

and its density, $\rho_0^{\text{MR}}(x) = \rho_0^{\text{MR}}(x, x)$, is same as an equiensemble density of $M_S = +1$ and $M_S = -1$ components of a triplet state, *e.g.*,

$$\rho_0^{\text{MR}}(x) = \frac{1}{2}\left\{\rho_0^{M_S=+1}(x) + \rho_0^{M_S=-1}(x)\right\} \tag{11}$$

Within the Tamm-Dancoff approximation[17,18], the use of MR-RDM in the linear-response formalism yields completely decoupled two linear-response equations for singlet and triplet excited states, respectively[20],

$$\sum_{rs}\left(A_{pq,rs}^{(k)(0)} + A_{pq,rs}^{\prime(k)}\right)X_{rs}^{(k)} = \Omega_{(k)}X_{pq}^{(k)}, \qquad k = S, T \tag{12}$$

where $k = S, T$ labels singlet and triplet states, respectively, $A_{pq,rs}^{(k)(0)}$ is an orbital Hessian matrix derived by the linear response, and $A_{pq,rs}^{\prime(k)}$ is a coupling matrix between configurations originating from different components, $M_S = +1$ and $M_S = -1$, of the mixed reference[20,20]. $X_{pq}^{(k)}$ and $\Omega_{(k)}$ are the amplitude vectors and the excitation energies with respect to the reference state, respectively.

It was shown that a linear response of MRSF-TDDFT can be represented as configurations from $M_S = +1$ and $M_S = -1$ references depicted as those with black and red arrows, respectively, in **Supplementary Fig. 1**[20]. The configurations with the red arrows are missing in the conventional SF-TDDFT; their absence leads to spin contamination of the response states. These are recovered in MRSF-TDDFT, and the spin contamination of the response states is nearly eliminated[20]. Configurations with the blue arrows originate from both $M_S = +1$ and $M_S = -1$ components of the mixed reference. Although not all electronic configurations can be recovered by using the MR-RDM, the missing C $\rightarrow$ V configurations (depicted with gray arrows in **Supplementary Fig. 1**) represent high-lying excited states, and their effect on the lower part of the excitation spectrum is insignificant[20].

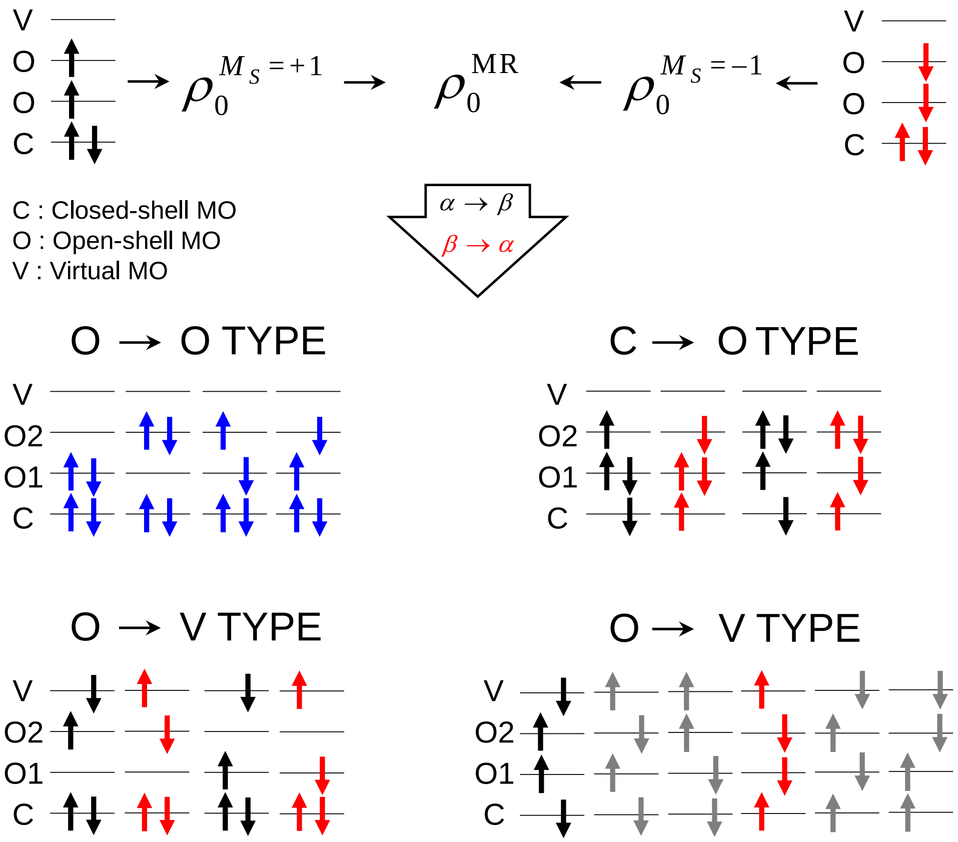

Supplementary Fig. 1. Electronic configurations of the $M_S = +1$ and $M_S = -1$ components of triplet reference in the upper panel, and a complete set of configurations for MRSF-TDDFT in the lower panel. Response states are described by configurations represented with blue, black, and red arrows in MRSF-TDDFT, while with blue and black arrows in SF-TDDFT.

Quantum Theory Atoms In Molecules (QTAIM) analysis. QTAIM analysis (in **Supplementary Table 9**) is an effective tool to describe the nature of intra- and intermolecular hydrogen bonds. Bond critical points (BCP) are the special point along the bond path at the interatomic surface, where the shared electron density $\rho(\mathrm{r})$ reaches a minimum.

We analyzed the topology of $\rho(\mathrm{r})$ of BCP between the hydrogen atom of amine group and the oxygen atom of acetyl group at MRSF/BH&HLYP/6-31G* optimized geometry. The wavefunctions obtained from MRSF were used to calculate the density $\rho(\mathrm{r})$, its Laplacian $\nabla^2\rho(\mathrm{r})$, and the potential energy density $V(\mathrm{r})$ at BCP using Multiwfn program[19]. The hydrogen bondenergy was estimated from $\rho(\mathrm{r})$[20], according to eq. (5).

$$E_{HB} \approx -223.08 \times \rho(r) + 0.7423 \tag{13}$$

Supplementary Table 1. Summary of X-ray crystallographic data for ***o*-DAPA**, ***m*-DAPA**, ***p*-DAPA**, and **4**.

	***o*-DAPA**	***m*-DAPA**	***p*-DAPA**	**4**
Chemical formula	$C_{10}H_{12}N_2O_2$	$C_{10}H_{12}N_2O_2$	$C_{10}H_{12}N_2O_2$	$C_{24}H_{24}N_2O_6$
Formula weight	192.22	192.22	192.22	436.45
Crystal system	monoclinic	trigonal	monoclinic	monoclinic
Space group	$P2_1/n$	$R\bar{3}$	$P2_1/c$	$P2_1/n$
Color of crystal	Orange	Colorless	Red	Colorless
a (Å)	8.14040(10)	28.2823(8)	10.2867(4)	10.45020(10)
b (Å)	26.4898(3)	28.2823(8)	11.2031(5)	10.63510(10)
c (Å)	8.63020(10)	6.8776(3)	7.8865(3)	19.8214(2)
α (°)	-	-	-	-
β (°)	101.7910(10)	-	95.061(4)	93.0590(10)
γ (°)	-	-	-	-
Volume (Å^3)	1821.73(4)	4764.3(3)	905.32(6)	2199.79(4)
Z	4	18	4	4
R(int)	0.0358	0.0464	0.0368	0.0286
Final R indices [$I > 2\sigma(I)$]	$R1 = 0.0568$, $wR2 = 0.1737$	$R1 = 0.0481$, $wR2 = 0.1249$	$R1 = 0.0814$, $wR2 = 0.2680$	$R1 = 0.0368$, $wR2 = 0.0947$
Final R indices [all data]	$R1 = 0.0604$, $wR2 = 0.1805$	$R1 = 0.0562$, $wR2 = 0.1291$	$R1 = 0.0864$, $wR2 = 0.2716$	$R1 = 0.0386$, $wR2 = 0.0963$
GOF	1.085	1.091	1.139	1.046

Supplementary Table 2. Summary of X-ray crystallographic data for **5**, **6**, **7**, **8**, and **10**.

	**5**	**6**	**7**	**8**	**10**
Chemical formula	$C_{14}H_{16}N_2O_4$	$C_{20}H_{28}N_2O_6$	$C_8H_{11}N_2O_2$	$C_{12}H_{14}N_2O_3$	$C_{13}H_{17}N_3O_3{\cdot}CHCl_3$
Formula weight	276.29	392.44	167.19	234.25	382.66
Crystal system	monoclinic	monoclinic	monoclinic	monoclinic	trigonal
Space group	$C2/c$	$P2_1/c$	$P2_1/n$	$P2_1/n$	$R\bar{3}$
Color of crystal	Yellow	Orange	Orange	Green	Red
a (Å)	24.022(5)	6.80930(10)	4.46070(10)	4.9140(10)	36.8131(3)
b (Å)	4.0160(8)	23.0855(3)	19.1723(4)	13.817(3)	36.8131(3)
c (Å)	17.877(4)	13.1682(2)	9.5290(2)	16.978(3)	7.01180(10)
α (°)	-	-	-	-	-
β (°)	129.80(3)	91.3920(10)	90.119(2)	96.36(3)	-
γ (°)	-	-	-	-	-
Volume (Å^3)	1325.0(6)	2069.38(5)	814.94(3)	1145.7(4)	8229.33(18)
Z	4	4	4	4	18
R(int)	0.0686	0.0366	0.0368	0.0722	0.0568
Final R indices [$I > 2\sigma(I)$]	$R1 = 0.0990$, $wR2 = 0.2570$	$R1 = 0.0425$, $wR2 = 0.1088$	$R1 = 0.0615$, $wR2 = 0.1748$	$R1 = 0.0470$, $wR2 = 0.1380$	$R1 = 0.0650$, $wR2 = 0.1759$
Final R indices [all data]	$R1 = 0.1394$, $wR2 = 0.2960$	$R1 = 0.0490$, $wR2 = 0.1129$	$R1 = 0.0636$, $wR2 = 0.1785$	$R1 = 0.0503$, $wR2 = 0.1417$	$R1 = 0.0676$, $wR2 = 0.1818$
GOF	0.996	1.076	1.079	1.105	1.063

Supplementary Table 3. Summary of fluorophores listed in **Supplementary Figure 2**.

Entry	Structure (Formula)	FW	$\lambda_{max,abs}$	$\lambda_{max,em}$	Reference
1	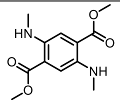 ($C_{12}H_{16}N_2O_4$)	252.27	484	620 (film)	*Angew. Chem. Int. Ed.* **56**, 12543–12547 (2017).
2	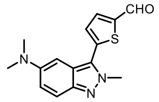 ($C_{15}H_{15}N_3OS$)	285.365	436	624	*J. Am. Chem. Soc.* **138**, 4730–4738 (2016).
3	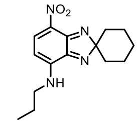 ($C_{15}H_{20}N_4O_2$)	288.351	546	650	*Angew. Chem. Int. Ed.* **58**, 6911–6915 (2019).
4	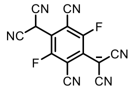 ($C_{14}F_2N_6$)	291.20	505	654	*Chem. Commun.* **55**, 11462–11465 (2019).
5	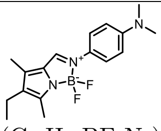 ($C_{17}H_{22}BF_2N_3$)	317.191	458	650	*Chem. Eur. J.* **22**, 17321–17328 (2016).
6	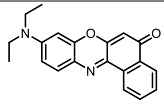 ($C_{20}H_{18}N_2O_2$)	318.376	552	636	Nile Red
7	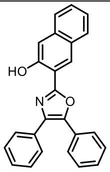 ($C_{25}H_{17}NO_2$)	363.416	333	628	*J. Am. Chem. Soc.* **135**, 11239–11246 (2013).
8	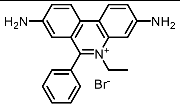 ($C_{21}H_{20}BrN_3$)	394.316	520	600	Ethidium Bromide
9	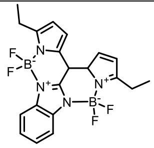 ($C_{20}H_{20}B_2F_4N_4$)	414.022	598	612	*Angew. Chem. Int. Ed.* **55**, 13340–13344 (2016).
10	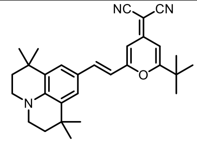 ($C_{30}H_{35}N_3O$)	453.63	502	602	DCJTB

11	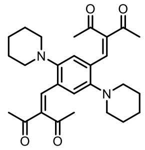 ($C_{28}H_{36}N_2O_4$)	464.606	425	656	*Angew. Chem. Int. Ed.* **48**, 3653–3656 (2009).
12	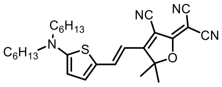 ($C_{28}H_{36}N_4OS$)	476.683	614	646	*Acc. Chem. Res.* **38**, 549–556 (2005).
13	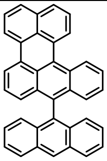 ($C_{38}H_{22}$)	478.594	568	608	*J. Mater. Chem.* **12**, 1307–1310 (2002).
14	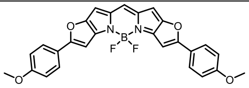 ($C_{27}H_{19}BF_2N_2O_4$)	484.266	673	683	*J. Am. Chem. Soc.* **130**, 1550–1551 (2008).
15	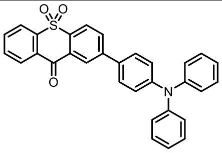 ($C_{31}H_{21}NO_3S$)	487.573	450	625 (film)	*Adv. Mater.* **26**, 5198–5204 (2014).
16	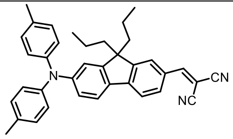 ($C_{37}H_{35}N_3$)	521.708	516	657	*Adv. Funct. Mater.* **15**, 231–238 (2005).
17	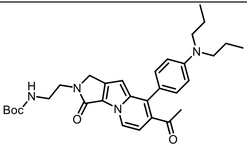 ($C_{31}H_{40}N_4O_4$)	532.685	445	605	*J. Am. Chem. Soc.* **133**, 6642–6649 (2011).
18	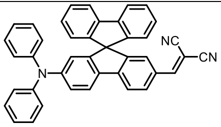 ($C_{41}H_{25}N_3$)	559.672	-	624	*Adv. Funct. Mater.* **15**, 232–238 (2005).
19	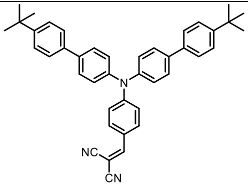 ($C_{42}H_{39}N_3$)	585.795	447	631	*Chem. Mater.* **20**, 6597–6599 (2008).
20	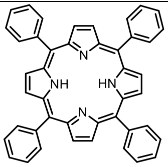 ($C_{44}H_{30}N_4$)	614.752	-	650	*J. Am. Chem. Soc.* **94**, 3986–3992 (1972).

21	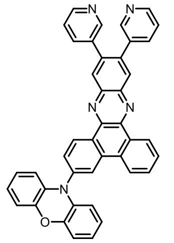 ($C_{42}H_{25}N_5O$)	615.696	450	607	*Angew. Chem. Int. Ed.* **58**, 14660–14665 (2009).
22	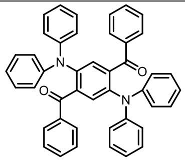 ($C_{44}H_{32}N_2O_2$)	620.752	450	602	*Tetrahedron Lett.* **52**, 4084–4089 (2011).
23	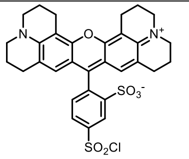 ($C_{31}H_{29}ClN_2O_6S_2$)	625.151	596	615	Texas Red
24	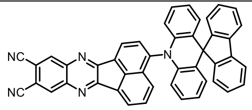 ($C_{45}H_{23}N_5$)	633.714	-	647	*Adv. Funct. Mater.* **30**, 1908839 (2020).
25	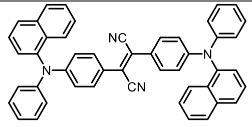 ($C_{48}H_{32}N_4$)	664.812	-	616	*Chem. Commun.* **20**, 2632–2633 (2003).
26	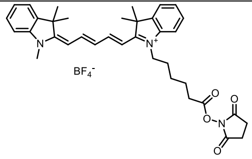 ($C_{36}H_{42}BF_4N_3O_4$)	667.553	646	662	Cy5
27	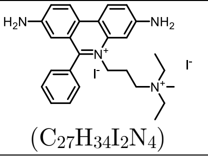 ($C_{27}H_{34}I_2N_4$)	668.406	535	617	Propidium Iodide
28	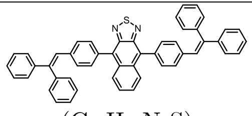 ($C_{50}H_{34}N_2S$)	694.896	499	620 (film)	*Adv. Mater.* **18**, 1607–1611 (2006).
29	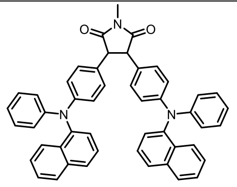 ($C_{49}H_{37}N_3O_2$)	699.854	-	650 (film)	*Adv. Mater.* **14**, 1072–1075 (2002).
30	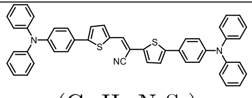 ($C_{47}H_{33}N_3S_2$)	703.922	482	635 (film)	*J. Am. Chem. Soc.* **141**, 15111–15120 (2019).

31	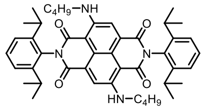 ($C_{46}H_{56}N_4O_4$)	728.978	621	650	*Chem. Eur. J.* **8**, 4742–4750 (2002).
32	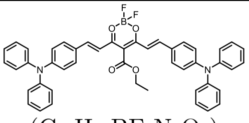 ($C_{46}H_{37}BF_2N_2O_4$)	730.618	-	721 (film)	*Nat. Photonics* **12**, 98 (2018).
33	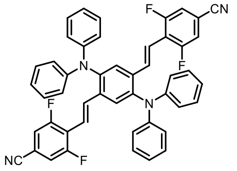 ($C_{48}H_{30}F_4N_4$)	738.790	473	609	*Angew. Chem. Int. Ed.* **124**, 4171–4175 (2012).
34	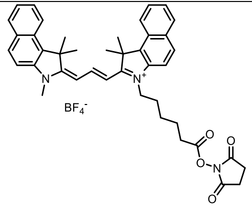 ($C_{42}H_{44}BF_4N_3O_4$)	741.635	591	604	Cy3.5
35	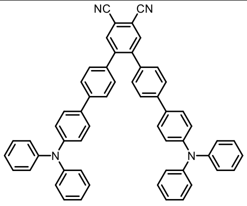 ($C_{56}H_{38}N_4$)	766.948	-	631 (film)	*Adv. Funct. Mater.* **15**, 1541–1546 (2005).
36	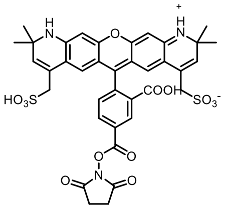 ($C_{37}H_{33}N_3O_{13}S_2$)	791.799	578	602	Thermo Fisher - Alexa Fluor^{TM} 568
37	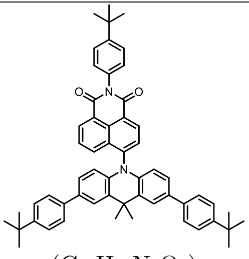 ($C_{57}H_{56}N_2O_2$)	801.087	-	622 (film)	*Adv. Mater.* **31**, 1901404 (2019).
38	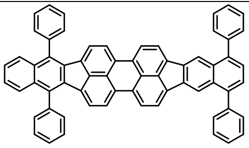 ($C_{64}H_{36}$)	804.992	333	610	DBP

39	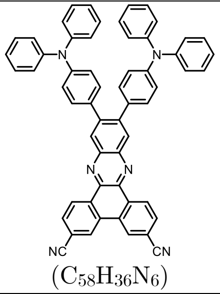 ($C_{58}H_{36}N_6$)	816.968	488	628 (film)	*Adv. Mater.* **31**, 1902368 (2019).
40	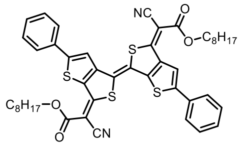 ($C_{46}H_{46}N_2O_4S_4$)	819.124	605	640	*J. Am. Chem. Soc.* **137**, 11294−11302 (2015).
41	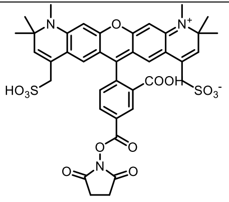 ($C_{39}H_{37}N_3O_{13}S_2$)	819.853	590	617	Thermo Fisher - Alexa Fluor™ 594
42	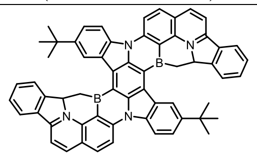 ($C_{60}H_{46}B_2N_4$)	844.676	597	615	*J. Am. Chem. Soc.* **142**, 19468−19472 (2020).
43	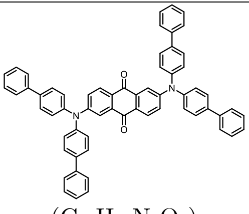 ($C_{62}H_{42}N_2O_2$)	847.03	464	629	*J. Am. Chem. Soc.* **136**, 18070−18081 (2014).
44	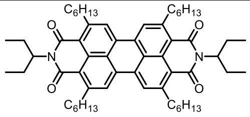 ($C_{58}H_{78}N_2O_4$)	867.272	521	635 (film)	*Chem. Eur. J.* **15**, 7530–7533 (2009).
45	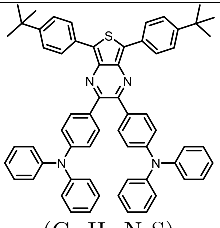 ($C_{62}H_{54}N_4S$)	887.202	455	633	*Adv. Mater.* **14**, 822–826 (2002).
46	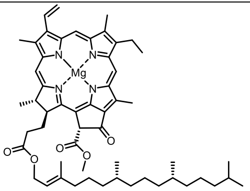 ($C_{55}H_{72}MgN_4O_5$)	893.509	661	666	Chlorophyll A

47	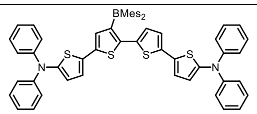 ($C_{58}H_{49}BN_2S_4$)	913.094	479	660	*Angew. Chem. Int. Ed.* **46**, 4273–4276 (2007).
48	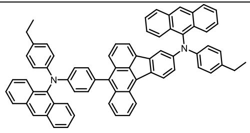 ($C_{70}H_{50}N_2$)	919.184	520	634	*Chem. Mater.* **15** 4854–4862 (2003).
49	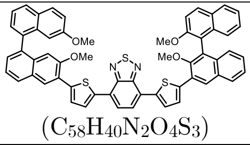 ($C_{58}H_{40}N_2O_4S_3$)	925.148	-	661	*Adv. Funct. Mater.* **18**, 3299–3306 (2008).
50	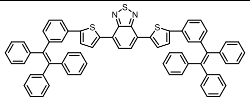 ($C_{66}H_{44}N_2S_3$)	961.272	510	623	*Chem. Commun.* **47**, 8847–8849 (2011).
51	DyLight™ 633	1066	638	658	Thermo Fisher
52	DyLight™ 650	1066	652	672	Thermo Fisher
53	DyLight™ 594	1078	593	618	Thermo Fisher
54	Alexa Fluor™ 660**	1100	668	698	Thermo Fisher
55	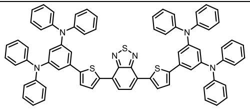 ($C_{74}H_{52}N_6S_3$)	1121.452	489	632	*Adv. Funct. Mater.* **14**, 83–90 (2004).
56	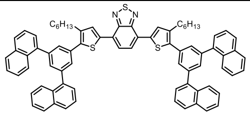 ($C_{78}H_{64}N_2S_3$)	1125.564	486	613	*Adv. Mater.* **20**, 4172–4175 (2008).
57	Alexa Fluor™ 633**	1200	621	639	Thermo Fisher
58	Alexa Fluor™ 647**	1250	651	672	Thermo Fisher
59	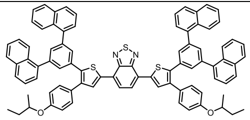 ($C_{86}H_{64}N_2O_2S_3$)	1253.65	491	609	*Adv. Funct. Mater.* **19**, 2978–2986 (2009).
60	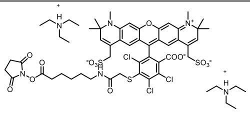 ($C_{58}H_{77}Cl_3N_6O_{14}S_3$)	1284.812	603	623	Thermo Fisher - Alexa Fluor™ 610-X

Supplementary Table 4. Energies (in eV) calculated for the optimized geometries of ***o*-DAPA** in S_0, S_1, and S_2 in gas phase at the MRSF/BH&HLYP/6-31G* level.

	FC	$S_{1,\ \mathrm{min}}$	$S_{1,\ \mathrm{2pt}}$	CI_{10}	$CI_{10,\ \mathrm{1pt}}$
S_0	0.00	0.41	2.05	4.39	4.74
S_1	3.73	3.33	4.29	4.39	4.74
S_2	4.70	4.69	5.58	6.35	7.08

Supplementary Table 5. Energies (in eV) calculated for the optimized geometries of ***m*-DAPA** in S_0, S_1, and S_2 in gas phase at the MRSF/BH&HLYP/6-31G* level.

	FC	CI_{21}	$S_{1,\ \mathrm{1pt}}$	$S_{1,\ \mathrm{2pt}}$	$CI_{10,\ \mathrm{1pt}}$	$CI_{10,\ \mathrm{2pt}}$
S_0	0.00	0.54	1.77	3.14	4.08	3.84
S_1	4.66	4.50	3.72	3.71	4.08	3.84
S_2	4.67	4.50	5.25	5.62	5.32	5.95

Supplementary Table 6. Energies (in eV) calculated for the optimized geometries of ***p*-DAPA** in S_0, S_1, and S_2 in gas phase at the MRSF/BH&HLYP/6-31G* level.

	FC	$S_{1,\ \mathrm{min}}$	$S_{1,\ \mathrm{1pt}}$	$CI_{10,\ \mathrm{1pt}}$	$CI_{10,\ \mathrm{2pt}}$
S_0	0.00	0.28	1.48	3.62	3.91
S_1	3.25	2.85	3.40	3.62	3.91
S_2	4.95	4.59	5.02	6.20	6.48

Supplementary Table 7. Calculated HOMO and LUMO energies (in eV) at the MRSF/BH&HLYP/6-31G* level.

	***o*-DAPA**	***m*-DAPA**	***p*-DAPA**	**5**	**6**	**7**	**8**	**9**	**10**
HOMO	–5.56	–5.69	–5.14	–5.95	–5.74	–5.50	–5.53	–5.43	–5.39
LUMO	–1.87	–0.91	–1.86	–2.27	–2.08	–1.98	–2.05	–1.96	–1.92
Gap	3.70	4.78	3.28	3.68	3.67	3.53	3.47	3.47	3.39

Supplementary Table 8. Excitation energies, transition dipole moments, and oscillator strengths for the S_0-to-S_1 transitions of ***o*-DAPA**, ***p*-DAPA**, and **5–10**, calculated at the MRSF/BH&HLYP/6-31G* level of theory.

	***o*-DAPA**	***p*-DAPA**	**5**	**6**	**7**	**8**	**9**	**10**
Energy (eV)	3.731	3.246	3.706	3.685	3.523	3.432	3.435	3.351
μ (D)	1.851	1.916	1.959	1.919	1.942	1.915	1.901	1.914
f	0.3133	0.2921	0.3484	0.3324	0.3255	0.3081	0.3043	0.3006

Supplementary Table 9. Emission energies, transition dipole moments, and oscillator strengths for the S_0-to-S_1 transitions of ***o*-DAPA**, ***p*-DAPA**, and **5–10**, calculated at the MRSF/BH&HLYP/6-31G* level of theory.

	***o*-DAPA**	***p*-DAPA**	**5**	**6**	**7**	**8**	**9**	**10**
Energy (eV)	2.924	2.576	3.036	3.034	2.890	2.780	2.789	2.704
μ (D)	1.856	2.055	2.039	2.010	2.025	2.020	2.009	2.020
f	0.2467	0.2662	0.3091	0.3003	0.2905	0.2778	0.2759	0.2704

Supplementary Table 10. HOMA values for the optimized geometries of ***o*-DAPA**, ***p*-DAPA**, and **5–10** at $S_{0,min}$ and $S_0@S_{1,min}$.

	***o*-DAPA**	***p*-DAPA**	**5**	**6**	**7**	**8**	**9**	**10**
$S_{0,min}$	0.874	0.938	0.947	0.948	0.937	0.939	0.941	0.936
$S_0@S_{1,min}$	0.599	0.745	0.703	0.709	0.704	0.713	0.720	0.716

Supplementary Table 11. Selected parameters for N–H···O hydrogen bonds obtained by QTAIM analysis.

	geometry	$d_{\mathrm{N-H\cdots O}}$, (Å)	ρ(r), (arb.units)	$\nabla^2\rho$(r), (arb.units)	V(r), (arb.units)	E_{HB}, (kcal mol^{-1})	E_{HB}, (eV)
***o*-DAPA**	FC	1.896	0.0325	0.111	–0.0291	–6.52	–0.283
	$S_{1,\mathrm{min}}$	1.821	0.0379	0.128	–0.0341	–7.71	–0.334
***p*-DAPA**	FC	1.933	0.0302	0.104	–0.0270	–5.99	–0.260
	$S_{1,\mathrm{min}}$	1.796	0.0398	0.137	–0.0361	–8.14	–0.353
**5**	FC	1.814	0.0384	0.131	–0.0347	–7.82	–0.339
	$S_{1,\mathrm{min}}$	1.691	0.0502	0.170	–0.0467	–10.45	–0.453
**6**	FC	1.832	0.0367	0.126	–0.0331	–7.45	–0.323
	$S_{1,\mathrm{min}}$	1.707	0.0480	0.164	–0.0443	–10.72	–0.465
**7**	FC	1.798	0.0397	0.135	–0.0361	–8.12	–0.352
	$S_{1,\mathrm{min}}$	1.679	0.0516	0.174	–0.0482	–10.8	–0.468
**8**	FC (NHAc)	1.823	0.0377	0.128	–0.0342	–7.68	–0.333
	FC (NH_2)	1.922	0.0308	0.107	–0.0275	–6.13	–0.266
	$S_{1,\mathrm{min}}$ (NHAc)	1.691	0.0503	0.170	–0.0467	–10.47	–0.454
	$S_{1,\mathrm{min}}$ (NH_2)	1.794	0.0400	0.138	–0.0363	–8.17	–0.354
**9**	FC (NHBoc)	1.840	0.0361	0.124	–0.0325	–7.31	–0.317
	FC (NH_2)	1.923	0.0148	0.0992	–0.0151	–2.56	–0.111
	$S_{1,\mathrm{min}}$ (NHBoc)	1.709	0.0479	0.163	–0.0440	–9.94	–0.431
	$S_{1,\mathrm{min}}$ (NH_2)	1.792	0.0401	0.138	–0.0364	–8.21	–0.356
**10**	FC (NHDmc)	1.805	0.0391	0.133	–0.0355	–8.73	–0.389
	FC (NH_2)	1.926	0.0306	0.106	–0.0273	–6.08	–0.264
	$S_{1,\mathrm{min}}$ (NHDmc)	1.679	0.0516	0.174	–0.0482	–10.77	–0.467
	$S_{1,\mathrm{min}}$ (NH_2)	1.800	0.0395	0.136	–0.0358	–8.07	–0.350

Supplementary Table 12. Photophysical properties of ***o*-DAPA**, ***p*-DAPA**, **5**, and **8** in different solvents.

		$\lambda_{abs,max}$ (nm)	$\lambda_{em,max}$ (nm)	Stokes shift (cm^{-1})	ϕ_F (%)
***o*-DAPA**	Cyclohexane	425	515	4110	32
	Toluene	433	528	4160	33
	$CHCl_3$	432	531	4320	26
	Et_2O	439	528	3840	26
	THF	443	533	3810	29
	EtOAc	440	529	3820	24
	DCM	428	530	4500	30
	DMSO	452	549	3910	26
	EtOH	450	546	3910	6
	MeCN	437	533	4120	20
	H_2O	435[b]	572	5510[b]	-[a]
***p*-DAPA**	Cyclohexane	482	590	3800	16
	Toluene	483	609	4280	11
	$CHCl_3$	482	618	4570	6
	Et_2O	493	617	4080	7
	THF	496	623	4110	5
	EtOAc	488	625	4490	5
	DCM	479	616	4640	7
	DMSO	496	655	4890	3
	EtOH	482	668	5780	-[a]
	MeCN	481	633	4990	4
	H_2O	454[b]	693	7600[b]	-[a]
**5**	Cyclohexane	409	496	4290	61
	Toluene	411	503	4450	70
	$CHCl_3$	405	502	4770	73
	Et_2O	407	496	4410	63
	THF	404	502	4830	63
	EtOAc	404	503	4870	73
	DCM	408	502	4590	72
	DMSO	371[b]	508	7270[b]	44
	EtOH	386[b]	515	6490[b]	61
	MeCN	397	503	5310	56
	H_2O	355[b]	516	8790[b]	28
**8**	Cyclohexane	442	535	3930	39
	Toluene	445	548	4220	35
	$CHCl_3$	445	556	4490	30
	Et_2O	449	547	3990	27
	THF	450	556	4240	25
	EtOAc	445	555	4450	24
	DCM	441	551	4530	36
	DMSO	408[b]	554	6460[b]	30
	EtOH	425[b]	572	6050[b]	7
	MeCN	442	558	4700	21
	H_2O	395[b]	574	7890[b]	2

[a]Negligible fluorescence intensity. [b]Indefinite due to the broadening in the absorption spectrum.

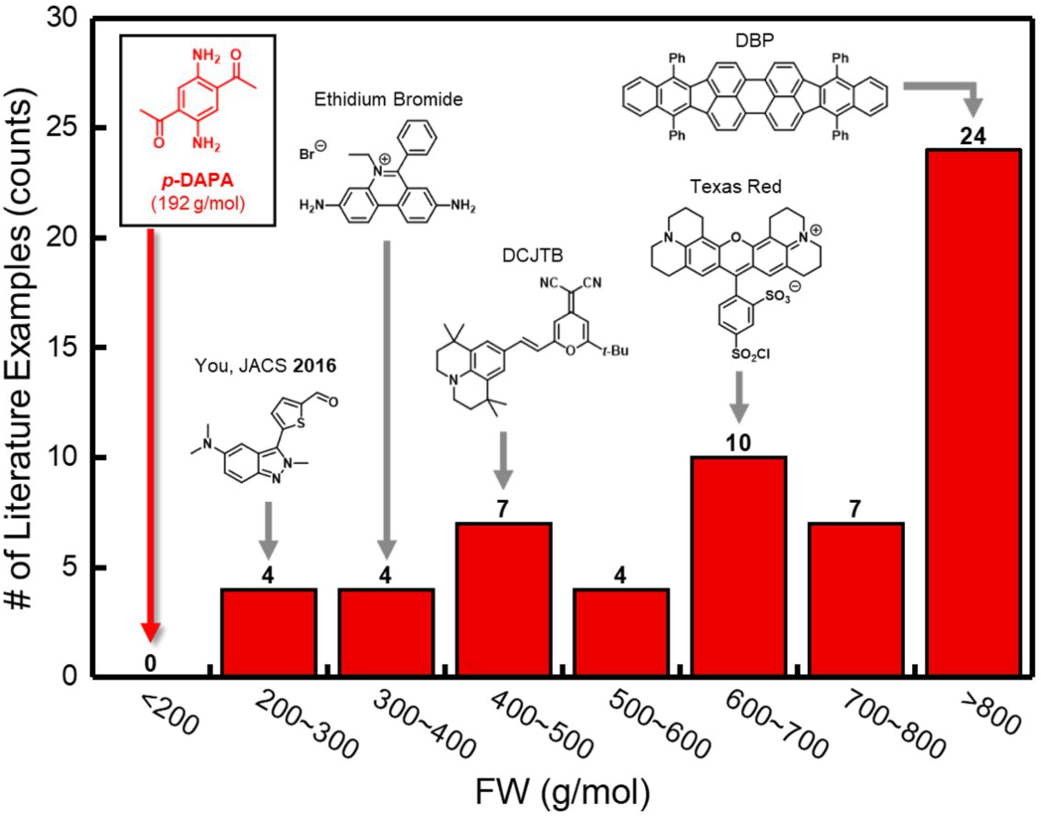

Supplementary Fig. 2. Molecular weight distribution of selected red fluorophores ($\lambda_{\mathrm{max,em}}$ = 600–700 nm). See **Supplementary Table 3** for the complete list of sixty molecules used in this analysis.

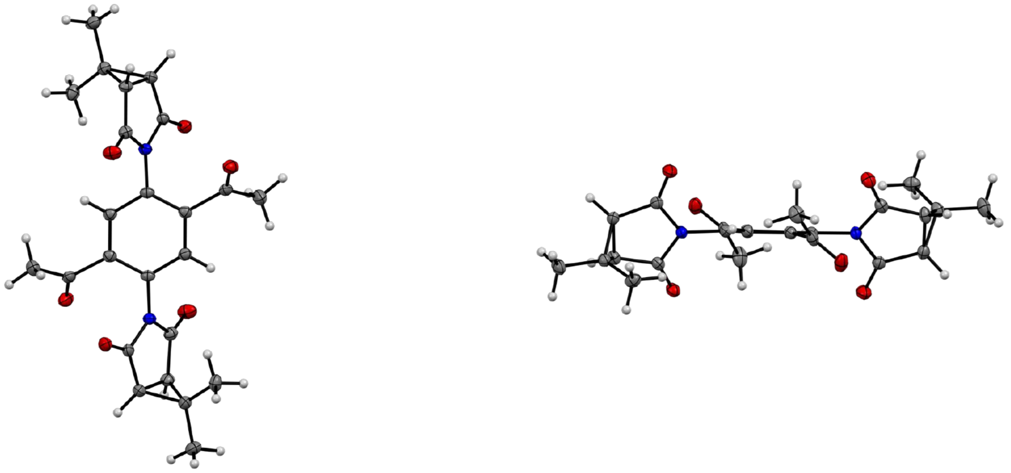

Supplementary Fig. 3. ORTEP diagrams of **4** (left, face-on view; right, edge-on view) with thermal ellipsoids at the 50% probability level.

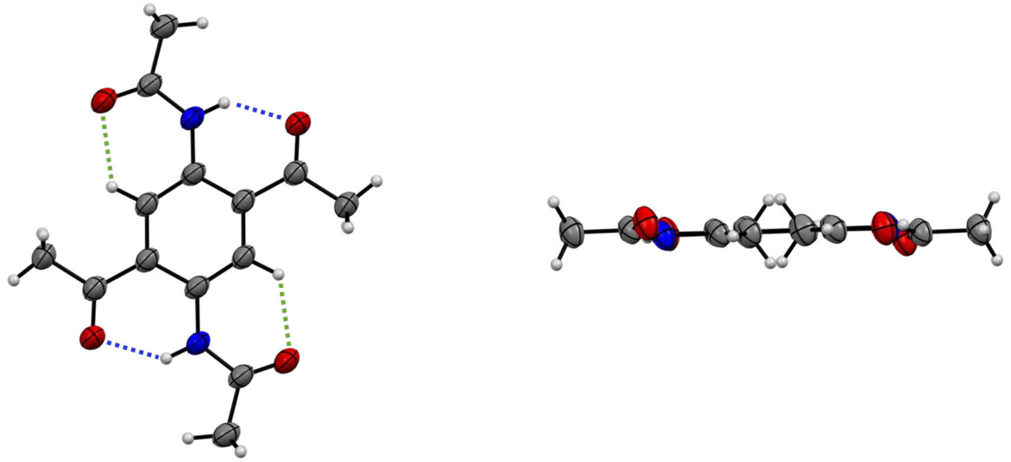

Supplementary Fig. 4. ORTEP diagrams of **5** (left, face-on view; right, edge-on view) with thermal ellipsoids at the 50% probability level. N–H$\cdots$O hydrogen bonds ($d_{\mathrm{N}\cdots\mathrm{O}}$ = 2.648(3) Å) are indicated by blue dotted lines, and C–H$\cdots$O hydrogen bonds ($d_{\mathrm{C}\cdots\mathrm{O}}$ = 2.837(4) Å) by green dotted lines.

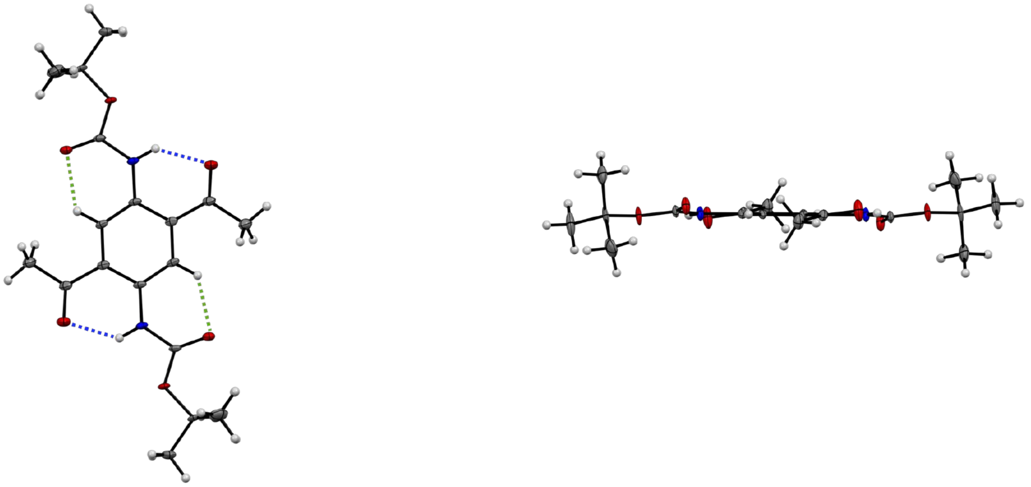

Supplementary Fig. 5. ORTEP diagrams of **6** (left, face-on view; right, edge-on view) with thermal ellipsoids at the 50% probability level. N–H···O hydrogen bonds ($d_{N\cdots O}$ = 2.636(1) Å) are indicated by blue dotted lines, and C–H···O hydrogen bonds ($d_{C\cdots O}$ = 2.823(2) Å) by green dotted lines.

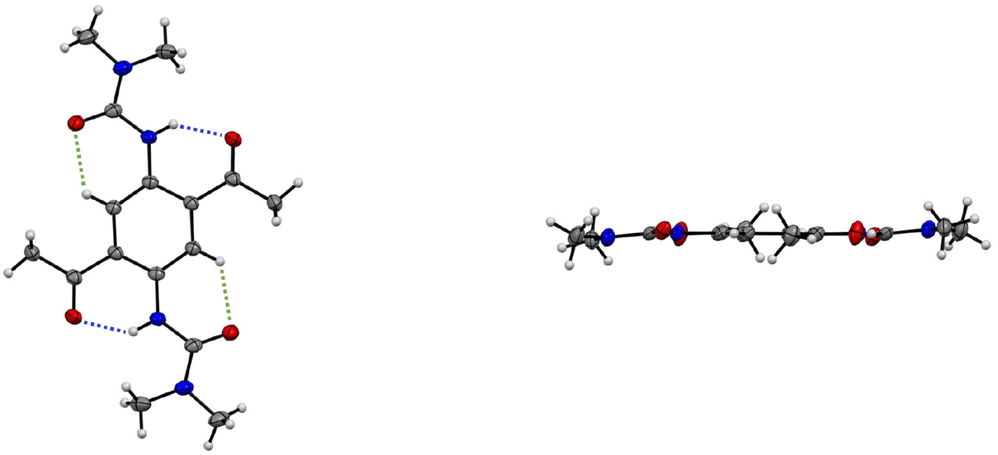

Supplementary Fig. 6. ORTEP diagrams of **7** (left, face-on view; right, edge-on view) with thermal ellipsoids at the 50% probability level. N–H···O hydrogen bonds ($d_{N\cdots O}$ = 2.621(2) Å) are indicated by blue dotted lines, and C–H···O hydrogen bonds ($d_{C\cdots O}$ = 2.855(2) Å) by green dotted lines.

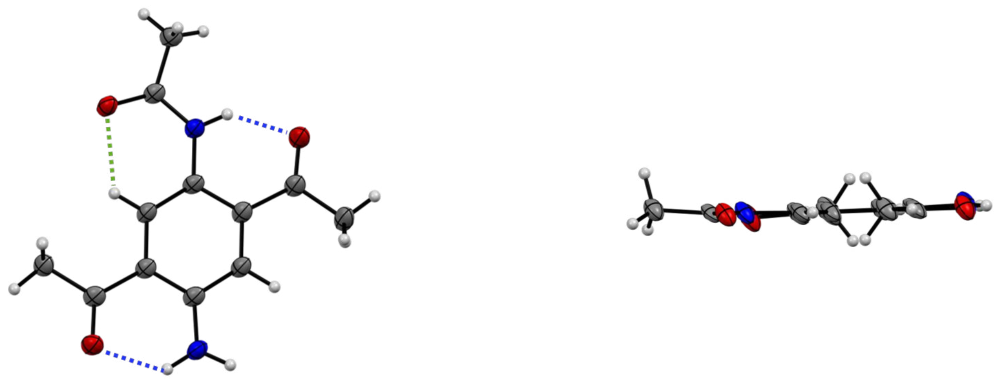

Supplementary Fig. 7. ORTEP diagrams of **8** (left, face-on view; right, edge-on view) with thermal ellipsoids at the 50% probability level. N–H$\cdots$O hydrogen bonds ($d_{\mathrm{N\cdots O}}$ = 2.662(2) Å and 2.663(2) Å) are indicated by blue dotted lines, and C–H$\cdots$O hydrogen bonds ($d_{\mathrm{C\cdots O}}$ = 2.878(2) Å) by green dotted lines.

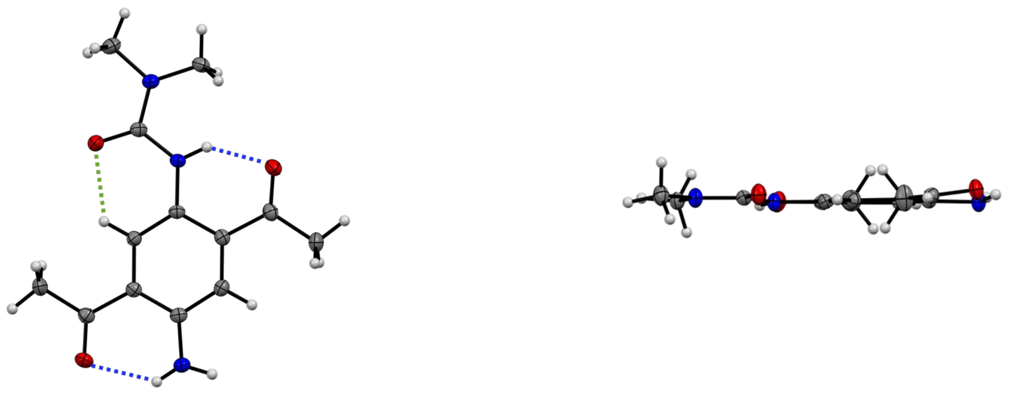

Supplementary Fig. 8. ORTEP diagrams of **10** (left, face-on view; right, edge-on view) with thermal ellipsoids at the 50% probability level. N–H$\cdots$O hydrogen bonds ($d_{\mathrm{N\cdots O}}$ = 2.635(3) Å and 2.692(3) Å) are indicated by blue dotted lines, and C–H$\cdots$O hydrogen bonds ($d_{\mathrm{C\cdots O}}$ = 2.830(2) Å) by green dotted lines.

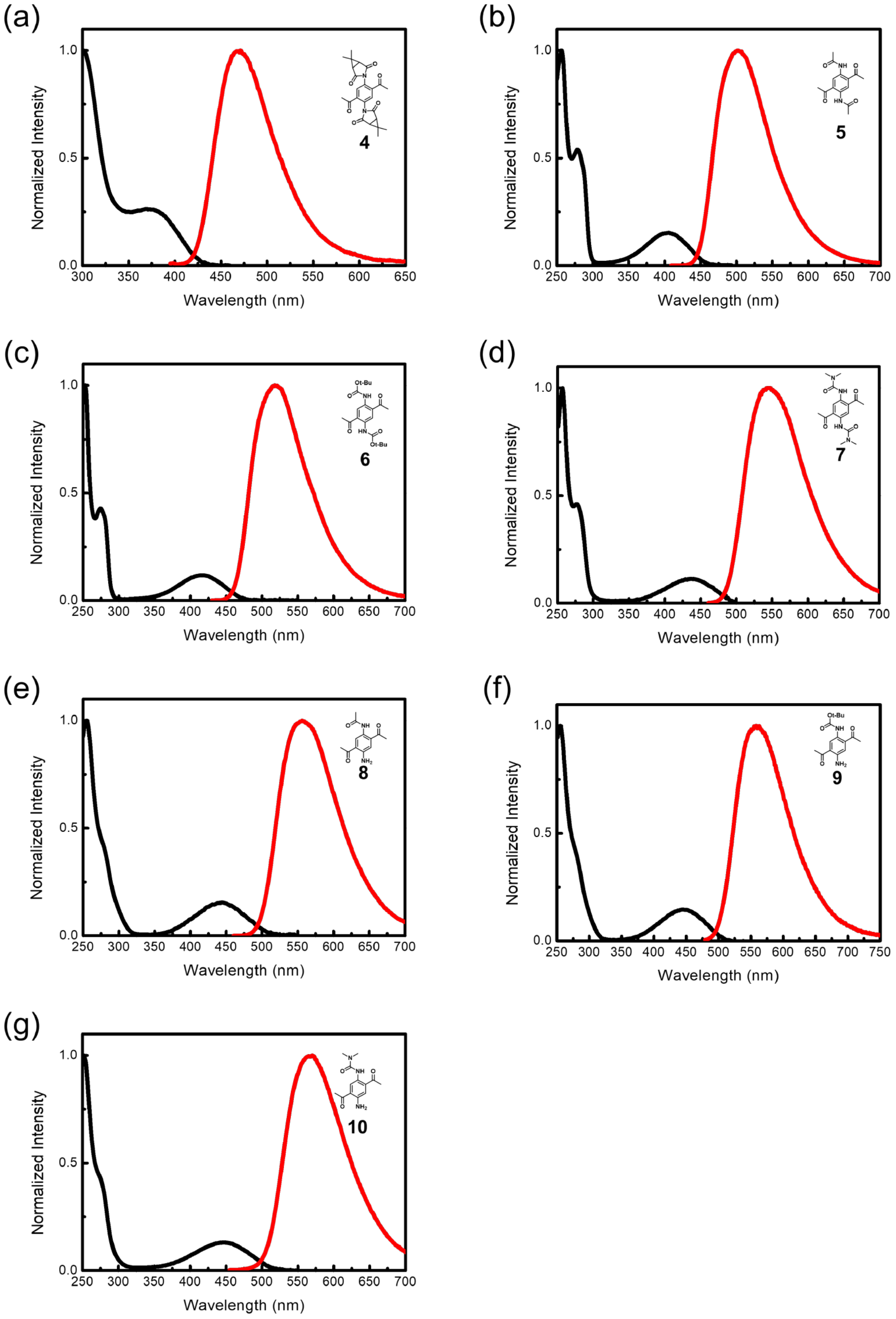

Supplementary Fig. 9. Normalized absorption (black) and emission (red) spectra of (a) **4**, (b) **5**, (c) **6**, (d) **7**, (e) **8**, (f) **9**, and (g) **10** in $CHCl_3$. T = 298 K.

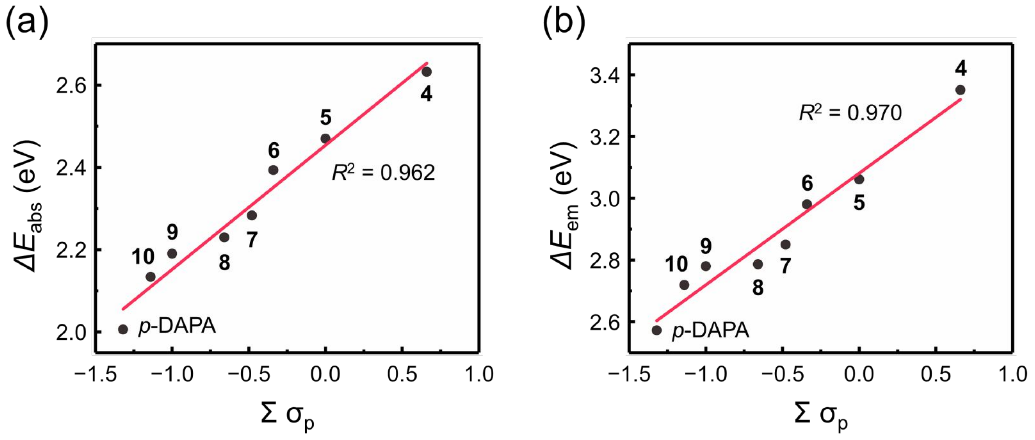

Supplementary Fig. 10. Linear free energy relationship (LFER) between $\Sigma\sigma_p$ values and experimentally determined (a) absorption, or (b) emission energy. See page S-8 for details.

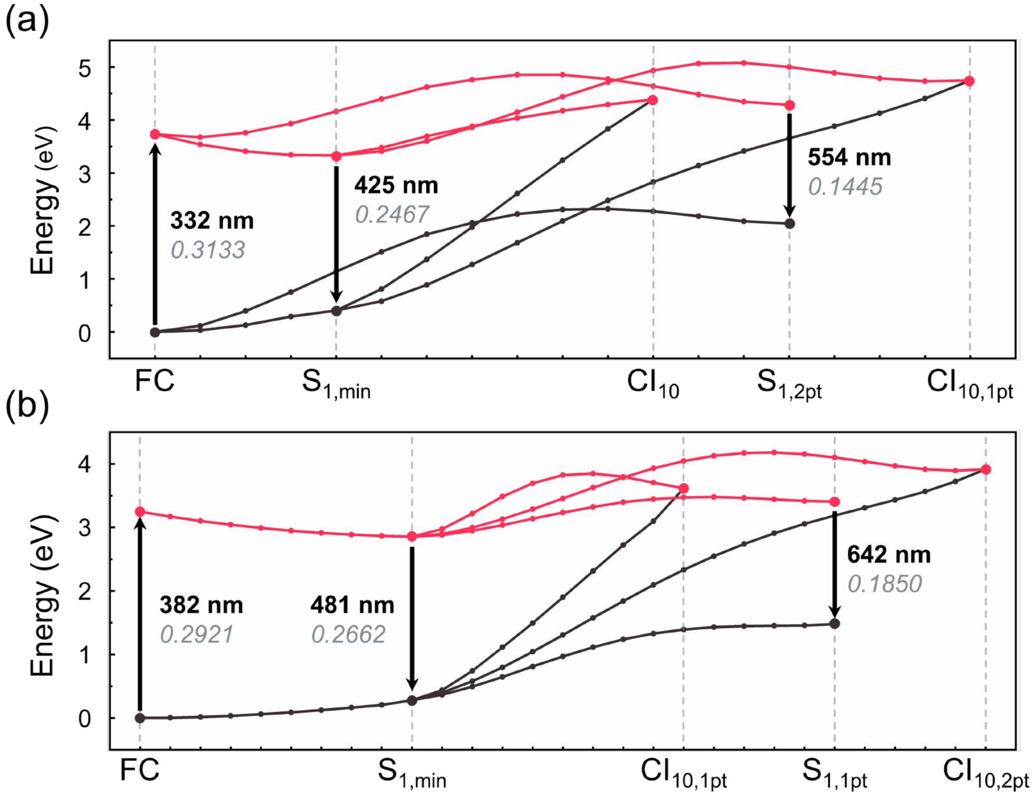

Supplementary Fig. 11. Calculated S_0 (black) and S_1 (red) energy profiles of (a) ***o*-DAPA** and (b) ***p*-DAPA** at the MRSF/BH&HLYP/6-31G* level of theory. Relative energies are plotted verses the reaction coordinates obtained by the geodesic interpolation method. For each transition, calculated wavelengths and oscillator strengths are shown in bold and italic, respectively.

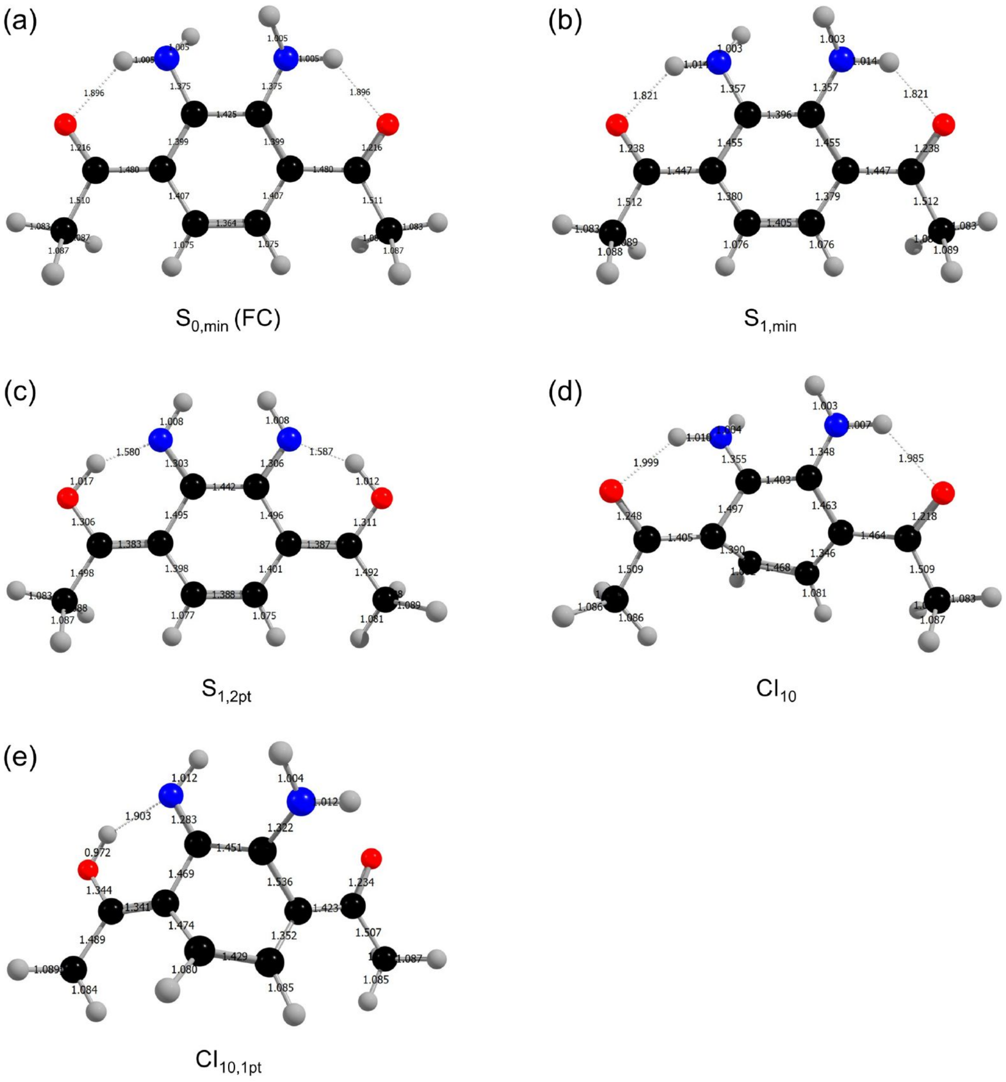

Supplementary Fig. 12. Optimized geometries of ***o*-DAPA** at (a) $S_{0,\ \mathrm{min}}$ (FC), (b) $S_{1,\ \mathrm{min}}$, (c) $S_{1,\ \mathrm{2pt}}$, (d) CI_{10}, and (e) $CI_{10,\ \mathrm{1pt}}$ with bond lengths in Å.

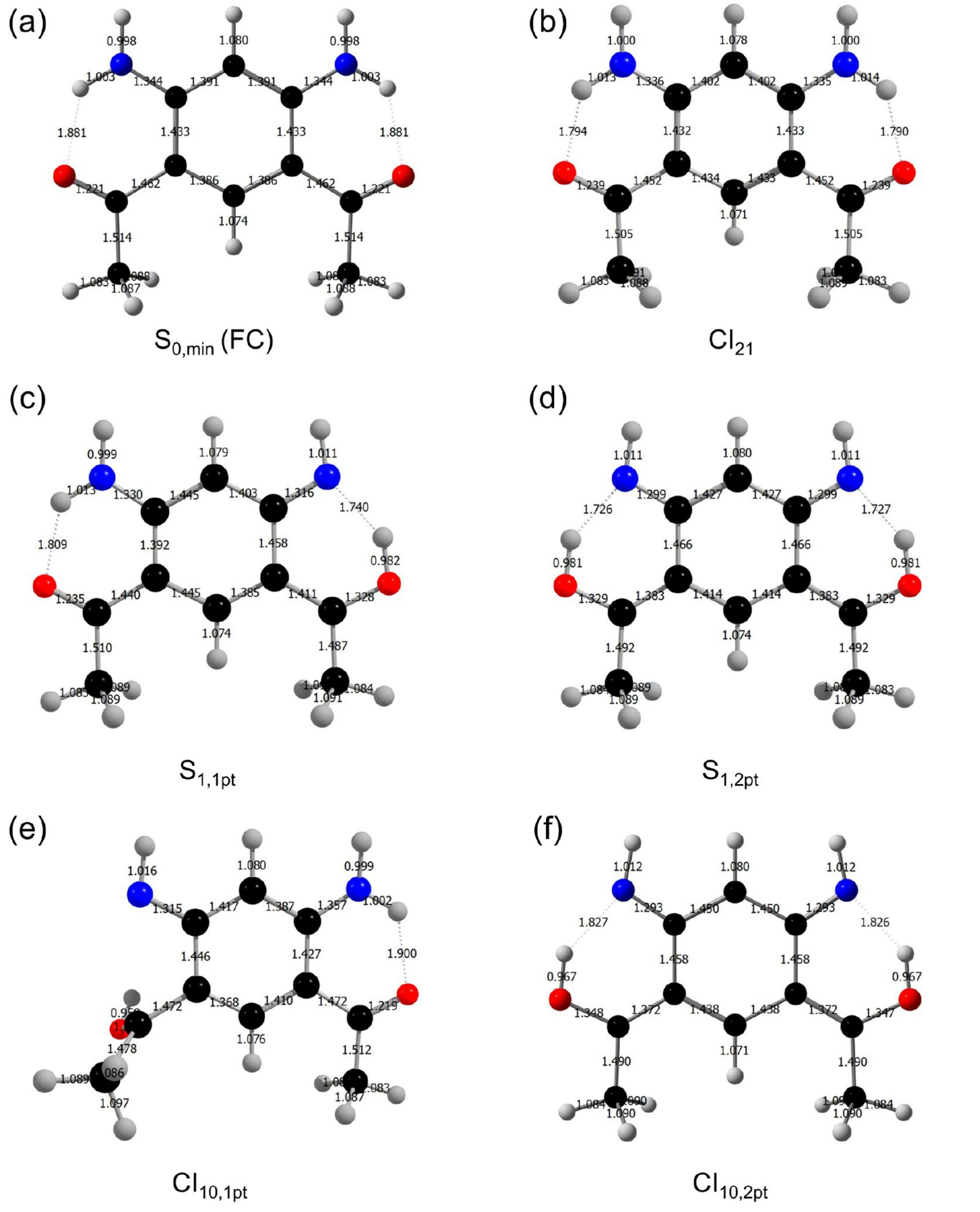

Supplementary Fig. 13. Optimized geometries of ***m*-DAPA** at (a) $S_{0,\ min}$ (FC), (b) CI_{21}, (c) $S_{1,\ 1pt}$, (d) $S_{1,\ 2pt}$, (e) $CI_{10,\ 1pt}$, and (f) $CI_{10,\ 2pt}$ with bond lengths in Å.

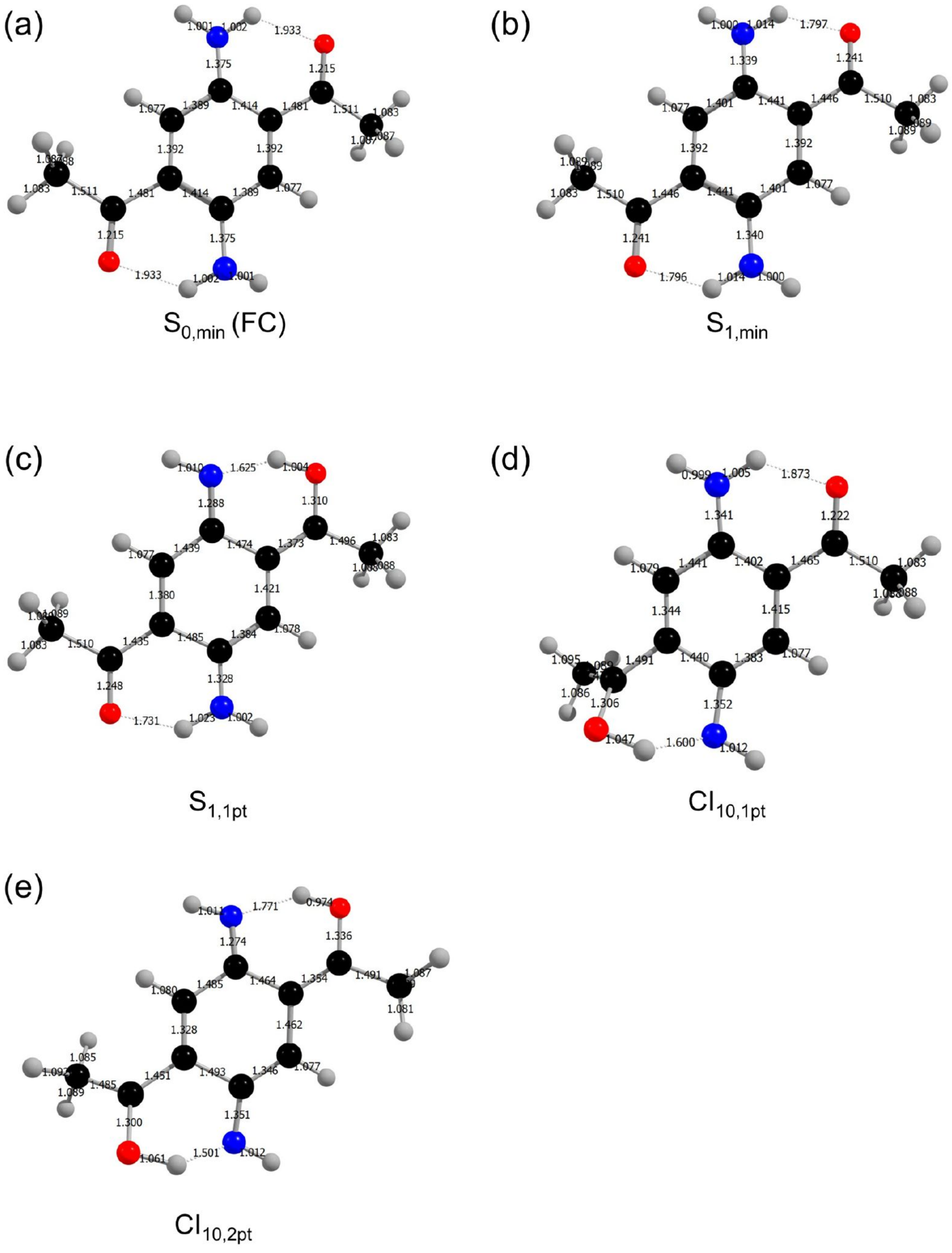

Supplementary Fig. 14. Optimized geometries of ***p*-DAPA** at (a) $S_{0,\ \mathrm{min}}$ (FC), (b) $S_{1,\ \mathrm{min}}$, (c) $S_{1,\ \mathrm{1pt}}$, (d) $CI_{10,\ \mathrm{1pt}}$, and (e) $CI_{10,\ \mathrm{2pt}}$ with bond lengths in Å.

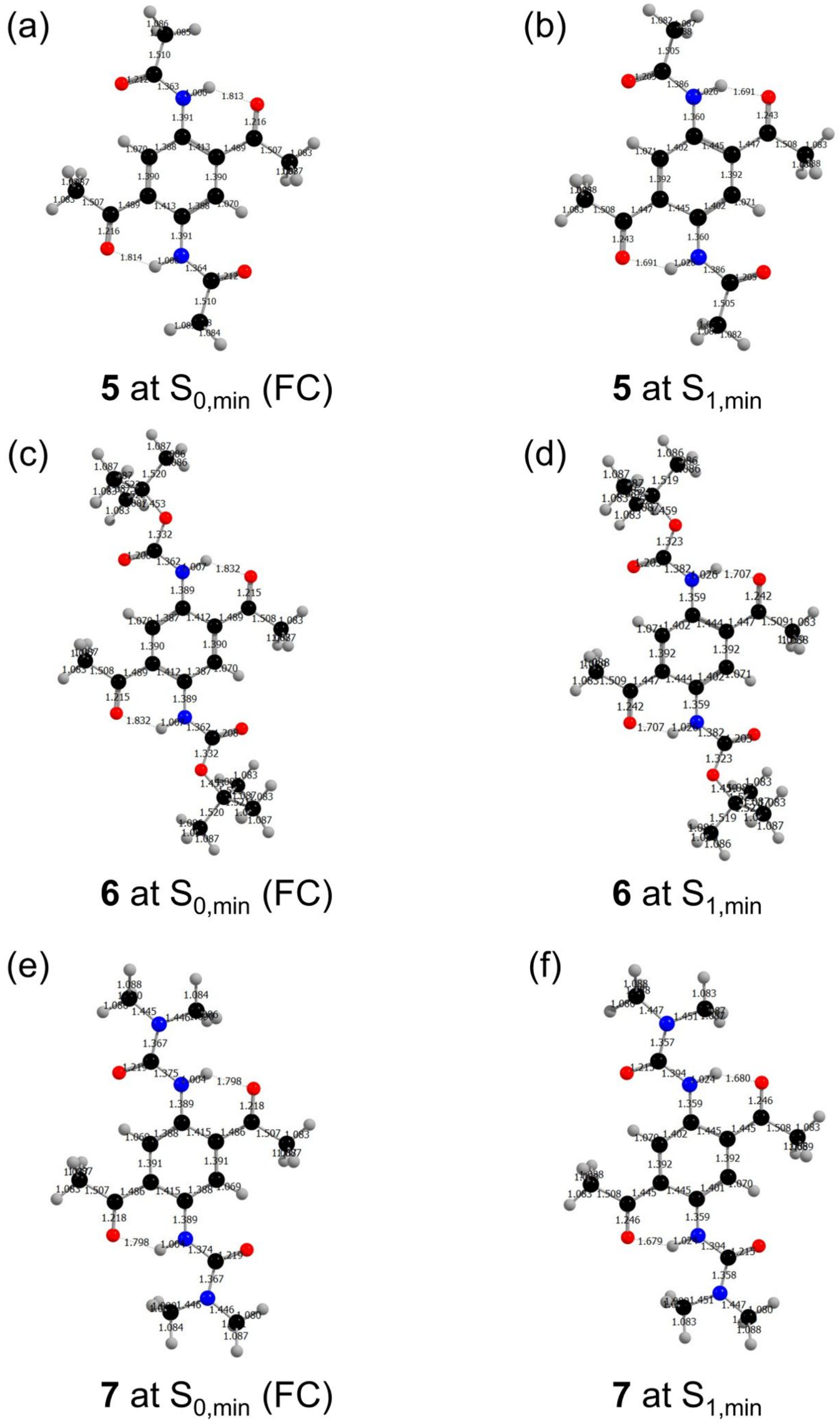

Supplementary Fig. 15. Optimized geometries of (a) **5** at $S_{0,\ min}$ (FC), (b) **5** at $S_{1,\ min}$, (c) **6** at $S_{0,\ min}$ (FC), (d) **6** at $S_{1,\ min}$, (e) **7** at $S_{0,\ min}$ (FC), and (f) **7** at $S_{1,\ min}$ with bond lengths in Å.

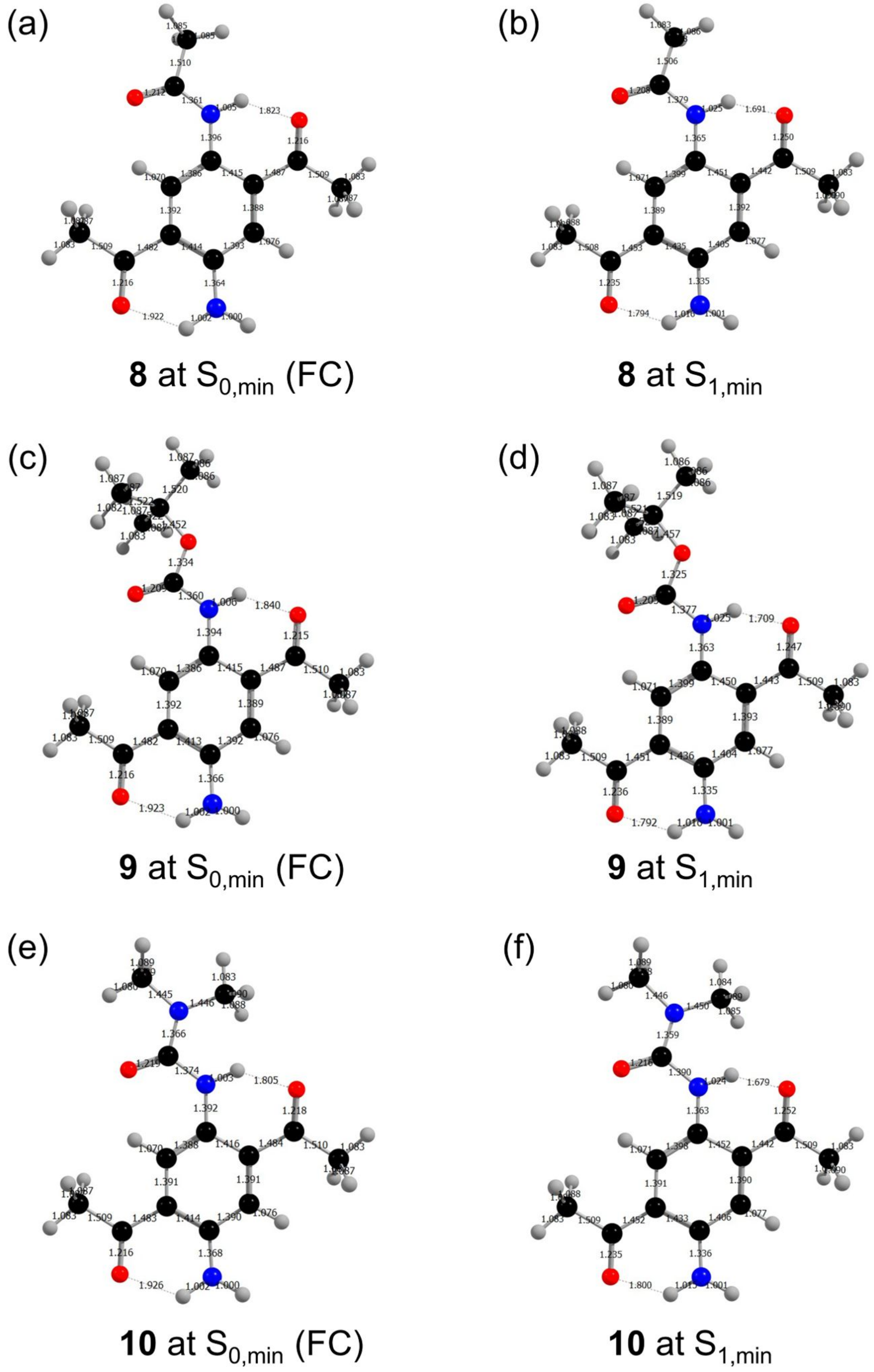

Supplementary Fig. 16. Optimized geometries of (a) **8** at $S_{0,\ \mathrm{min}}$ (FC), (b) **8** at $S_{1,\ \mathrm{min}}$, (c) **9** at $S_{0,\ \mathrm{min}}$ (FC), (d) **9** at $S_{1,\ \mathrm{min}}$, (e) **10** at $S_{0,\ \mathrm{min}}$ (FC), and (f) **10** at $S_{1,\ \mathrm{min}}$ with bond lengths in Å.

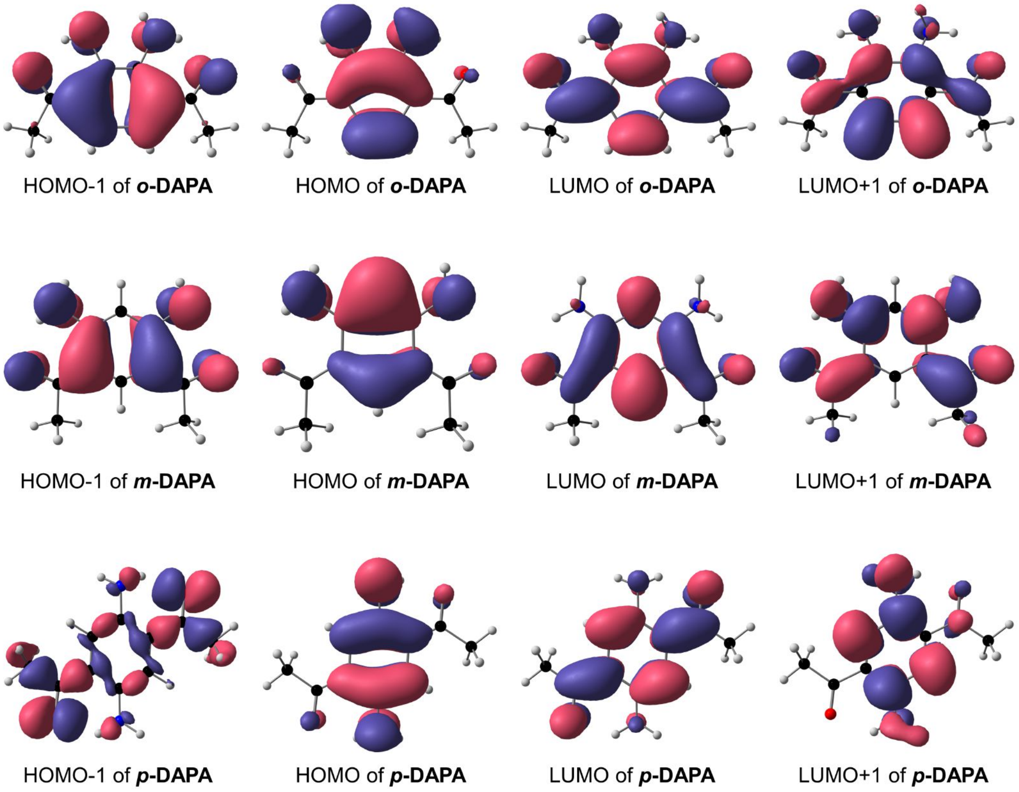

Supplementary Fig. 17. Plots of frontier molecular orbitals of ***o*-DAPA**, ***m*-DAPA**, and ***p*-DAPA**.

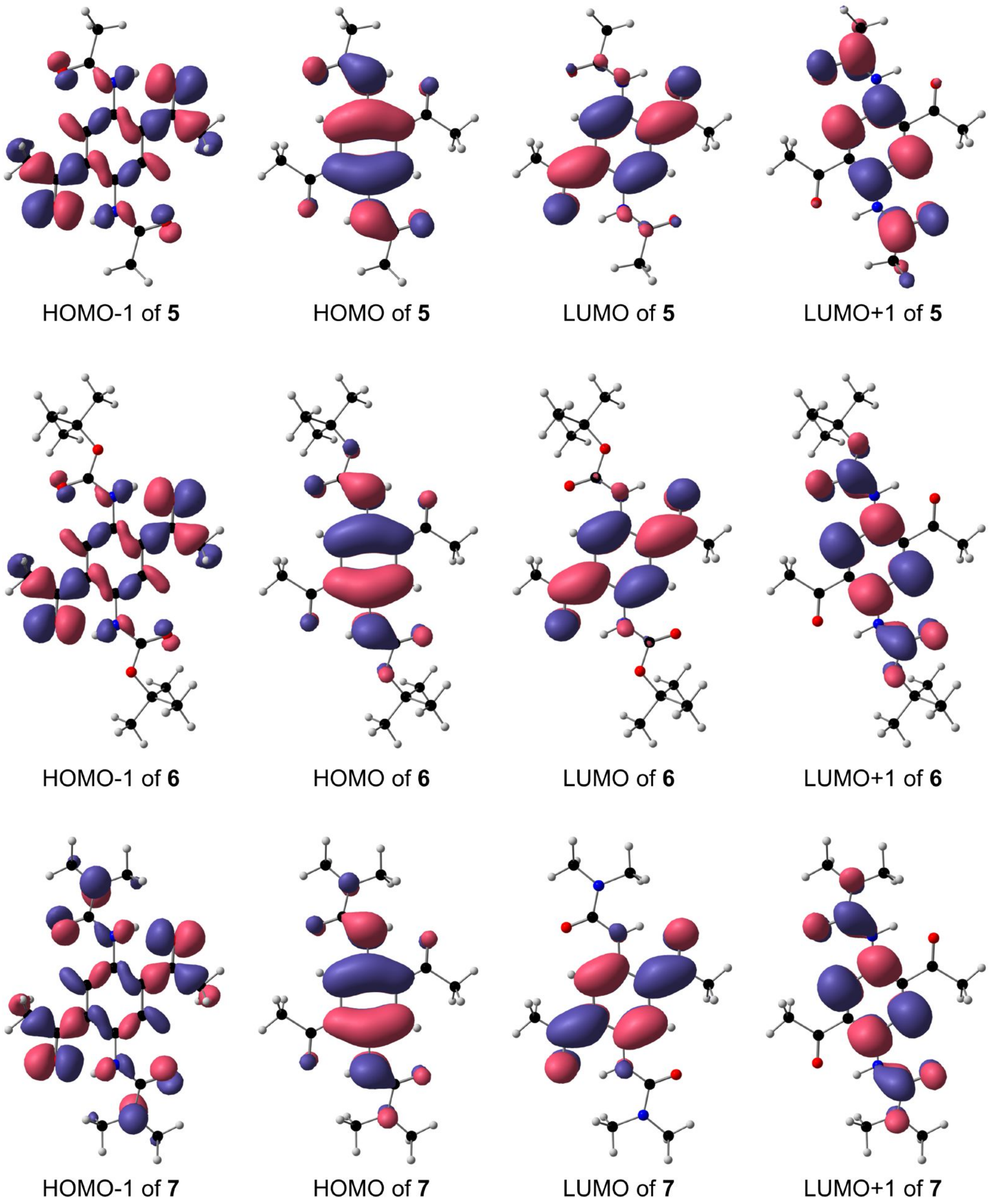

Supplementary Fig. 18. Plots of frontier molecular orbitals of **5**–**7**.

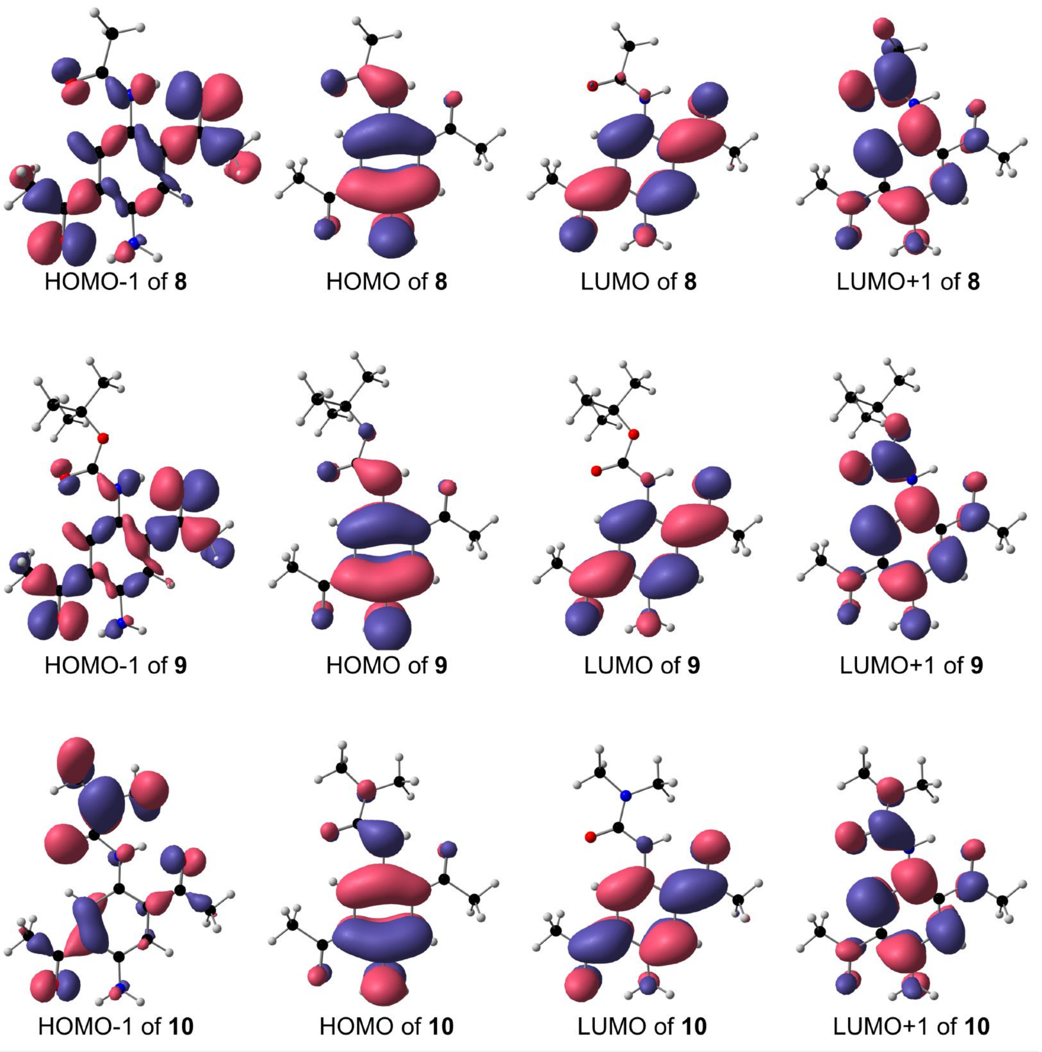

Supplementary Fig. 19. Plots of frontier molecular orbitals of **8**–**10**.

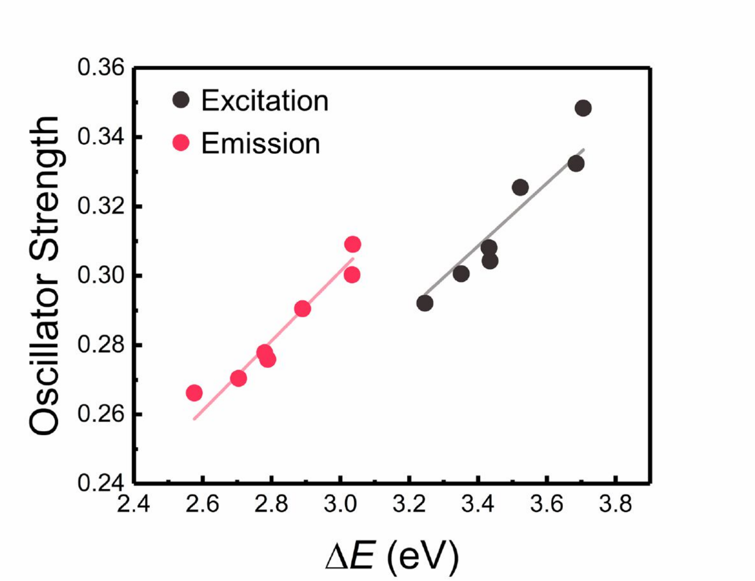

Supplementary Fig. 20. Plot of oscillator strength vs electronic excitation (black circles) and emission (red circles) energies of ***p*-DAPA** and **5–10**, calculated at the MRSF/BH&HLYP/6-31G* level of theory.

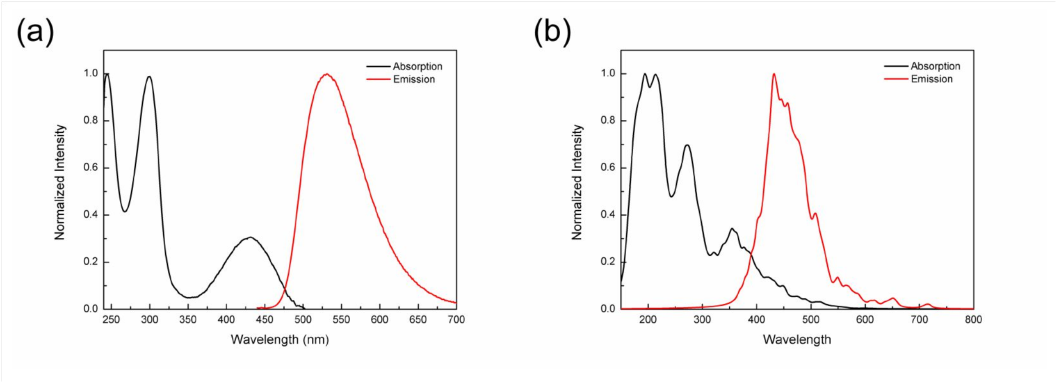

Supplementary Fig. 21. (a) Experimental and (b) calculated absorption (black) and emission (red) spectra of ***o*-DAPA** in $CHCl_3$. In (b), the absorption spectrum was constructed by using ten lowest singlet excited state (S_1 – S_{10}) sampled by Wigner distribution of S_0 equilibrium conformation at T = 300 K. The emission spectrum was constructed from the $S_{1,\ min}$ conformation.

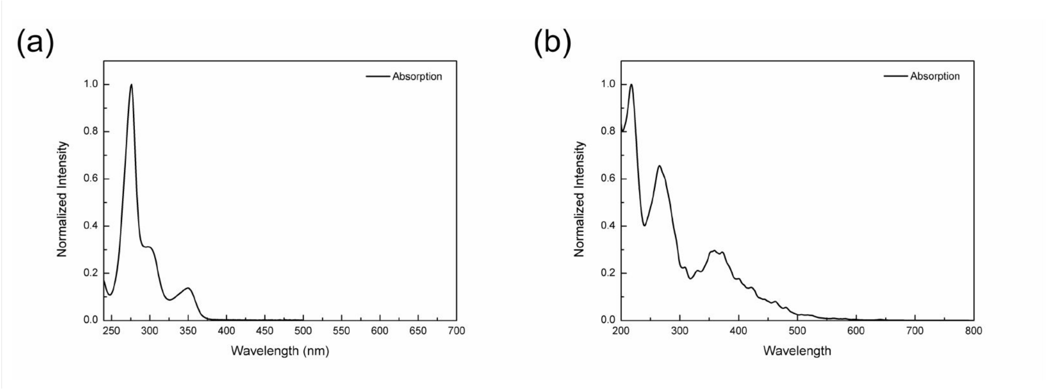

Supplementary Fig. 22. (a) Experimental and (b) calculated absorption spectra of ***m*-DAPA** in $CHCl_3$. In (b), the absorption spectrum was constructed by using ten lowest singlet excited state (S_1 – S_{10}) sampled by Wigner distribution of S_0 equilibrium conformation at T = 300 K.

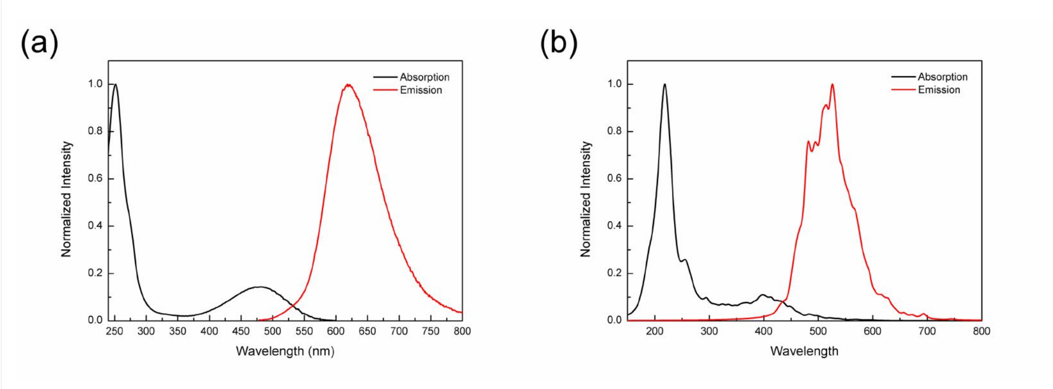

Supplementary Fig. 23. (a) Experimental and (b) calculated absorption (black) and emission (red) spectra of ***p*-DAPA** in $CHCl_3$. In (b), the absorption spectrum was constructed by using ten lowest singlet excited state (S_1 – S_{10}) sampled by Wigner distribution of S_0 equilibrium conformation at T = 300 K. The emission spectrum was constructed from the $S_{1,\text{ min}}$ conformation.

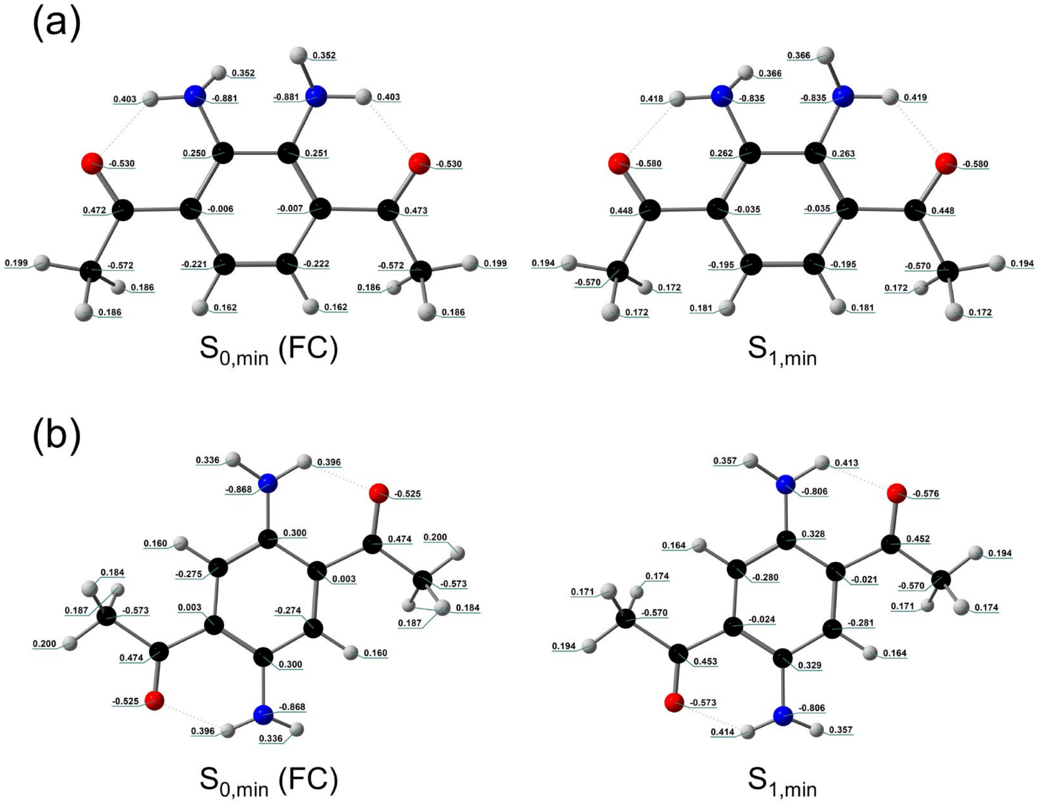

Supplementary Fig. 24. Mulliken charge analysis of (a) ***o*-DAPA** and (b) ***p*-DAPA** in optimized geometries at S_0 and S_1.

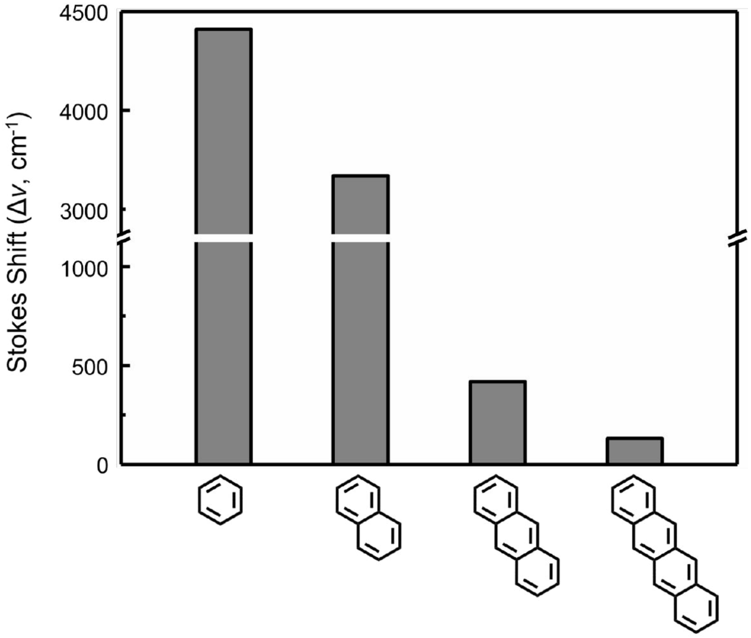

Supplementary Fig. 25. Stokes shifts of benzene, naphthalene, anthracene, and tetracene[21].

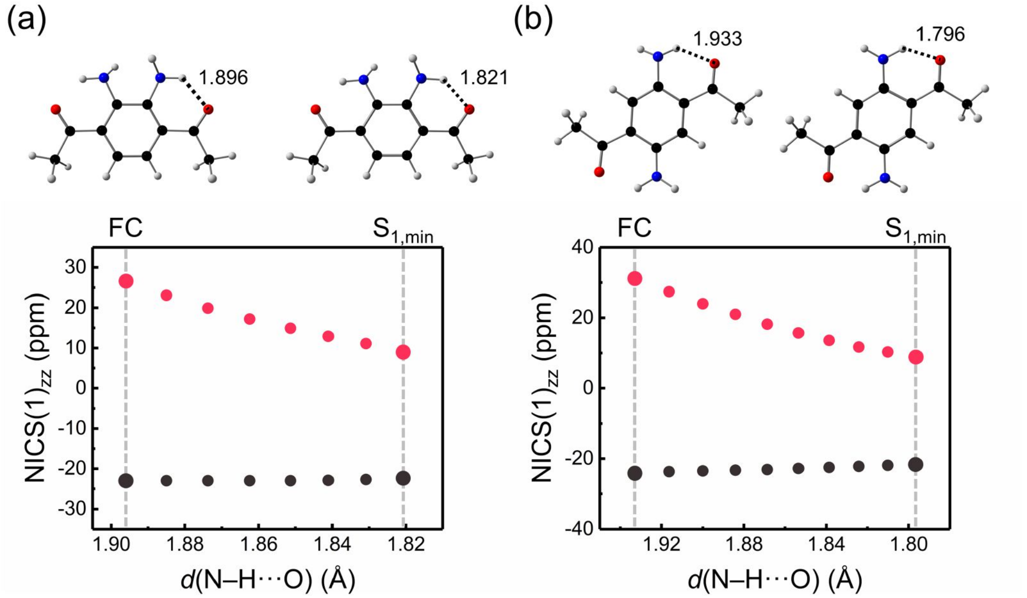

Supplementary Fig. 26. Relationship between the hydrogen bond distance $d_{\mathrm{N–H}\cdots\mathrm{O}}$ and NICS(1)$_{\mathrm{zz}}$ value of (a) ***o*-DAPA** and (b) ***p*-DAPA** in the S_1 (red circles) and S_0 (black circles) states. Each point between S_1 FC and $S_{1,\mathrm{min}}$ was taken from the potential energy surfaces in Figure 6.

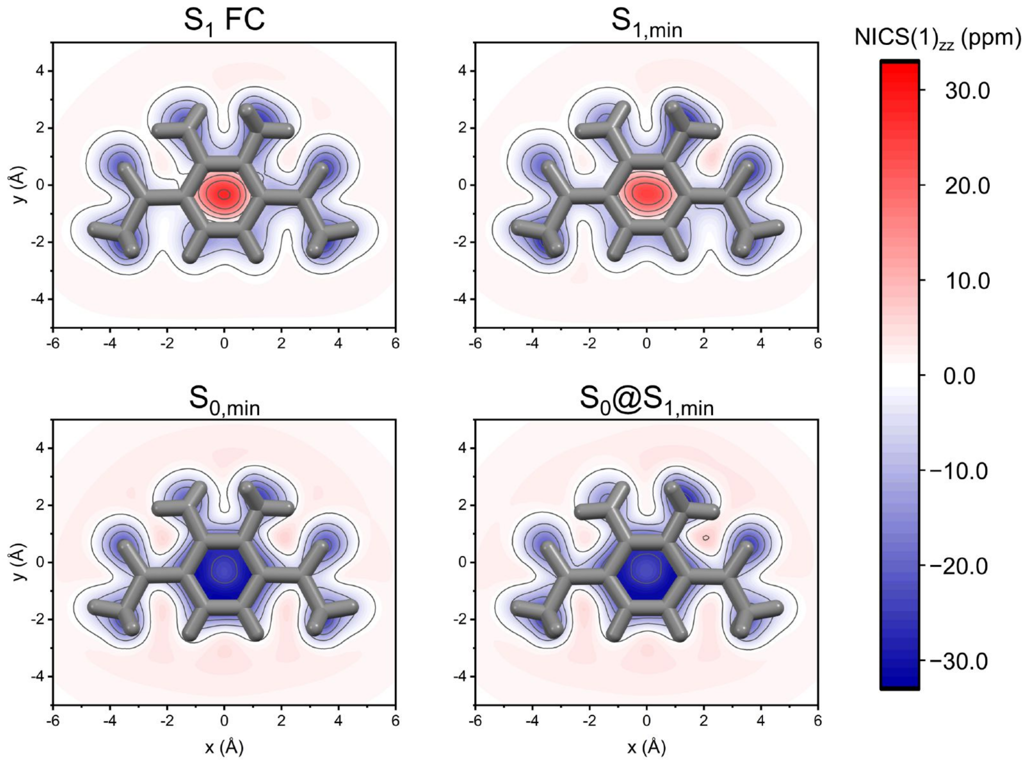

Supplementary Fig. 27. NICS(1)$_{zz}$ grids in the molecular plane of ***o*-DAPA**.

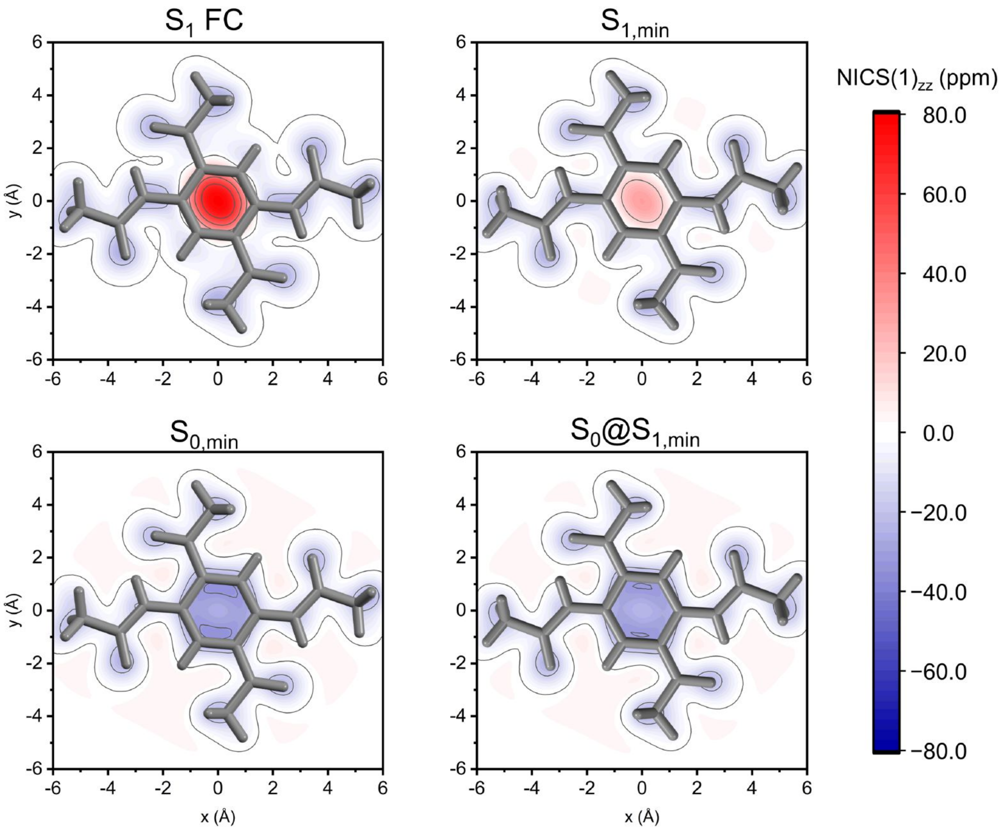

Supplementary Fig. 28. NICS(1)$_{zz}$ grids in the molecular plane of **5**.

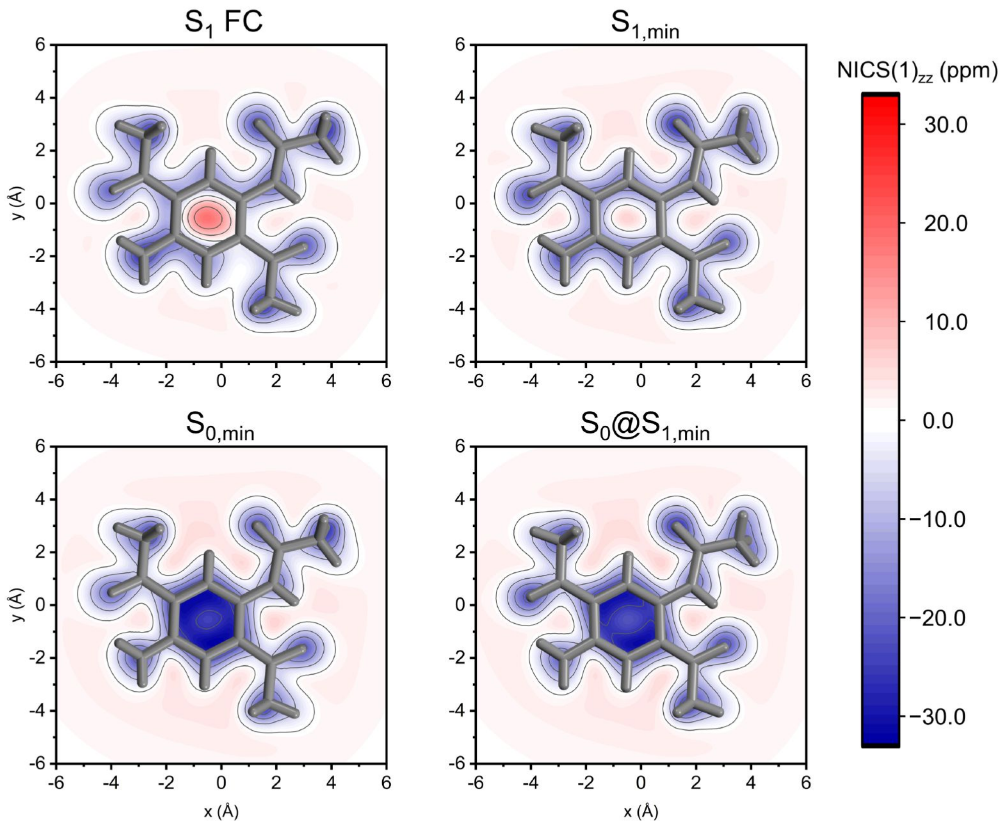

Supplementary Fig. 29. NICS$(1)_{zz}$ grids in the molecular plane of **8**.

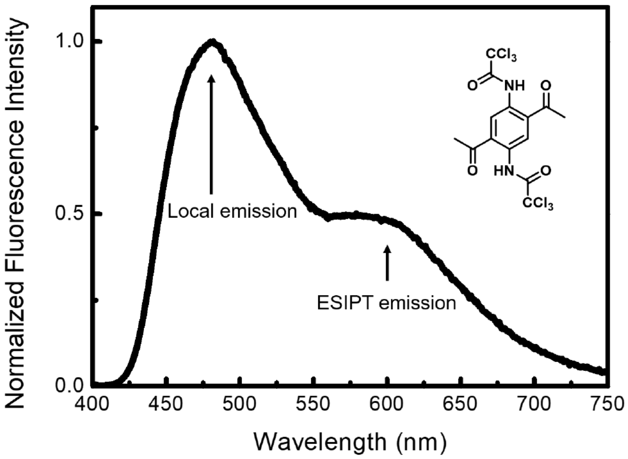

Supplementary Fig. 30. Normalized emission spectra of **11** in $CHCl_3$ at T = 298 K.

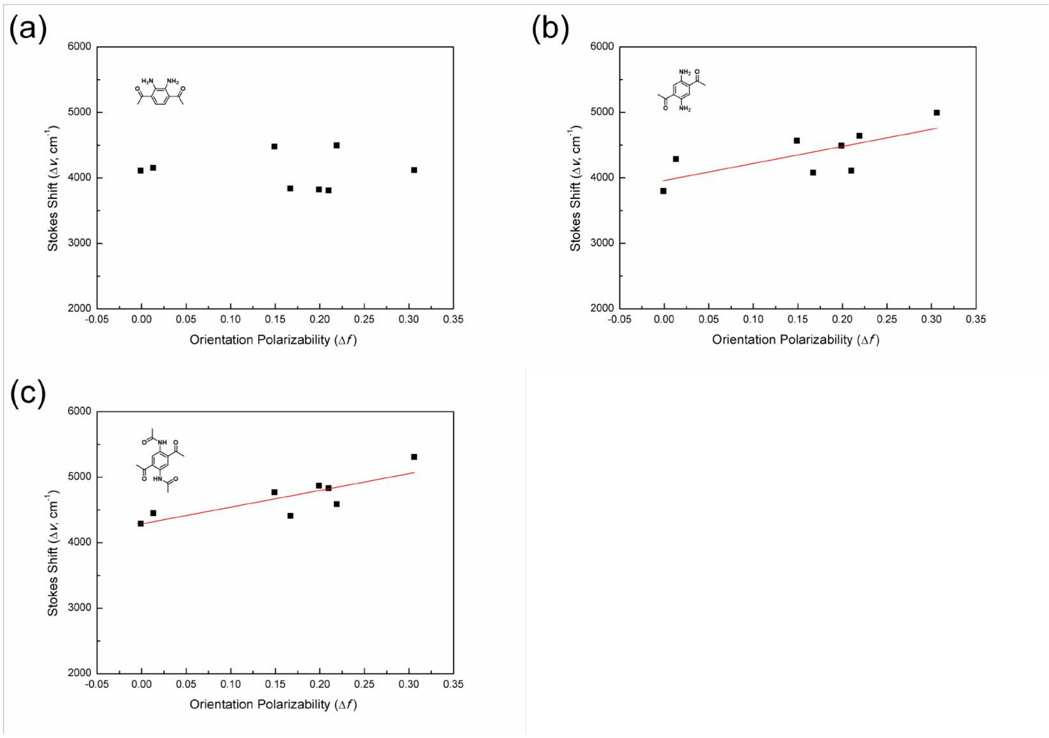

Supplementary Fig. 31. Lippert–Mataga plots for (a) ***o*-DAPA**, (b) ***p*-DAPA**, and (c) **5**. See page S-8 for details.

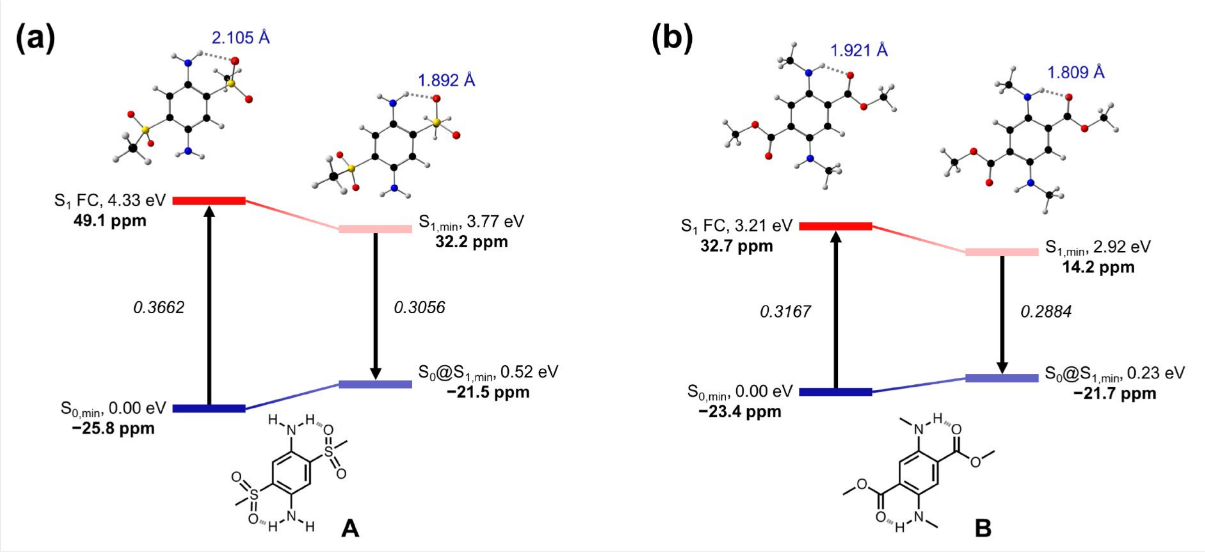

Supplementary Fig. 32. Schematic energy diagram and calculated NICS(1)$_{zz}$ values (in bold) at the optimized geometries for reported single-benzene fluorophores (a) **A**[22], and (b) **B**[23]. For each geometries, the averaged N–H$\cdots$O hydrogen bond distances are shown in blue; the oscillator strengths in italic.

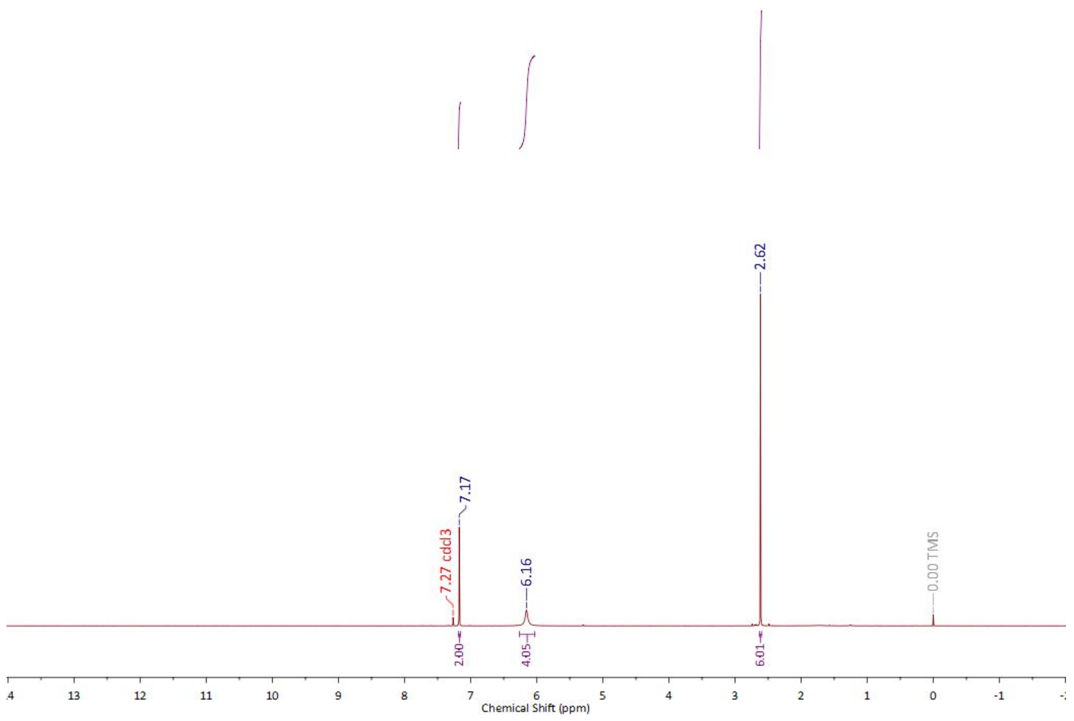

Supplementary Fig. 33. ^{1}H NMR (500 MHz) spectrum of ***o*-DAPA** in $CDCl_3$ (T = 298 K).

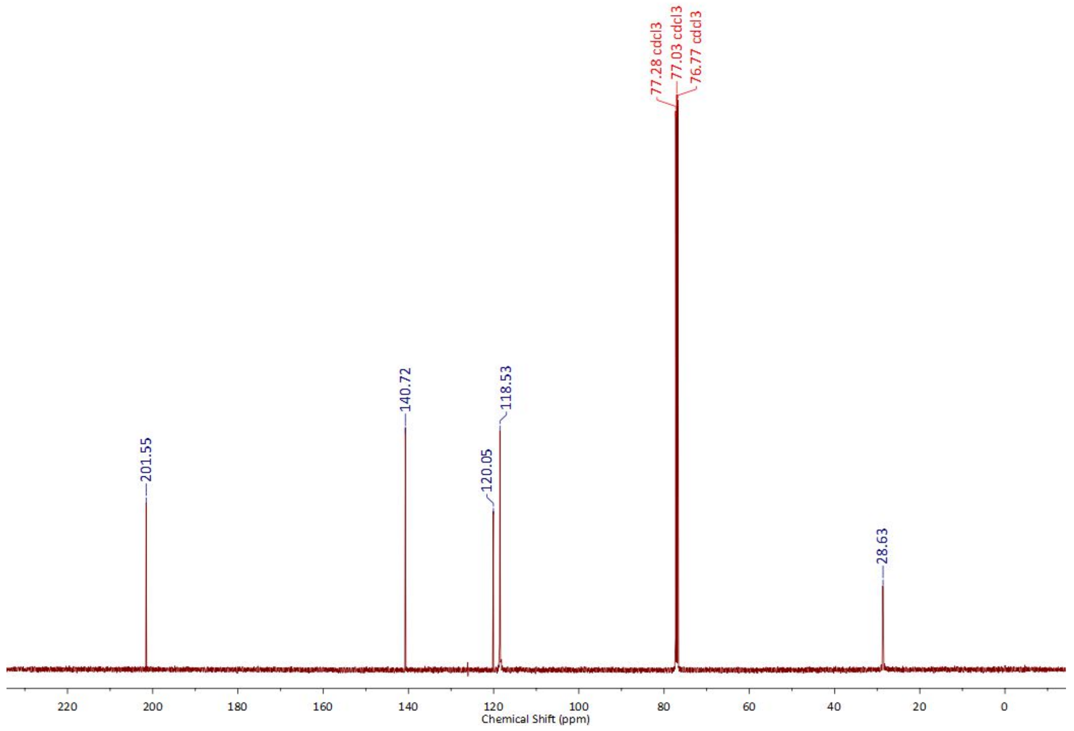

Supplementary Fig. 34. ^{13}C NMR (125 MHz) spectrum of ***o*-DAPA** in $CDCl_3$ (T = 298 K).

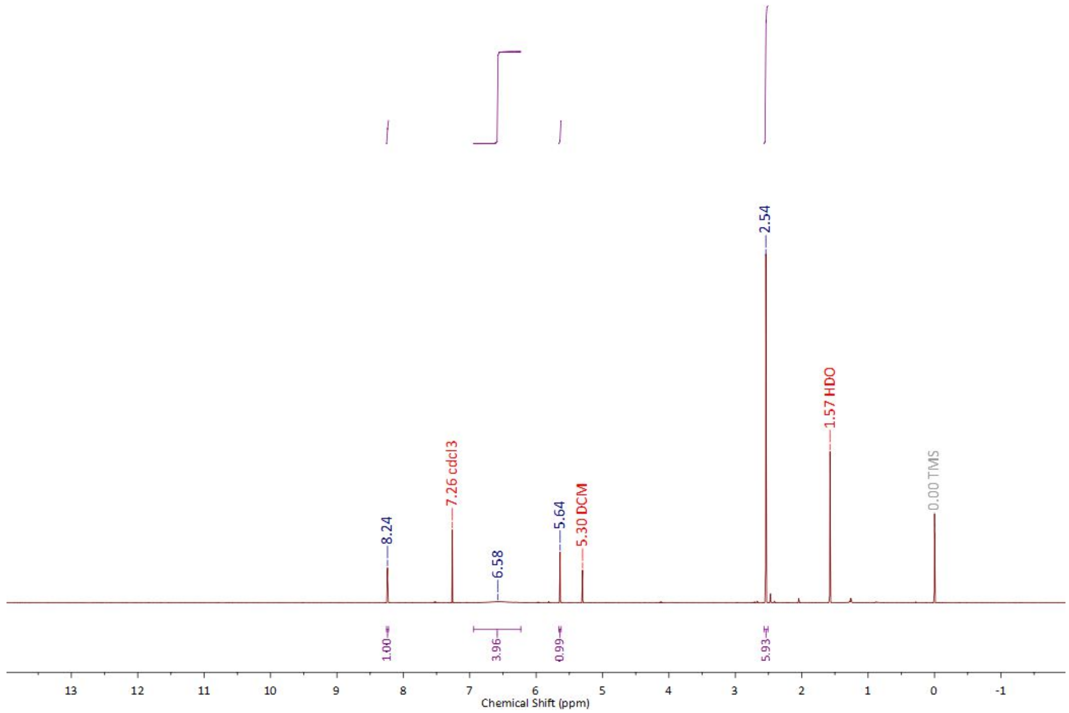

Supplementary Fig. 35. ^{1}H NMR (500 MHz) spectrum of ***m*-DAPA** in $CDCl_3$ (T = 298 K).

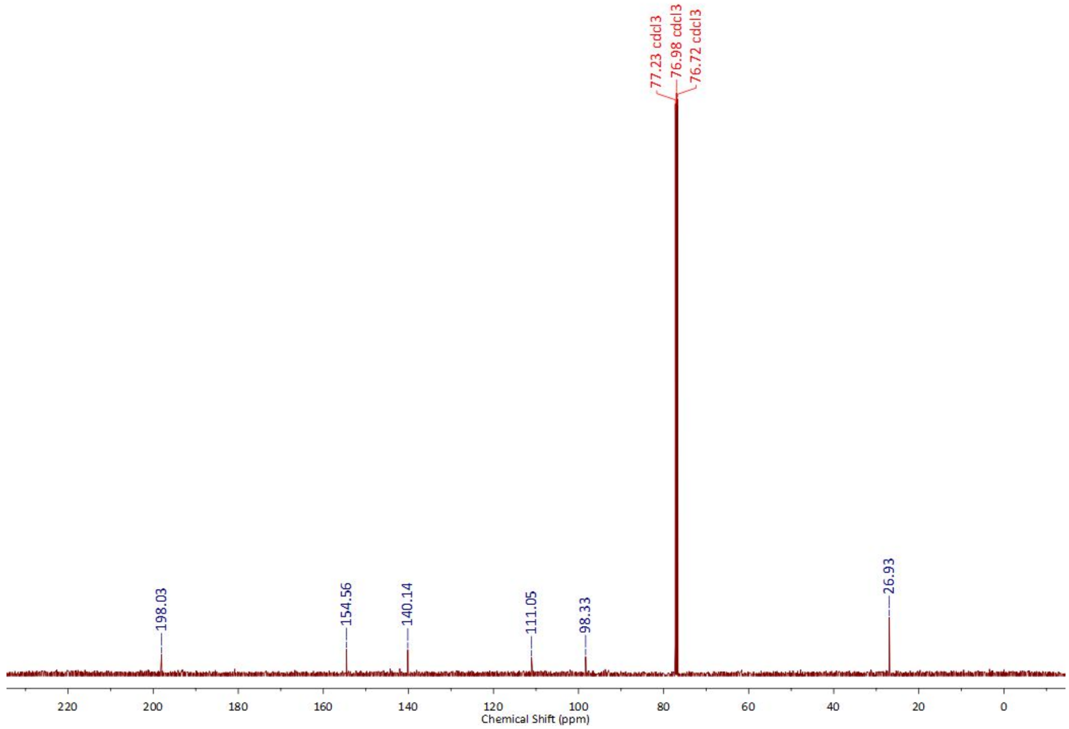

Supplementary Fig. 36. ^{13}C NMR (125 MHz) spectrum of ***m*-DAPA** in $CDCl_3$ (T = 298 K).

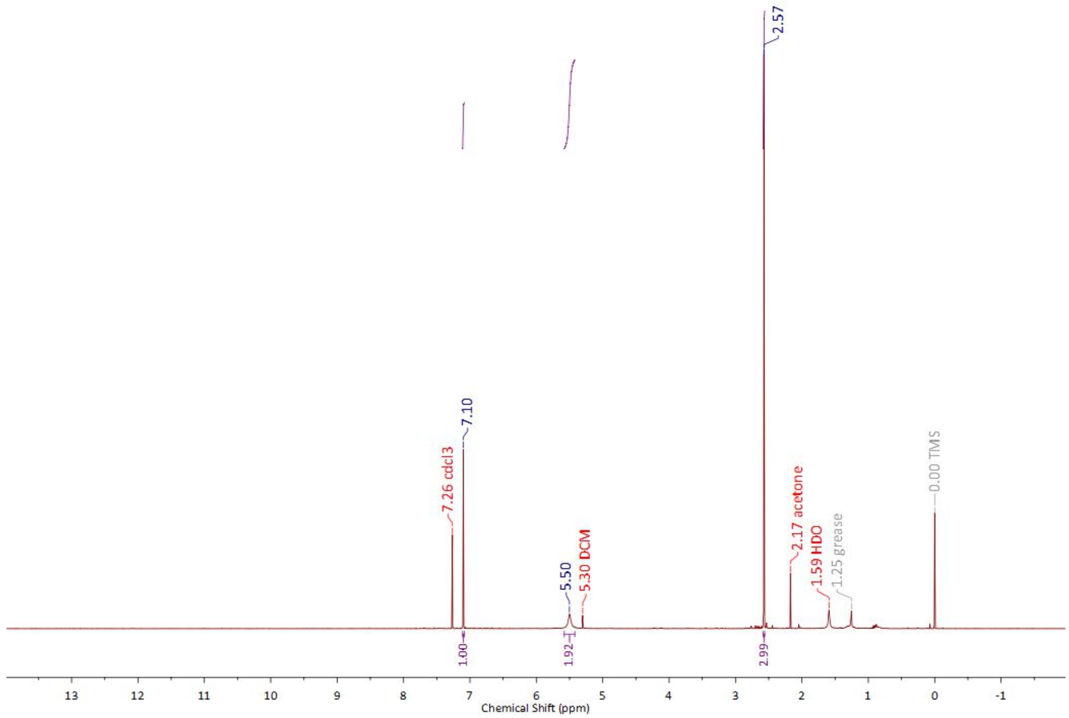

Supplementary Fig. 37. ^{1}H NMR (500 MHz) spectrum of ***p*-DAPA** in $CDCl_3$ (T = 298 K).

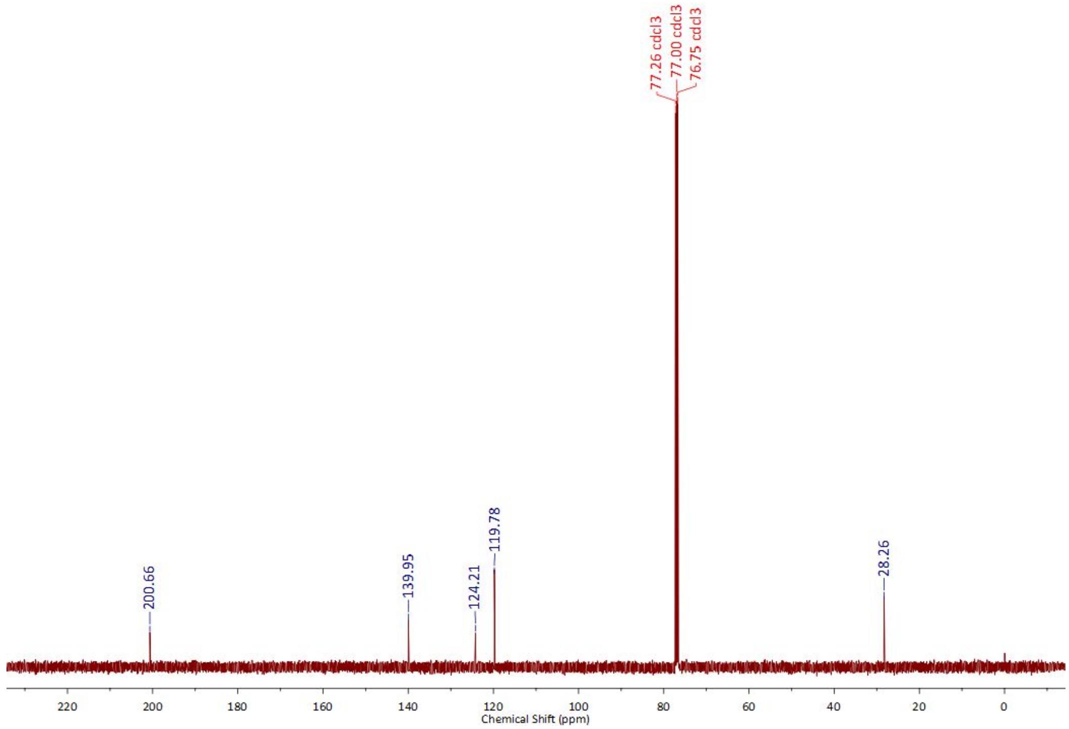

Supplementary Fig. 38. ^{13}C NMR (125 MHz) spectrum of ***p*-DAPA** in $CDCl_3$ (T = 298 K).

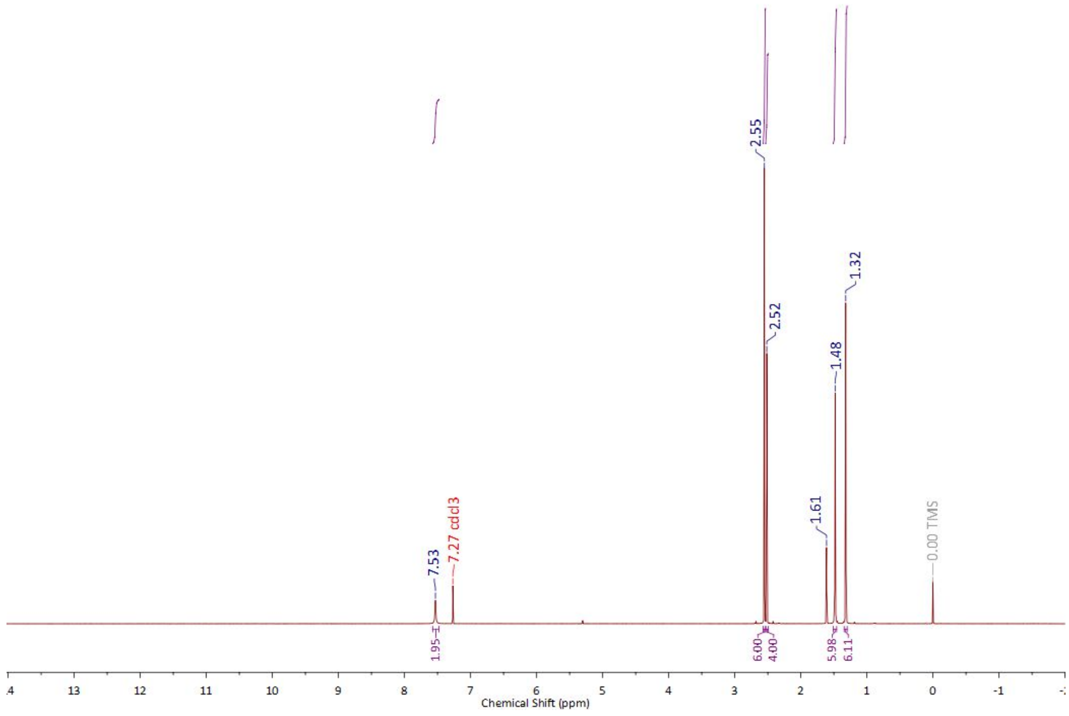

Supplementary Fig. 39. ^{1}H NMR (500 MHz) spectrum of **4** in $CDCl_3$ (T = 298 K).

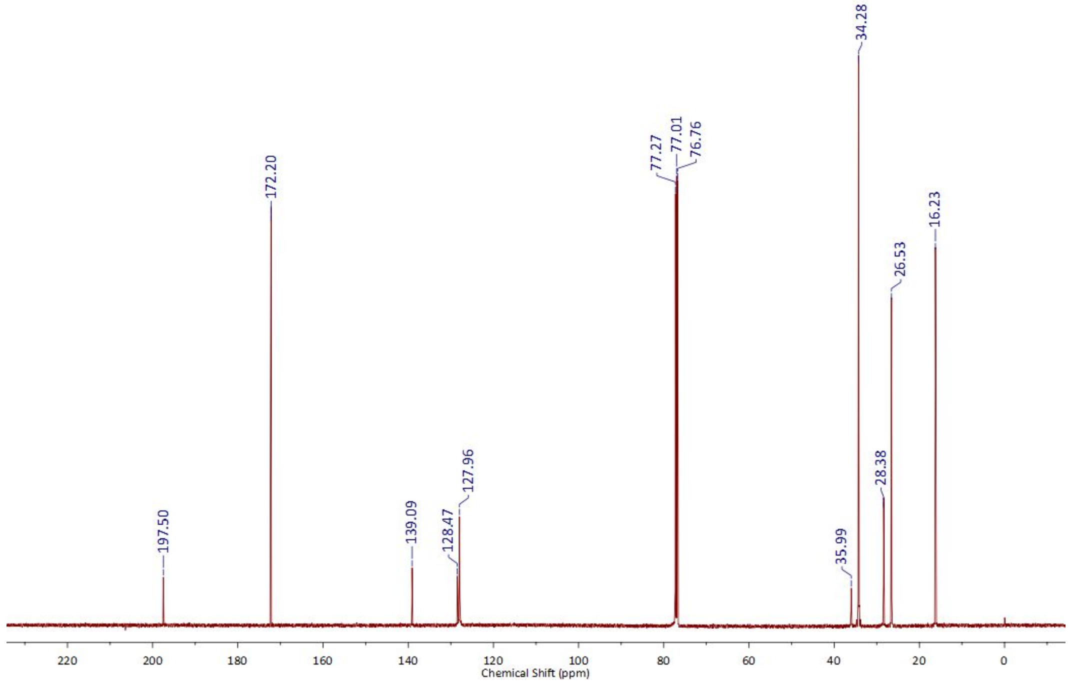

Supplementary Fig. 40. ^{13}C NMR (125 MHz) spectrum of **4** in $CDCl_3$ (T = 298 K).

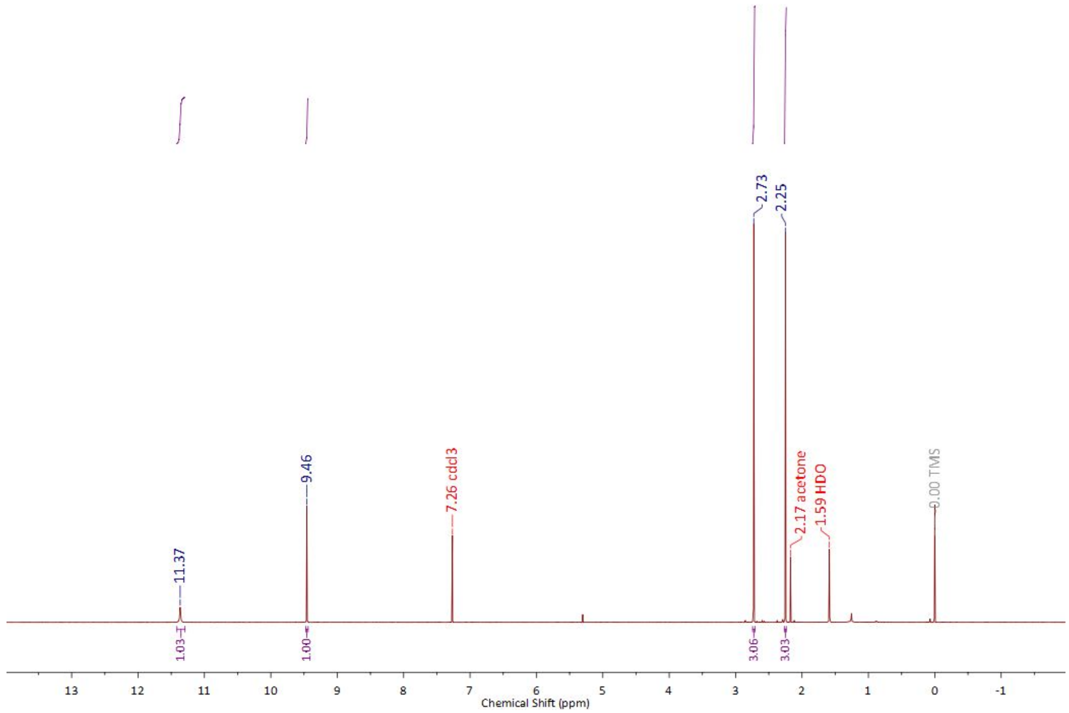

Supplementary Fig. 41. ^{1}H NMR (500 MHz) spectrum of **5** in $CDCl_3$ (T = 298 K).

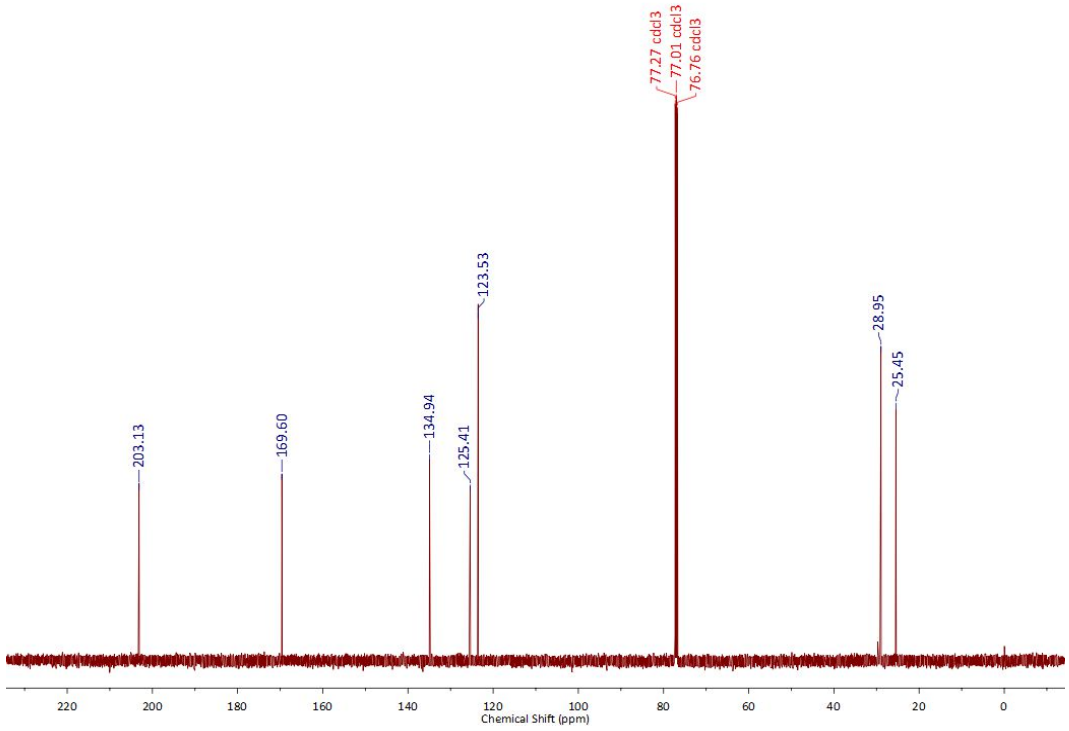

Supplementary Fig. 42. ^{13}C NMR (125 MHz) spectrum of **5** in $CDCl_3$ (T = 298 K).

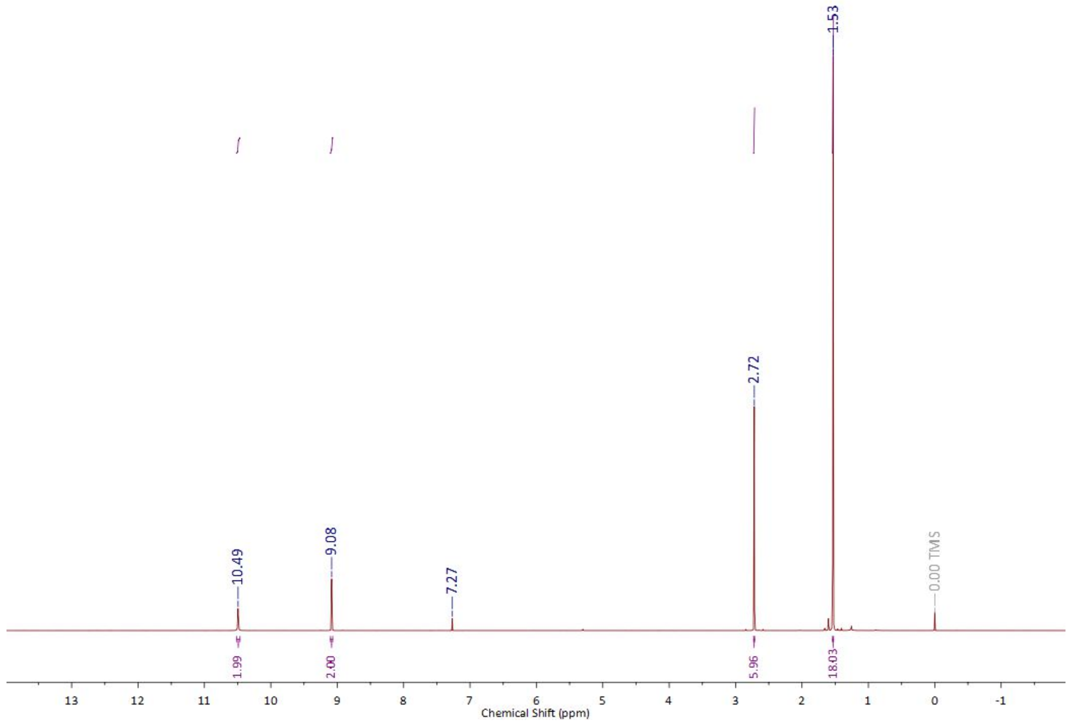

Supplementary Fig. 43. ^{1}H NMR (500 MHz) spectrum of **6** in $CDCl_3$ (T = 298 K).

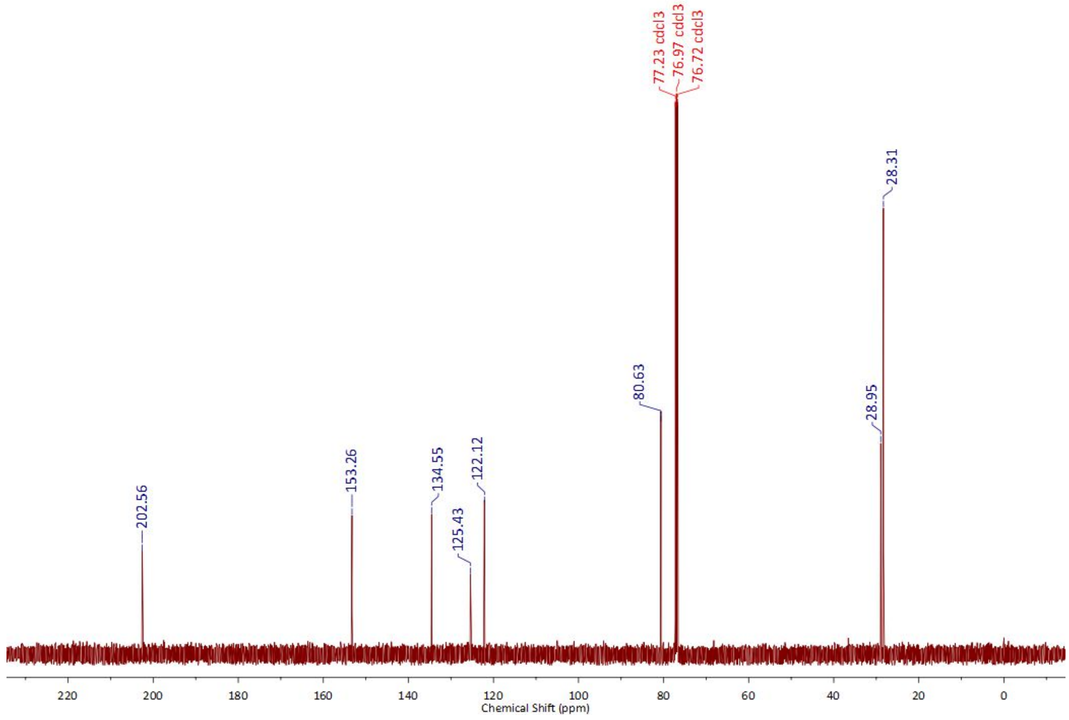

Supplementary Fig. 44. ^{13}C NMR (125 MHz) spectrum of **6** in $CDCl_3$ (T = 298 K).

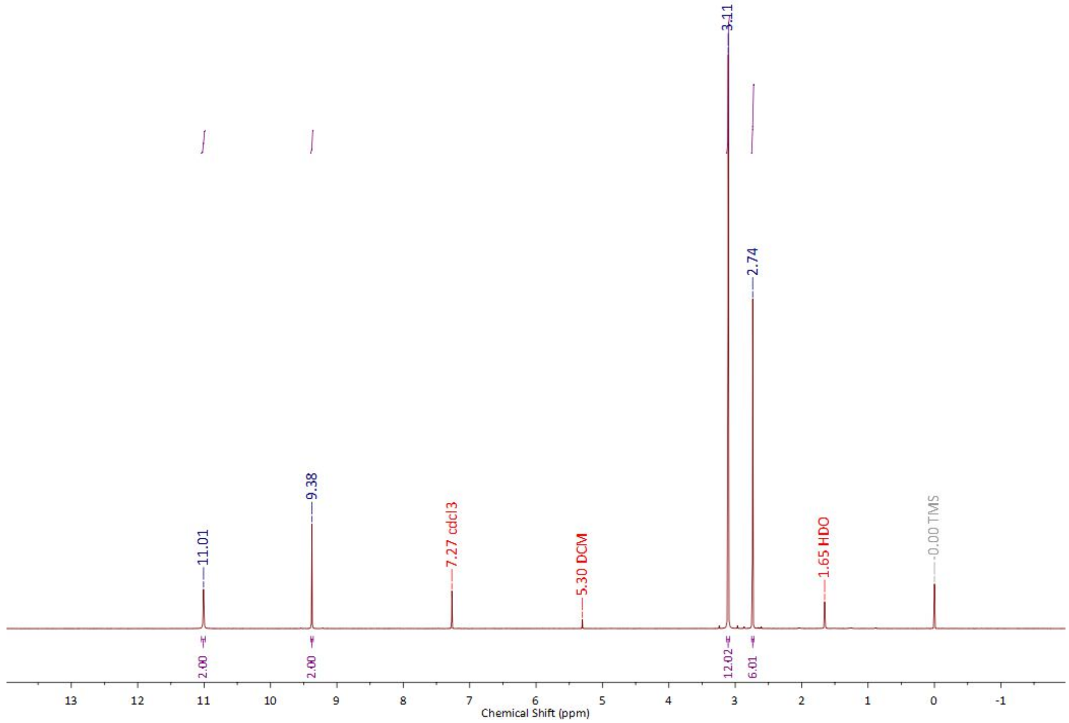

Supplementary Fig. 45. ^{1}H NMR (500 MHz) spectrum of **7** in $CDCl_3$ (T = 298 K).

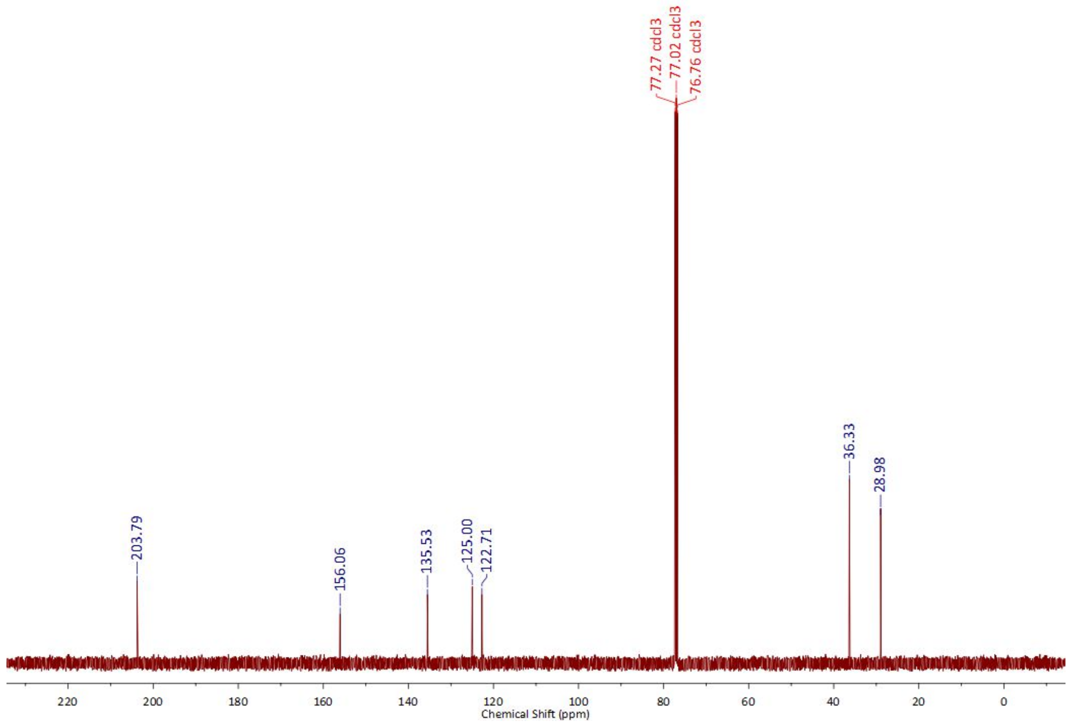

Supplementary Fig. 46. ^{13}C NMR (125 MHz) spectrum of **7** in $CDCl_3$ (T = 298 K).

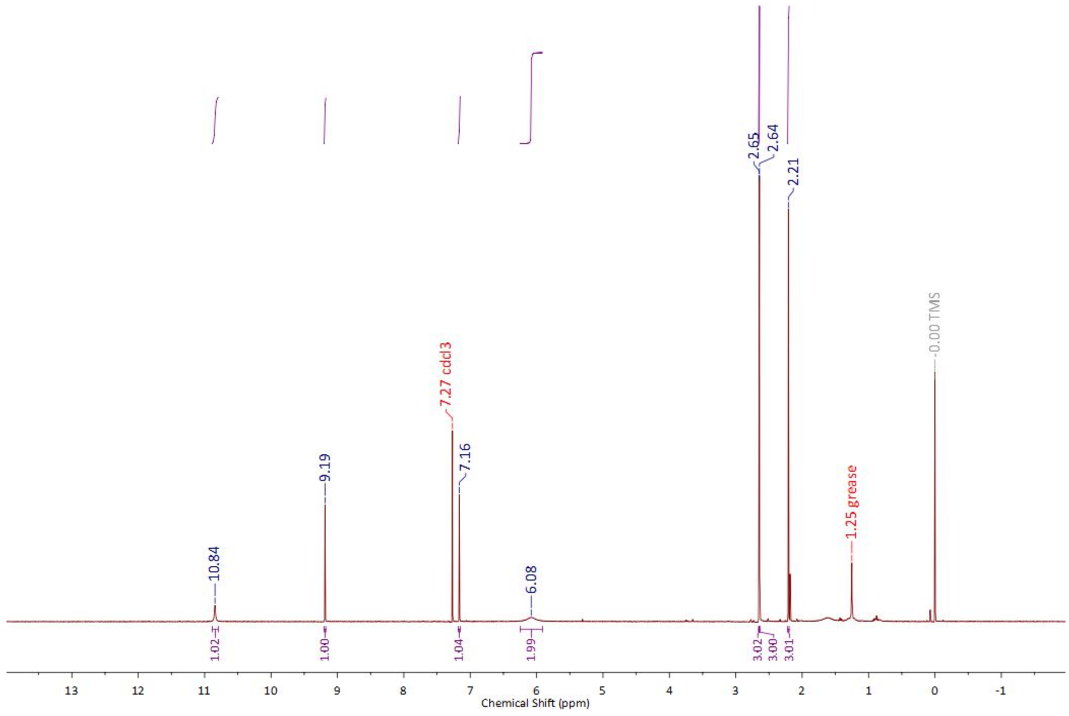

Supplementary Fig. 47. ^{1}H NMR (500 MHz) spectrum of **8** in $CDCl_3$ (T = 298 K).

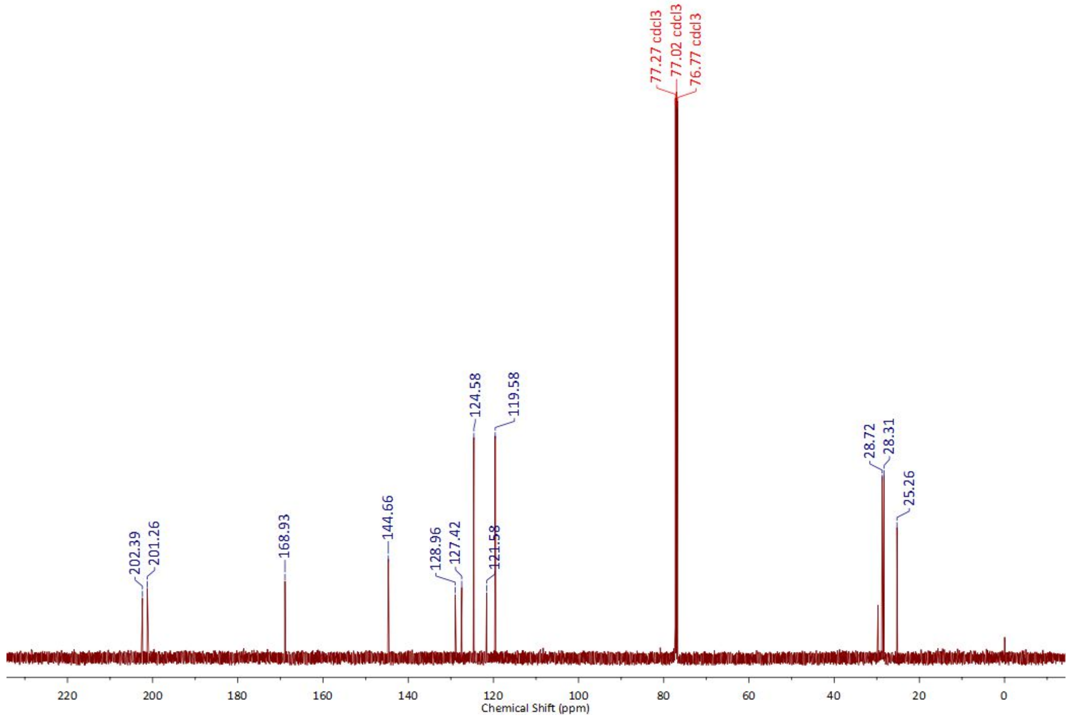

Supplementary Fig. 48. ^{13}C NMR (125 MHz) spectrum of **8** in $CDCl_3$ (T = 298 K).

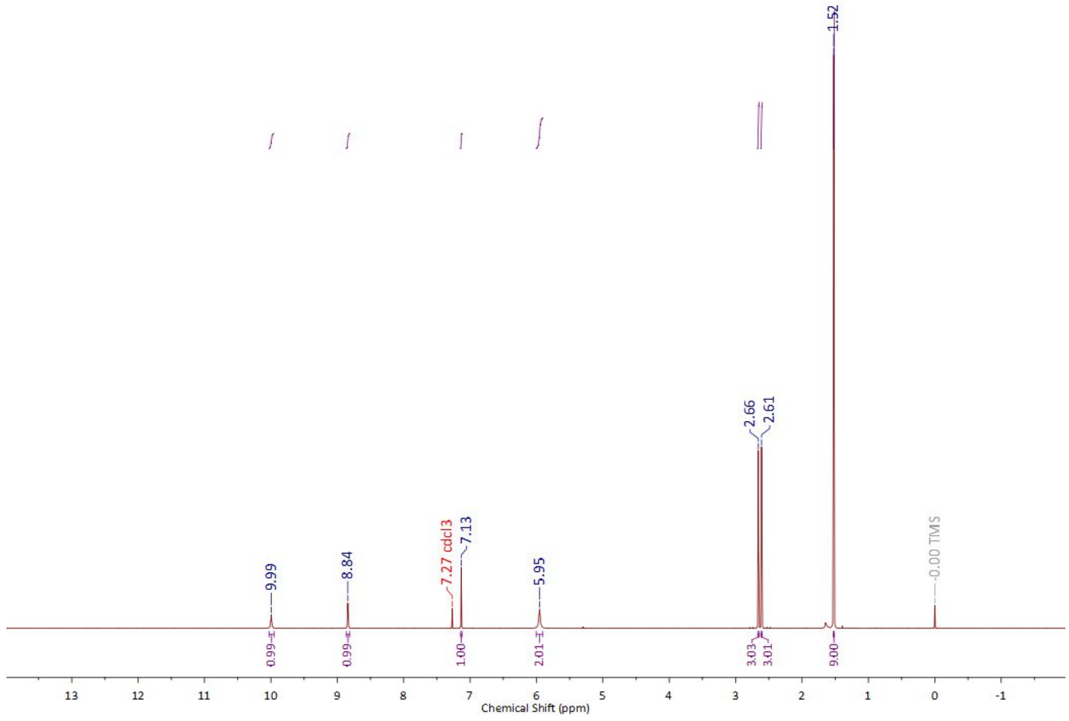

Supplementary Fig. 49. ^{1}H NMR (500 MHz) spectrum of **9** in $CDCl_3$ (T = 298 K).

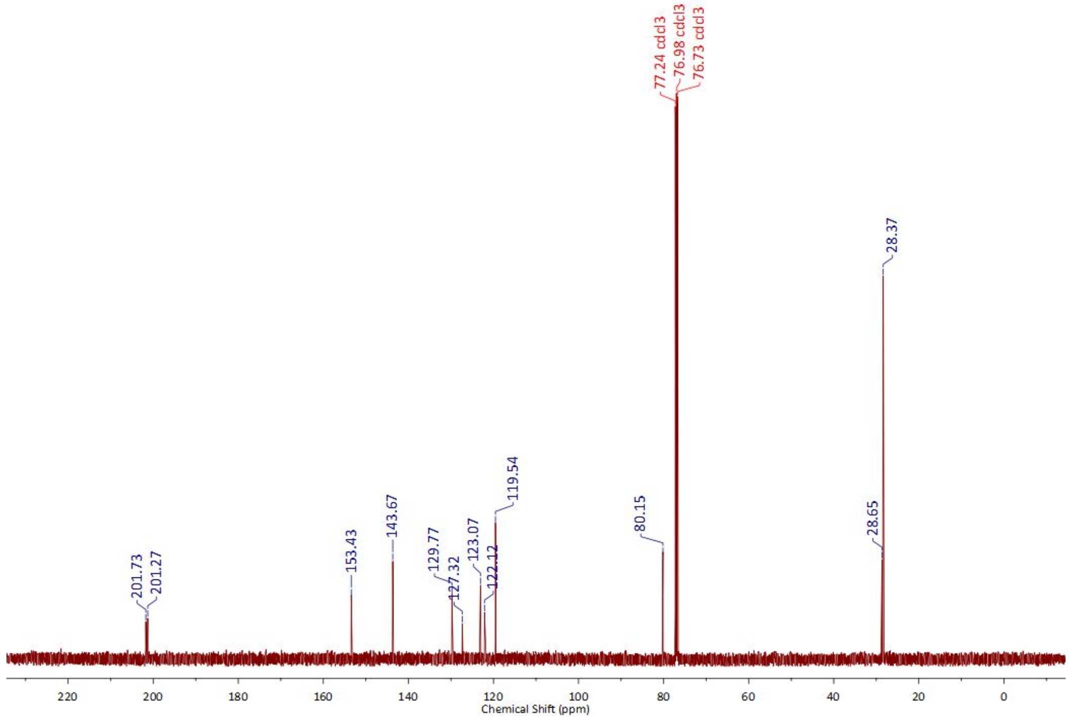

Supplementary Fig. 50. ^{13}C NMR (125 MHz) spectrum of **9** in $CDCl_3$ (T = 298 K).

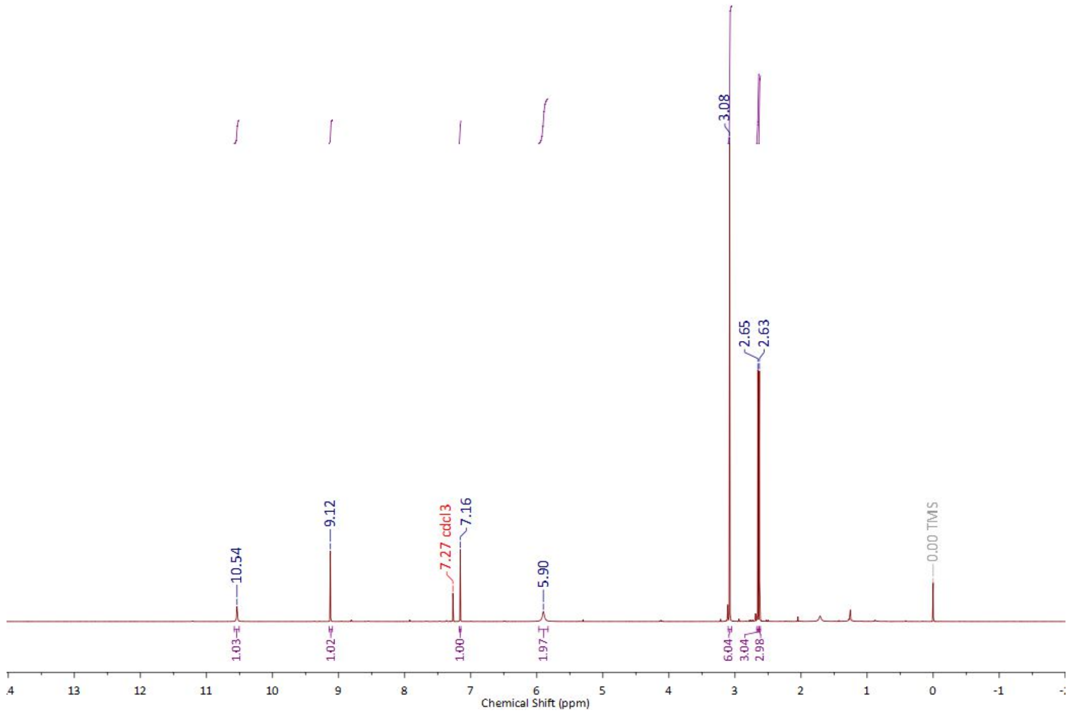

Supplementary Fig. 51. ^{1}H NMR (500 MHz) spectrum of **10** in $CDCl_3$ (T = 298 K).

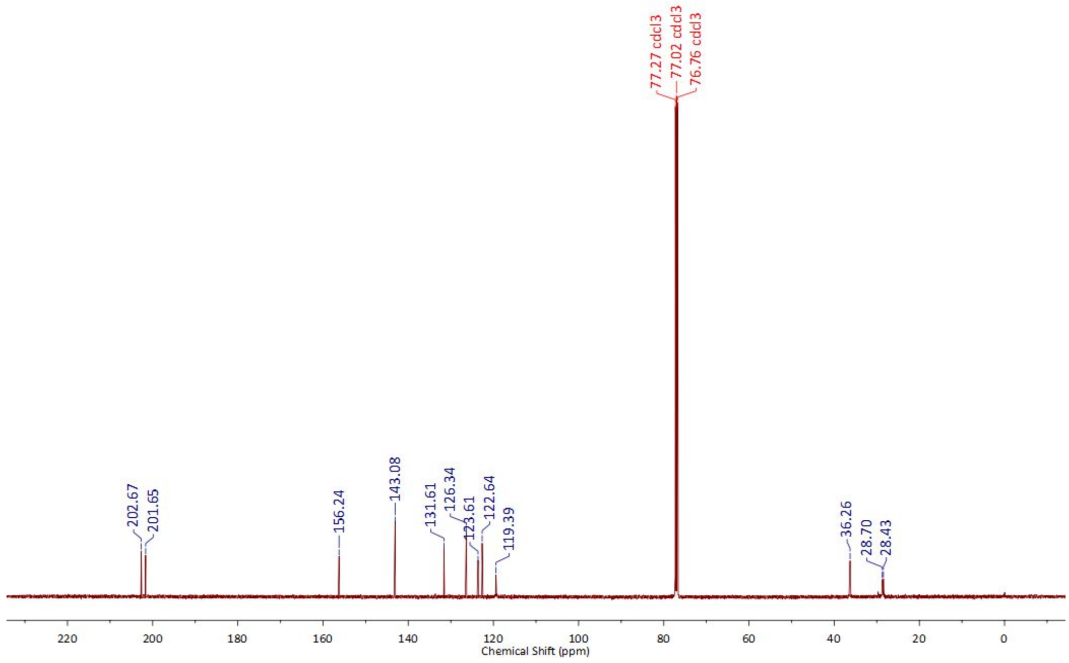

Supplementary Fig. 52. ^{13}C NMR (125 MHz) spectrum of **10** in $CDCl_3$ (T = 298 K).

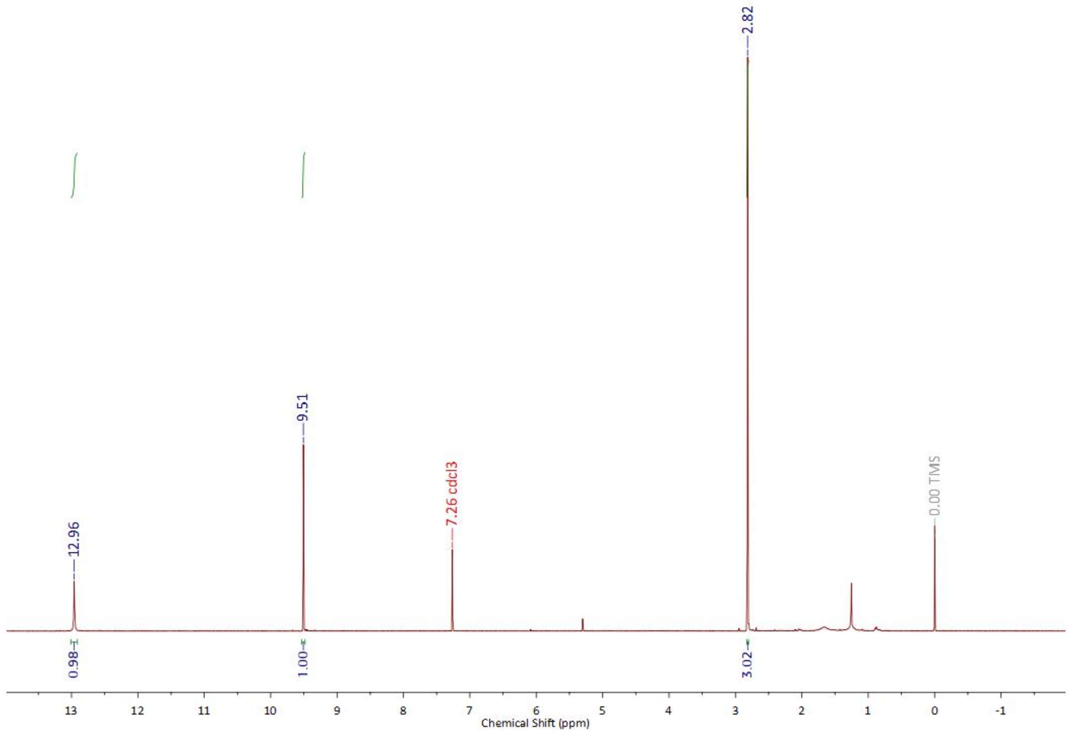

Supplementary Fig. 53. ^{1}H NMR (500 MHz) spectrum of **11** in $CDCl_3$ (T = 298 K).

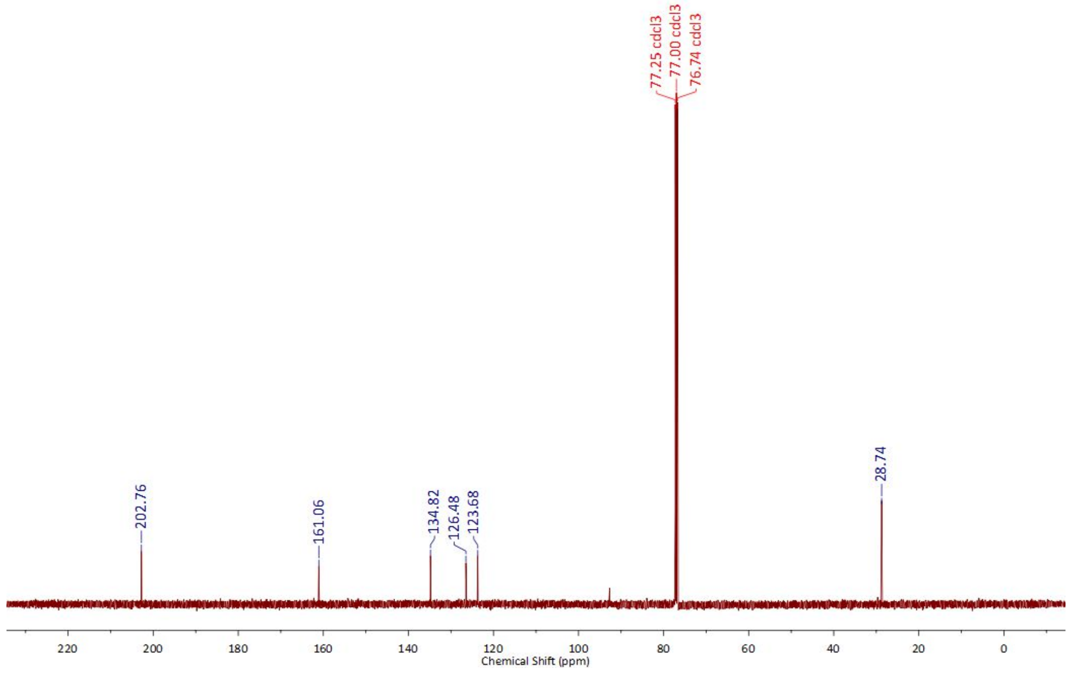

Supplementary Fig. 54. ^{13}C NMR (125 MHz) spectrum of **11** in $CDCl_3$ (T = 298 K).

Supplementary Table 13. Cartesian coordinates of the optimized geometry of ***o*-DAPA** at $S_{0,\mathrm{min}}$.

Atom	x	y	z
C	-0.681493513	-1.532685315	0.023745987
C	0.681332903	-1.532804134	-0.021528019
C	-1.409647736	-0.328579846	0.026420189
C	-0.712434550	0.883864128	-0.001460272
C	-2.888811647	-0.359757373	0.008142505
O	-3.562358921	0.652898925	-0.005236553
C	-3.592999818	-1.695992836	-0.002673363
C	0.712488623	0.883838788	0.002475179
C	1.409642888	-0.328666957	-0.025426975
N	-1.318768080	2.117432903	0.007016199
C	2.888871814	-0.359806920	-0.009064313
O	3.562470268	0.652834550	0.002593782
C	3.592895300	-1.696170978	0.001644882
N	1.318895771	2.117385147	-0.005919368
H	-1.204564831	-2.471498294	0.041164935
H	1.203904842	-2.471903010	-0.038677069
H	-4.660101806	-1.512670566	-0.009606132
H	-3.322973515	-2.277708024	-0.880683749
H	-3.335316413	-2.284536116	0.874485508
H	-0.863601975	2.802047196	-0.570652416
H	-2.317924684	2.080661731	-0.090596988
H	4.660014940	-1.513041442	0.010725846
H	3.320399000	-2.278664393	0.878208256
H	3.336493590	-2.283148469	-0.876946506
H	2.318109386	2.080858624	0.090965130
H	0.863874434	2.802531416	0.571218540

Supplementary Table 14. Cartesian coordinates of the optimized geometry of ***o*-DAPA** at $S_{1,\mathrm{min}}$.

Atom	x	y	z
C	-0.702520017	1.491224530	-0.007936608
C	0.702367568	1.491341638	-0.000530139
C	-1.464318327	0.341154722	-0.018598076
C	-0.697051115	-0.893388750	0.034287556
C	-2.911190665	0.364847458	-0.016870154
O	-3.594380739	-0.665564235	0.054704125
C	-3.622054568	1.696118731	-0.104056145
C	0.697055376	-0.893309603	-0.035748749
C	1.464251924	0.341432550	0.014880311
N	-1.352696508	-2.077199633	0.137518743
C	2.911124028	0.365009409	0.017467781
O	3.594576341	-0.665085141	-0.056114653
C	3.621848627	1.695734499	0.113520188
N	1.353023710	-2.076981876	-0.135158059
H	-1.194136299	2.448586037	0.006884570
H	1.193540129	2.448912904	-0.018623443
H	-4.685666690	1.498719485	-0.157254727
H	-3.427328778	2.315071595	0.770542036
H	-3.324153205	2.264976479	-0.982493317
H	-0.904715825	-2.828418566	0.629016583
H	-2.362054469	-1.999313811	0.186935491
H	4.684553381	1.497249529	0.179567000
H	3.438939131	2.315305828	-0.763340301
H	3.313249762	2.264516605	0.988200747
H	2.362257251	-1.999200169	-0.186517927
H	0.903665633	-2.831343010	-0.620656283

Supplementary Table 15. Cartesian coordinates of the optimized geometry of ***o*-DAPA** at $S_{1,2pt}$.

Atom	x	y	z
C	0.705935608	1.510344888	0.012439005
C	-0.682319849	1.515560682	0.005986324
C	1.470016051	0.339775111	0.009109470
C	0.722496179	-0.954551661	-0.005649279
C	2.851778471	0.386695217	0.004645840
O	3.591700038	-0.688830053	-0.016863183
C	3.648446792	1.655255256	0.013405190
C	-0.719389896	-0.948444817	0.012124731
C	-1.460774949	0.350648090	-0.002417800
N	1.468663009	-2.022404487	-0.032581681
C	-2.847206476	0.396260208	-0.009858523
O	-3.588649078	-0.685287021	0.000391745
C	-3.682878394	1.632098748	-0.024738758
N	-1.465984164	-2.020152620	0.039695882
H	1.203215742	2.465689179	0.016178564
H	-1.163529513	2.477105164	0.010584407
H	4.696852768	1.400881958	0.110369538
H	3.514736890	2.214546312	-0.910397096
H	3.369906829	2.304479427	0.839046853
H	0.959276964	-2.891680979	-0.057497409
H	2.953107869	-1.480499209	-0.030701307
H	-4.378767275	1.571762997	-0.858642055
H	-4.281800932	1.668449578	0.883794209
H	-3.122833377	2.552679139	-0.107722971
H	-2.955902124	-1.474917264	0.021111392
H	-0.952579373	-2.887092877	0.067289763

Supplementary Table 16. Cartesian coordinates of the optimized geometry of ***o*-DAPA** at CI_{10}.

Atom	x	y	z
C	-0.713905000	-1.110804578	-0.933337207
C	0.698526307	-1.328821826	-0.597779408
C	-1.474006009	-0.391227495	-0.018876671
C	-0.683800729	0.868862462	-0.187784316
C	-2.825917928	-0.432827337	0.360816429
O	-3.429301220	0.595268351	0.728861301
C	-3.561231321	-1.748085883	0.280444794
C	0.711346836	0.941297028	-0.057697134
C	1.455007695	-0.315673659	-0.135320891
N	-1.392484166	1.962384652	-0.558598352
C	2.866720557	-0.424359157	0.238280912
O	3.521293288	0.561464722	0.526922780
C	3.521429415	-1.783441202	0.239333813
N	1.337448197	2.134993113	-0.026481674
H	-1.143267870	-1.693235535	-1.738024229
H	1.098397389	-2.327437769	-0.701250988
H	-4.159972510	-1.871332973	1.178430277
H	-4.247395344	-1.741907981	-0.565568488
H	-2.891631826	-2.596714921	0.175158731
H	-1.102152991	2.513923151	-1.345535569
H	-2.376110669	1.923990223	-0.330390531
H	4.567585197	-1.659294520	0.490099418
H	3.050829161	-2.439658667	0.967154319
H	3.440949723	-2.258887012	-0.735219627
H	2.324131499	2.104092956	0.171932913
H	0.812765465	2.925455657	0.299707806

Supplementary Table 17. Cartesian coordinates of the optimized geometry of ***o*-DAPA** at $CI_{10,1pt}$.

Atom	x	y	z
C	-0.607614637	-1.139526689	0.958132251
C	0.767844289	-1.418798043	0.689917460
C	-1.434708577	-0.263996916	0.109114568
C	-0.743211961	1.025411971	-0.020534337
C	-2.715111751	-0.464470121	-0.236592774
O	-3.467740707	0.484067262	-0.820645755
C	-3.477138202	-1.725704577	-0.021975597
C	0.649199604	0.846280670	0.345119100
C	1.446070289	-0.434864068	0.058308163
N	-1.364556735	2.134253763	-0.195800019
C	2.779185022	-0.354479547	-0.431516380
O	3.289705662	0.744133623	-0.664397502
C	3.588435860	-1.612877135	-0.614907847
N	1.350465388	1.740279165	1.020619415
H	-1.083328097	-1.675890911	1.765879621
H	1.211273130	-2.345429237	1.038850464
H	-4.287544870	-1.564972564	0.687089000
H	-3.931903436	-2.035042521	-0.960659841
H	-2.844497717	-2.528550171	0.338929210
H	-0.746194539	2.935250387	-0.174359887
H	-2.985754206	1.327373448	-0.781367065
H	3.824269137	-1.724812118	-1.670458435
H	4.530166925	-1.512330180	-0.082404103
H	3.076920410	-2.510561982	-0.283936719
H	2.341808172	1.787378409	0.820925254
H	0.904288123	2.520893836	1.466922390

Supplementary Table 18. Cartesian coordinates of the optimized geometry of ***m*-DAPA** at $S_{0,\text{min}}$.

Atom	x	y	z
C	-1.220051916	-1.295191876	0.001349394
C	0.000068862	-1.962738484	-0.000203651
C	-1.228924522	0.137708651	0.001381114
C	0.000011343	0.777824127	0.001883388
N	-2.369944006	-1.990701392	0.002077391
C	-2.476644374	0.898927729	-0.000638427
O	-3.574712599	0.364259753	0.002138995
C	-2.421066446	2.411651113	-0.008399347
C	1.228844453	0.137517106	0.001029472
C	1.220217256	-1.295321454	-0.000651325
C	2.476467283	0.899044094	0.000165664
C	2.420788449	2.411708900	0.008764320
O	3.574637101	0.364596063	-0.006325809
N	2.370312555	-1.990565666	-0.000117742
H	-0.000233850	-3.042357937	0.000018264
H	0.000591954	1.852074923	0.001846634
H	-3.237094855	-1.486134535	0.004111143
H	-2.355568278	-2.988889204	0.001554864
H	-1.908383777	2.795851026	0.870386222
H	-1.901185498	2.785784990	-0.887034443
H	-3.438831260	2.781187113	-0.014682472
H	1.907898668	2.785366699	0.891849970
H	3.438316218	2.781608619	0.006534560
H	1.900769156	2.796641313	-0.865356248
H	2.355921316	-2.988555007	-0.015313763
H	3.237326038	-1.485701060	-0.010426172

Supplementary Table 19. Cartesian coordinates of the optimized geometry of ***m*-DAPA** at CI_{21}.

Atom	x	y	z
C	-1.219337797	-1.280202344	-0.035188833
C	0.001349965	-1.969830455	-0.040196755
C	-1.241926344	0.148939627	-0.134807948
C	-0.000617084	0.802822405	-0.424980289
N	-2.363495940	-1.958870824	0.079558240
C	-2.481379740	0.884225604	0.042584630
O	-3.590202134	0.334402757	0.102149460
C	-2.408428213	2.386332754	0.105082443
C	1.242082900	0.148451000	-0.135883455
C	1.221893112	-1.280030262	-0.037003279
C	2.480253788	0.886185080	0.043790237
C	2.403750314	2.388222448	0.104260708
O	3.589735895	0.338819665	0.104838455
N	2.366779547	-1.958818266	0.076407637
H	0.001119046	-3.046551360	0.015903045
H	0.000012554	1.837476329	-0.702778382
H	-3.219615514	-1.416500493	0.102718315
H	-2.358802861	-2.948456031	0.225077452
H	-1.648830866	2.721913316	0.808952362
H	-2.153681728	2.808506131	-0.867583549
H	-3.379194883	2.765332771	0.401630097
H	1.634860261	2.723560479	0.797825937
H	3.369747953	2.770857569	0.411010001
H	2.159494919	2.806525206	-0.872765386
H	2.362029265	-2.947572196	0.227078809
H	3.222727183	-1.417038767	0.101214804

Supplementary Table 20. Cartesian coordinates of the optimized geometry of ***m*-DAPA** at $S_{1,1pt}$.

Atom	x	y	z
C	-1.223152252	-1.304839236	0.001507520
C	0.024228137	-1.946987850	-0.000479998
C	-1.248537956	0.152493727	0.000564309
C	-0.025512414	0.802766912	0.000891285
N	-2.358312006	-1.971260193	0.003715484
C	-2.468648747	0.861470316	-0.001016198
O	-3.664890333	0.283736780	0.001026711
C	-2.527891786	2.347640236	-0.005485792
C	1.269041355	0.160300512	0.000350444
C	1.279957059	-1.231968847	-0.001499674
C	2.509011137	0.892544101	0.000875215
C	2.465806285	2.402370774	0.004204154
O	3.612745926	0.338120636	-0.000316914
N	2.396829358	-1.954800152	-0.003702180
H	0.048888454	-3.025825388	-0.000291924
H	-0.029792520	1.877018896	0.000650232
H	-3.535968333	-0.689905319	0.003498573
H	-2.212786634	-2.971953971	0.004478120
H	-2.040236221	2.774459805	0.871881192
H	-2.032462219	2.769230747	-0.880953486
H	-3.565064781	2.661353233	-0.010759996
H	1.951064146	2.780985486	0.885429229
H	3.483338529	2.772943291	-0.000608110
H	1.941275306	2.786243817	-0.868959415
H	2.369916788	-2.953911361	-0.009653408
H	3.267672841	-1.437244596	-0.005104289

Supplementary Table 21. Cartesian coordinates of the optimized geometry of ***m*-DAPA** at $S_{1,2pt}$.

Atom	x	y	z
C	1.259946608	1.297837693	0.000520746
C	0.000168504	1.967939548	-0.001650327
C	1.255867190	-0.168016350	0.002733953
C	0.000062271	-0.818794602	0.007512304
N	2.378299954	1.958716732	0.000019830
C	2.443393791	-0.876601971	-0.000264229
O	3.642929118	-0.305330357	-0.000858001
C	2.505213883	-2.367353579	-0.003307251
C	-1.255609470	-0.167811674	0.002371592
C	-1.259646979	1.298038584	-0.000445598
C	-2.443254078	-0.876343350	-0.000497205
C	-2.506133772	-2.367001203	-0.003223223
O	-3.642717791	-0.304913040	0.000851199
N	-2.378143052	1.958635890	-0.002180131
H	0.000492255	3.047456070	-0.003561165
H	-0.000661455	-1.892435109	0.015920999
H	3.527129893	0.669235274	0.000958039
H	2.235730447	2.959601894	-0.002216015
H	2.036742600	-2.788123708	0.885342375
H	1.993050799	-2.786555704	-0.868003349
H	3.542654032	-2.678854285	-0.028848458
H	-2.065478262	-2.787636049	0.899946081
H	-3.542818138	-2.677210326	-0.058657740
H	-1.969299126	-2.788334521	-0.851480751
H	-2.236440233	2.959641913	-0.005621739
H	-3.526731428	0.669654762	0.001685953

Supplementary Table 22. Cartesian coordinates of the optimized geometry of ***m*-DAPA** at $CI_{10,1pt}$.

Atom	x	y	z
C	1.225635964	-0.047926392	-0.013176239
C	1.133126340	1.394730437	-0.038572760
C	-0.154315254	1.985664512	-0.085162964
C	-1.325952819	1.243855581	-0.040877303
C	-1.222385921	-0.177582852	0.030349129
C	0.060513521	-0.761967711	0.053514429
H	-0.220555622	3.062431422	-0.134347684
H	0.131968749	-1.835094171	0.093516873
N	-2.528269076	1.873505960	-0.032507670
H	-3.349444609	1.311148344	-0.150690763
H	-2.561909997	2.839029955	-0.287348587
N	2.256404995	2.074850486	0.031867840
H	2.058092685	3.070408766	0.003427547
H	2.934978063	-0.300215970	1.755494055
C	2.545632204	-0.697155029	-0.059355495
O	3.177278537	-0.968489332	1.111515651
C	-2.418999729	-1.034076879	0.038432046
O	-3.545524840	-0.575597222	-0.038050802
C	3.058303102	-1.455627702	-1.220313616
H	4.144704593	-1.397656076	-1.262303691
H	2.800936451	-2.521247835	-1.178153346
H	2.652355519	-1.057509734	-2.145225696
C	-2.255248911	-2.533275578	0.141114208
H	-3.243201964	-2.975560553	0.165557341
H	-1.708535711	-2.929864767	-0.711051661
H	-1.713164582	-2.813704434	1.040686021

Supplementary Table 23. Cartesian coordinates of the optimized geometry of ***m*-DAPA** at $CI_{10,2pt}$.

Atom	x	y	z
C	1.279356684	1.275605691	-0.002465154
C	0.000100855	1.958089310	-0.002818545
C	1.277654445	-0.182254621	0.000220154
C	-0.000145440	-0.840780621	0.003937032
N	2.351776903	1.997264145	-0.003773998
C	2.462053755	-0.874066867	0.000857458
O	3.683491082	-0.305409852	-0.000109027
C	2.519276110	-2.363047557	0.003769796
C	-1.277832565	-0.182048399	0.000657907
C	-1.279192556	1.275792122	0.001149874
C	-2.462086838	-0.874033857	-0.001666640
C	-2.519210998	-2.362938343	-0.007113678
O	-3.683770107	-0.305480731	0.001539775
N	-2.351440733	1.997779387	0.005231132
H	0.000323041	3.037822213	-0.005622582
H	-0.001045652	-1.911990624	0.011348094
H	3.592606365	0.657337631	-0.001633236
H	2.154978940	2.989672009	-0.004763973
H	2.031178994	-2.779388033	0.884862486
H	2.015882034	-2.783113286	-0.866784975
H	3.554740688	-2.683306652	-0.004284466
H	-2.051838057	-2.784546404	0.882899297
H	-3.554084925	-2.683362238	-0.040656828
H	-1.995690638	-2.778717235	-0.867715497
H	-2.153959651	2.990063350	0.005045268
H	-3.593017561	0.657158934	0.005691569

Supplementary Table 24. Cartesian coordinates of the optimized geometry of ***p*-DAPA** at $S_{0,\mathrm{min}}$.

Atom	x	y	z
C	-0.798709973	1.181046057	-0.018951092
C	-1.401099708	-0.098762839	-0.014336186
C	0.589551098	1.229337931	-0.000781530
C	1.401122029	0.098722429	0.014621635
C	0.798724919	-1.181067995	0.019156114
C	-0.589551960	-1.229342228	0.000951486
N	-1.520367944	2.348394296	-0.098706741
N	1.520426038	-2.348340692	0.098836337
C	2.873816680	0.255129557	-0.007462180
O	3.620986841	-0.699909671	-0.086250540
C	3.471448215	1.641187438	0.059839791
C	-2.873856760	-0.255143485	0.007493275
O	-3.621057544	0.699798026	0.087092658
C	-3.471448640	-1.641027716	-0.061391877
H	1.049316383	2.202801371	-0.017411150
H	-1.049280460	-2.202809829	0.017563070
H	-2.495722773	2.271335587	0.116362239
H	-1.062662769	3.172942299	0.236012675
H	2.495867701	-2.271189750	-0.115891976
H	1.062660796	-3.172938120	-0.235708699
H	4.549147981	1.541067754	0.088642342
H	3.134247535	2.175355191	0.944330333
H	3.191933992	2.230734569	-0.810188653
H	-4.549104618	-1.540879336	-0.090533945
H	-3.192594835	-2.231950197	0.808024351
H	-3.133445172	-2.174384864	-0.946119686

Supplementary Table 25. Cartesian coordinates of the optimized geometry of ***p*-DAPA** at $S_{1,\mathrm{min}}$.

Atom	x	y	z
C	0.789407237	-1.151383553	-0.001286089
C	1.453753873	0.126992923	0.000712730
C	-0.609435595	-1.233405253	-0.002692339
C	-1.453773800	-0.127019666	-0.001034620
C	-0.789401644	1.151356957	0.000271529
C	0.609439194	1.233412801	0.002270366
N	1.530458852	-2.267249663	-0.002701473
N	-1.530425649	2.267208004	0.000793487
C	-2.895382261	-0.244392905	-0.000279085
O	-3.651592317	0.739191696	-0.003105929
C	-3.510273063	-1.623830178	0.004978012
C	2.895328215	0.244369543	0.000953807
O	3.651609562	-0.739156927	0.002940192
C	3.510304242	1.623844605	-0.001982594
H	-1.037866015	-2.221860254	-0.005411657
H	1.037756023	2.221936524	0.005153289
H	2.537920222	-2.148774730	-0.000072098
H	1.101431355	-3.170909267	-0.003735634
H	-2.537940946	2.148954066	-0.002107031
H	-1.101633846	3.170997965	0.001621457
H	-4.587082503	-1.510500346	0.029640006
H	-3.197374339	-2.203327908	0.872186675
H	-3.237099262	-2.191338809	-0.883635790
H	4.587174101	1.510713776	-0.025654577
H	3.236134463	2.190181317	0.887097537
H	3.198245798	2.204606310	-0.868704095

Supplementary Table 26. Cartesian coordinates of the optimized geometry of ***p*-DAPA** at $S_{1,1pt}$.

Atom	x	y	z
C	-0.814214401	-1.182208854	0.004984030
C	-1.460091012	0.142875137	0.001666350
C	0.623748189	-1.247075863	0.003526311
C	1.465571888	-0.153690675	0.001788120
C	0.783781858	1.165832137	0.003019541
C	-0.595819451	1.271170574	0.000118646
N	-1.581614114	-2.216861445	0.009473389
N	1.557203731	2.245277940	0.004523635
C	2.896538357	-0.256985171	-0.001670891
O	3.656123451	0.733167991	0.005701376
C	3.519351552	-1.632404664	-0.016755744
C	-2.827414746	0.268461279	-0.001799479
O	-3.644354347	-0.755809023	-0.001876468
C	-3.535997865	1.585501359	-0.010116231
H	1.042174521	-2.239399809	0.004575041
H	-1.015952102	2.263729489	-0.003521483
H	-3.079487559	-1.586330526	0.002577238
H	-1.071315794	-3.088472808	0.012355943
H	2.566029342	2.078173247	0.007085929
H	1.161579866	3.165479642	0.004794154
H	4.595538312	-1.512904878	-0.044713943
H	3.205444674	-2.208384315	-0.885803769
H	3.251932923	-2.206654138	0.869159554
H	-4.604138736	1.410366608	0.027783165
H	-3.307828974	2.150810712	-0.911488348
H	-3.251880009	2.196795163	0.843465262

Supplementary Table 27. Cartesian coordinates of the optimized geometry of ***p*-DAPA** at $CI_{10,1pt}$.

Atom	x	y	z
C	0.893575259	1.157080850	0.129520770
C	1.431346413	-0.131305068	-0.003391240
C	-0.539455363	1.312758048	0.136033740
C	-1.358663726	0.261089658	-0.035042304
C	-0.833229784	-1.078844708	-0.088433412
C	0.541528991	-1.227172023	-0.094606453
N	1.643960996	2.266138162	0.198949022
N	-1.795368147	-2.027680943	-0.063611274
C	-2.848874663	0.309020435	-0.004366371
O	-3.375962863	-0.392432531	0.963522947
C	-3.693345284	1.011240567	-0.992290948
C	2.882707463	-0.326944441	-0.053821909
O	3.669786732	0.604199034	0.028212763
C	3.430526378	-1.725030491	-0.214070136
H	-0.939158642	2.310645270	0.233508703
H	0.944094483	-2.218523052	-0.217778339
H	2.644005074	2.163592731	0.179706534
H	1.219414481	3.158291768	0.345361217
H	-2.875154552	-1.306803826	0.872256831
H	-1.391494338	-2.952563071	0.012894432
H	-4.654551243	0.511779671	-1.074157464
H	-3.212660523	1.037001106	-1.968965704
H	-3.888642178	2.051260398	-0.709217339
H	4.511498320	-1.664879809	-0.231873730
H	3.079979133	-2.180554957	-1.137354047
H	3.121041133	-2.366873519	0.607534928

Supplementary Table 28. Cartesian coordinates of the optimized geometry of ***p*-DAPA** at $CI_{10,2pt}$.

Atom	x	y	z
C	-0.869825174	1.165658760	0.057134762
C	-1.487064826	-0.160764692	0.024391883
C	0.611984716	1.260014143	0.039755559
C	1.414670415	0.202239565	0.013552217
C	0.786648268	-1.144454221	0.161796893
C	-0.552528957	-1.275237975	0.168718257
N	-1.569022624	2.230972191	0.073278315
N	1.733471686	-2.069162736	0.432998961
C	2.857702778	0.275014591	-0.122947081
O	3.474007277	-0.731858921	-0.668419092
C	3.681232190	1.452682736	0.251705230
C	-2.830251839	-0.302431274	-0.076288352
O	-3.692636519	0.716484793	-0.136498834
C	-3.558626884	-1.602677931	-0.135932545
H	1.010384638	2.263183202	-0.004271311
H	-0.949414138	-2.251328159	0.392210323
H	-3.197517338	1.552219019	-0.059084969
H	-1.005966534	3.070899082	0.090909143
H	2.914725038	-1.575281611	-0.349598726
H	1.337803846	-2.992538676	0.552484519
H	4.560292316	1.108822377	0.792567682
H	3.141246040	2.156839867	0.875162797
H	4.042712333	1.977757322	-0.634125947
H	-4.227188110	-1.590821978	-0.993521803
H	-4.180471422	-1.717454994	0.750145091
H	-2.903977697	-2.459166172	-0.219701606

Supplementary Table 29. Cartesian coordinates of the optimized geometry of **5** at $S_{0,min}$.

Atom	x	y	z
C	-1.413222678	0.090881959	0.000491950
C	-0.769472484	-1.138635272	0.000290052
C	0.615317231	-1.257126971	0.000938905
C	1.412628468	-0.090804405	0.001297732
C	0.769094346	1.138627930	0.000198982
C	-0.615849395	1.257159796	0.000092129
N	-2.800348340	0.197661238	0.003463147
N	2.799856546	-0.198471161	0.002631984
C	-3.730002134	-0.800817675	0.012188899
C	-5.156433064	-0.308416307	-0.027659680
C	3.729614537	0.798829260	-0.003133469
C	5.158267910	0.311298312	-0.005990200
O	3.471661246	1.982802049	-0.008688736
O	-3.470758913	-1.984211529	0.025928976
C	-1.200345797	2.626723255	0.001203071
C	-0.280268099	3.820278128	0.015428059
O	-2.402419514	2.812818178	-0.007120839
C	1.199812844	-2.626908930	0.000467596
O	2.401790213	-2.813259625	0.012148204
C	0.279757446	-3.820416884	-0.017235769
H	-1.385535037	-2.012978528	0.000680849
H	1.385171608	2.013041434	-0.000246831
H	-3.126805297	1.149507607	-0.005390932
H	3.125144559	-1.150460284	0.007092151
H	-5.764706208	-0.964324788	0.584122890
H	-5.265672041	0.716018998	0.313564091
H	-5.522636599	-0.372959022	-1.049691116
H	5.673629586	0.747817356	0.843933063
H	5.251129983	-0.768661438	0.035543900
H	5.646870284	0.676273305	-0.904586075
H	-0.891182539	4.713965615	0.008639752
H	0.377253410	3.823521952	-0.849690331
H	0.350439235	3.818755425	0.900571687
H	0.890753384	-4.714107134	-0.009839862
H	-0.348554222	-3.818010272	-0.904119095
H	-0.379692318	-3.824775063	0.846390060

Supplementary Table 30. Cartesian coordinates of the optimized geometry of **5** at $S_{1,min}$.

Atom	x	y	z
C	-1.395364935	0.024349169	-0.004045502
C	-0.701816250	-1.194285817	-0.002539334
C	0.686932980	-1.283765572	0.000319635
C	1.395518677	-0.024491919	0.003580005
C	0.701924953	1.194224381	0.002140530
C	-0.686722464	1.283690787	-0.003694545
N	-2.753597154	0.089862818	-0.004101187
N	2.753746156	-0.090076448	0.006202755
C	-3.695821597	-0.926503847	0.008728365
C	-5.110115891	-0.413135854	-0.010160525
C	3.695819159	0.926429553	0.009695866
C	5.110132840	0.414173046	-0.027609115
O	3.417343988	2.098868735	0.026290643
O	-3.417644797	-2.099055668	0.025054143
C	-1.354035066	2.567548880	-0.005767941
C	-0.521523540	3.824932196	0.005633523
O	-2.592220025	2.678760960	-0.015485484
C	1.354138303	-2.567766109	-0.001113462
O	2.592288342	-2.679170479	0.002441686
C	0.521142917	-3.824846572	-0.010170840
H	-1.295094796	-2.085495462	-0.002537890
H	1.295193434	2.085443688	0.005531352
H	-3.083933827	1.061287083	-0.012018347
H	3.084096943	-1.061333893	0.002113384
H	-5.786142183	-1.246658728	0.128610843
H	-5.269214183	0.325482781	0.771383956
H	-5.322326713	0.066304054	-0.963684252
H	5.786683043	1.242757818	0.135254243
H	5.274056916	-0.351216640	0.726446677
H	5.317685188	-0.031243550	-0.998606793
H	-1.196945830	4.671417888	-0.002559823
H	0.133268832	3.885551917	-0.861387359
H	0.108724897	3.883292432	0.890964589
H	1.195937279	-4.671805447	-0.002440656
H	-0.110024361	-3.883242110	-0.894839315
H	-0.132846600	-3.884683857	0.857483956

Supplementary Table 31. Cartesian coordinates of the optimized geometry of **6** at $S_{0,min}$.

Atom	x	y	z	Atom	x	y	z
C	-1.383884529	0.309738184	0.088428335	H	-1.673940803	-1.775622122	0.087578940
C	-0.934503911	-1.002897285	0.093341172	H	1.674050042	1.778545967	0.106573748
C	0.415378287	-1.334873643	0.097365881	H	-2.941407413	1.615458341	0.097397331
C	1.383638104	-0.306602251	0.095668542	H	2.940579200	-1.612650715	0.105821833
C	0.934373753	1.005985992	0.102215262	H	-0.156297287	4.795435504	0.079950766
C	-0.415581004	1.337977602	0.098129535	H	0.957565472	3.710923071	-0.770034479
N	-2.735631807	0.630037457	0.078032164	H	0.938291890	3.735156724	0.981091105
N	2.735283633	-0.627031603	0.090601336	H	0.156196113	-4.792511199	0.104773766
C	3.796631123	0.223068485	0.008657277	H	-0.941998803	-3.722273916	-0.782223373
O	3.740510463	1.428536395	-0.046355218	H	-0.954428372	-3.717689698	0.969075462
C	-3.796227454	-0.221208101	-0.001211367	H	-7.047094480	1.625475998	0.856095652
O	-3.737870090	-1.426512251	-0.059408664	H	-8.192320164	0.753575044	-0.164521380
C	-6.225718829	-0.115076285	-0.116783487	H	-6.944177504	1.762619569	-0.897124686
C	-0.781908291	2.780812384	0.102419466	H	-6.342951052	-0.493282261	2.001840355
O	-1.939829575	3.149702078	0.111284853	H	-7.509613030	-1.387704695	1.034246624
C	0.311666472	3.819250665	0.096964687	H	-5.818332825	-1.888682751	1.054941433
C	0.781613338	-2.777761041	0.104219687	H	-6.110902421	-0.170771238	-2.267542428
O	1.939515137	-3.146687047	0.115963084	H	-7.363126050	-1.197575732	-1.577522549
C	-0.311897310	-3.816246946	0.098193392	H	-5.673416834	-1.694667033	-1.488952705
C	-7.160495537	1.082860714	-0.078027773	H	7.051074121	-1.637548961	0.835690787
C	-6.482153636	-1.033626332	1.068749787	H	8.188720775	-0.765000048	-0.192654497
C	-6.343068528	-0.844825174	-1.446730441	H	6.930305989	-1.766569813	-0.917014295
C	6.225510854	0.110213283	-0.121570199	H	5.664855442	1.697344112	-1.481589046
C	7.156554955	-1.090948948	-0.097095577	H	7.354033918	1.200739596	-1.583744045
C	6.334885129	0.847293038	-1.448078045	H	6.097296494	0.178099006	-2.271470593
C	6.494152378	1.021126684	1.067109281	H	5.830110650	1.876144837	1.065189515
O	-4.914810923	0.501956396	-0.002738100	H	7.521212870	1.375435692	1.024685503
O	4.913686234	-0.502615918	0.000176979	H	6.363806301	0.474657082	1.997891826

Supplementary Table 32. Cartesian coordinates of the optimized geometry of **6** at $S_{1,min}$.

Atom	x	y	z	Atom	x	y	z
C	-1.370804078	-0.267066376	-0.072881246	H	-1.633023044	1.829313925	-0.062608787
C	-0.896695501	1.052251623	-0.076894186	H	1.633423330	-1.831540368	-0.089531179
C	0.455462396	1.381887347	-0.092684950	H	-2.881248105	-1.576559734	-0.068185463
C	1.370849588	0.264699549	-0.083565486	H	2.881143826	1.574253432	-0.084700067
C	0.896830171	-1.054582257	-0.089259694	H	-0.372091987	-4.808284247	-0.112721698
C	-0.455453843	-1.384226150	-0.090370783	H	0.806891429	-3.808403357	0.744563827
N	-2.695522055	-0.567751703	-0.052976327	H	0.774857760	-3.805660186	-1.008168970
N	2.695561474	0.565473194	-0.068290129	H	0.371546680	4.805883796	-0.125656793
C	3.781680415	-0.286729188	-0.001874085	H	-0.779721690	3.816220944	0.778688687
O	3.724966258	-1.487470881	0.048360345	H	-0.802881435	3.793137283	-0.974025350
C	-3.781340668	0.285273069	0.006049925	H	-6.983594136	-1.627682513	-0.859954264
O	-3.723666920	1.486207998	0.050622833	H	-8.152134430	-0.776073758	0.151031095
C	-6.207863154	0.136254244	0.107569706	H	-6.885184500	-1.753420469	0.894266673
C	-0.890714707	-2.763979941	-0.101570230	H	-6.307447157	0.500014370	-2.014724234
O	-2.089350322	-3.088215946	-0.099217302	H	-7.505855154	1.369825446	-1.064468861
C	0.147797352	-3.858255861	-0.119965822	H	-5.829260946	1.914220381	-1.069917558
C	0.890561408	2.761681965	-0.105037912	H	-6.102394277	0.207124978	2.258443551
O	2.089135018	3.085988339	-0.115080073	H	-7.368103676	1.207249841	1.554955453
C	-0.148166130	3.855939802	-0.107008537	H	-5.688770706	1.734799411	1.472342356
C	-7.113109449	-1.083169194	0.070757985	H	6.988098129	1.629774582	-0.855256309
C	-6.469791567	1.041376036	-1.086228259	H	8.149824479	0.783277284	0.167569372
C	-6.341567644	0.871505814	1.432051555	H	6.875851645	1.760919270	0.897837637
C	6.207607726	-0.132590871	0.112337681	H	5.680724575	-1.726143438	1.480924599
C	7.110909799	1.088369527	0.078228874	H	7.359565803	-1.197981359	1.571165515
C	6.333821378	-0.862965128	1.440371841	H	6.089434373	-0.195500506	2.262871966
C	6.479458157	-1.041103661	-1.076477038	H	5.831472450	-1.908559078	-1.067944139
O	-4.877539351	-0.454629812	0.005868082	H	7.512359793	-1.377843037	-1.039680906
O	4.876957653	0.454621209	-0.001049236	H	6.334287828	-0.499589745	-2.007774429

Supplementary Table 33. Cartesian coordinates of the optimized geometry of **7** at $S_{0,min}$.

Atom	x	y	z
C	-1.415000769	0.132746122	-0.009703033
C	-0.804497379	-1.113393808	-0.009793276
C	0.576828993	-1.273245914	-0.006136986
C	1.415119736	-0.133364319	-0.002577477
C	0.804645561	1.112763603	-0.004165638
C	-0.576678422	1.272596757	-0.006624014
N	-2.794321940	0.294143519	-0.012308822
N	2.794481544	-0.294636512	0.003941913
C	-3.754864183	-0.688515334	0.021908991
O	-3.500097258	-1.880448125	0.016730616
N	-5.036805390	-0.217097165	0.072357498
C	3.754796584	0.688636501	0.016761138
O	3.500078067	1.880006679	-0.018220464
N	5.037445044	0.219717275	0.082416093
C	-5.373860533	1.187233624	-0.002246391
C	-6.119950780	-1.167142790	-0.042040287
C	5.375262708	-1.185720516	0.040423706
C	6.117230235	1.167515945	-0.076843617
C	-1.113036995	2.658179873	-0.002744676
O	-2.309227064	2.888199889	-0.023917507
C	-0.155555954	3.822045015	0.030000254
C	1.113273656	-2.658841358	-0.010268718
O	2.309676762	-2.888684039	-0.008230355
C	0.155403526	-3.822799849	-0.020402502
H	-1.446805677	-1.968111712	-0.012841503
H	1.446863780	1.967544402	-0.003714456
H	-3.070922909	1.259277446	-0.037501285
H	3.071515558	-1.259738019	-0.014207289
H	-6.431951265	1.294781150	0.205559386
H	-5.177486090	1.615009235	-0.985780687
H	-4.836768008	1.770280356	0.740541065
H	-6.860326362	-0.983213638	0.733195057
H	-6.611038476	-1.093625034	-1.012794172
H	-5.720747515	-2.163612003	0.074522646
H	4.826634905	-1.752447604	0.787074029
H	5.193572497	-1.634003096	-0.936923043
H	6.429955374	-1.289368559	0.266977133
H	6.571226650	1.092518894	-1.065512374
H	6.885315748	0.982328420	0.670153082
H	5.724497431	2.164927543	0.053476709
H	-0.738726540	4.734272411	0.039838772
H	0.498444543	3.819737511	-0.837875757
H	0.478793793	3.786261551	0.911453457
H	0.738156632	-4.735339802	-0.022090102
H	-0.483538273	-3.798374326	-0.898995016
H	-0.494077572	-3.808414483	0.850635681

Supplementary Table 34. Cartesian coordinates of the optimized geometry of **7** at $S_{1,min}$.

Atom	x	y	z
C	1.396560587	0.093871100	0.003435452
C	0.760797627	-1.155015915	-0.006955189
C	-0.622090189	-1.312641297	-0.007350548
C	-1.397625449	-0.093396735	0.000675940
C	-0.761816181	1.155549522	0.010195471
C	0.621028730	1.313183603	0.009104336
N	2.748191645	0.237650623	0.002043862
N	-2.749307097	-0.236521407	-0.001660953
C	3.729052633	-0.752250018	0.010860323
O	3.471626102	-1.937797007	0.069551063
N	4.994554218	-0.263870418	-0.055102054
C	-3.730039937	0.753739736	0.006594618
O	-3.472872654	1.940778307	0.023651938
N	-4.994643192	0.260758149	-0.005977865
C	5.321709101	1.148901596	-0.101234702
C	6.095308133	-1.195288485	0.062541530
C	-5.311136621	-1.155655003	-0.025768749
C	-6.097663123	1.196635591	0.011436364
C	1.216449611	2.629810923	0.009874593
O	2.449556273	2.809929488	0.012640834
C	0.319775879	3.842050523	0.005862943
C	-1.217492424	-2.629367059	-0.012896948
O	-2.450394830	-2.809402364	-0.019599821
C	-0.320760210	-3.841613587	-0.009453138
H	1.398856334	-2.014207902	-0.010887489
H	-1.399968352	2.014652907	0.017839623
H	3.010513510	1.227394347	0.017177284
H	-3.012622245	-1.226167095	-0.009439252
H	6.374728583	1.240603980	-0.338259012
H	5.141112349	1.647855976	0.849314555
H	4.763377835	1.668626846	-0.872657450
H	6.765580420	-1.092911749	-0.788074848
H	6.661199962	-1.008066974	0.974014764
H	5.701551711	-2.200429960	0.088699438
H	-4.913745877	-1.651843213	-0.907331255
H	-4.937144571	-1.672025055	0.854631490
H	-6.389035359	-1.257014702	-0.041621327
H	-6.709667396	1.048558900	0.899210431
H	-6.724193930	1.061904891	-0.868011354
H	-5.702134306	2.201320400	0.015981956
H	0.951012777	4.722034636	-0.011965733
H	-0.314816633	3.879883002	0.889347043
H	-0.336792461	3.858160497	-0.861688482
H	-0.951966889	-4.721575956	0.010718055
H	0.338169705	-3.856656250	0.856228298
H	0.311305483	-3.880537864	-0.894785596

Supplementary Table 35. Cartesian coordinates of the optimized geometry of **8** at $S_{0,min}$.

Atom	x	y	z
C	-0.709580807	0.132126387	0.003627957
C	0.499421580	0.809609550	0.010042484
C	1.729752285	0.158068891	0.015668884
C	1.771702476	-1.254896306	0.027465756
C	0.551827070	-1.927664653	0.014446337
C	-0.677199351	-1.282425313	0.000553146
C	-1.913206462	-2.109455618	-0.014173456
C	2.972843237	0.964533813	-0.016966959
O	-3.024256067	-1.615104787	-0.011698580
O	4.069668521	0.443461547	-0.076817936
C	2.874799653	2.469512840	0.014645843
C	-1.800083917	-3.614421889	-0.032772269
N	-1.935237855	0.800592500	-0.003109584
N	2.935753824	-1.963408011	0.094164738
C	-2.169411805	2.141432744	-0.004526774
O	-1.308436127	2.995093296	-0.010936740
C	-3.633193059	2.512049346	0.022569996
H	0.460935663	1.879251432	0.004224506
H	0.581079065	-3.003196113	0.024295976
H	3.880015289	2.872067631	0.015559214
H	2.344520902	2.813597870	0.898831106
H	2.336757808	2.847283665	-0.851326256
H	-2.802131817	-4.023086013	-0.061303402
H	-1.289058612	-3.981772124	0.853686004
H	-1.246394910	-3.959054661	-0.902169836
H	-2.727861323	0.183135713	-0.001139686
H	2.907244976	-2.925673820	-0.175135642
H	3.781303092	-1.456607410	-0.085740263
H	-3.814979989	3.258852585	-0.743063357
H	-4.299649261	1.669303639	-0.129781873
H	-3.857774454	2.968040276	0.983116176

Supplementary Table 36. Cartesian coordinates of the optimized geometry of **8** at $S_{1,min}$.

Atom	x	y	z
C	0.677201722	-0.153727822	-0.000177867
C	-0.557081695	-0.812243795	0.002138075
C	-1.777639251	-0.149359764	0.002130485
C	-1.712569091	1.284441705	-0.004280857
C	-0.474570771	1.949254982	-0.005119764
C	0.754682902	1.295465397	-0.000393095
C	2.001931558	2.019089181	0.001807658
C	-3.042160402	-0.864011311	0.009393604
O	3.115600390	1.451250446	0.012001600
O	-4.131686889	-0.283224673	0.008273752
C	-3.024147218	-2.372331791	0.019395322
C	1.971158639	3.527482127	-0.006148027
N	1.866578293	-0.823170665	-0.002438984
N	-2.846259251	1.988443811	-0.009176953
C	2.114871082	-2.179342927	-0.020428281
O	1.252290852	-3.024028360	-0.054587730
C	3.580950793	-2.519094580	0.040501818
H	-0.528552575	-1.883139074	0.002694642
H	-0.499261157	3.025723275	-0.009546131
H	-4.049503744	-2.720602518	0.034762779
H	-2.502305125	-2.761611288	0.891003755
H	-2.525210758	-2.772414048	-0.860831953
H	2.995079106	3.880559358	-0.015792658
H	1.472120953	3.931452894	0.874413881
H	1.457830184	3.923167100	-0.882062134
H	2.662733734	-0.177503834	0.010073455
H	-2.832796599	2.989540415	-0.011807402
H	-3.714332021	1.461152588	-0.005764480
H	3.733006720	-3.492330281	-0.409655001
H	4.199391124	-1.777955503	-0.456096822
H	3.891316403	-2.572546582	1.082354070

Supplementary Table 37. Cartesian coordinates of the optimized geometry of **9** at $S_{0,min}$.

Atom	x	y	z
C	-0.469179131	-0.468477431	0.057802128
C	-1.067867281	0.781347151	0.062084691
C	-2.448009687	0.958983903	0.025340970
C	-3.296416086	-0.170862498	-0.002528954
C	-2.687099459	-1.422823033	-0.006969616
C	-1.310525116	-1.604941529	0.019621739
C	-0.774697784	-2.992130835	0.007187182
O	0.415637211	-3.226225278	0.074300339
C	-1.731389472	-4.155583042	-0.094957498
C	-2.997845122	2.334842821	-0.007978841
C	-2.053806084	3.507357616	0.094802239
O	-4.190152162	2.543506089	-0.120606846
N	-4.659270746	-0.078682589	0.022283974
N	0.915111508	-0.628164236	0.088981753
C	1.867879962	0.339896630	0.026186628
O	1.673294131	1.530477639	-0.047295494
C	4.294697701	0.521307956	-0.029146164
C	4.412346892	1.469824606	1.155077469
C	4.357400073	1.252615447	-1.362510166
C	5.366057725	-0.554681495	0.040058344
O	3.065736688	-0.246590089	0.060831505
H	-0.422554587	1.634967930	0.082602937
H	-3.331765582	-2.284209910	-0.023652866
H	-1.145477629	-5.065579272	-0.121135293
H	-2.404951986	-4.188612797	0.757645075
H	-2.337093278	-4.090431354	-0.995000346
H	-2.643773011	4.415253694	0.081632835
H	-1.472771656	3.463991712	1.012396668
H	-1.349905447	3.522687085	-0.733520082
H	-5.048275254	0.823354548	-0.175014513
H	-5.178851538	-0.877374203	-0.281065960
H	1.237185555	-1.580362649	0.119911753
H	3.649818397	2.237347763	1.120974323
H	5.389601108	1.946277646	1.141178836
H	4.318441116	0.919497200	2.088024680
H	3.588596874	2.012079562	-1.430940927
H	5.329089411	1.728465953	-1.469837396
H	4.232913499	0.549094608	-2.182088065
H	5.296559766	-1.103894711	0.974756196
H	6.352180224	-0.102811019	-0.023479456
H	5.253726051	-1.258601678	-0.779587568

Supplementary Table 38. Cartesian coordinates of the optimized geometry of **9** at $S_{1,min}$.

Atom	x	y	z
C	-0.472609847	-0.446273207	0.025333190
C	-1.111099901	0.799073177	0.023356739
C	-2.491860650	0.953281152	0.004385199
C	-3.256734675	-0.261769363	-0.016097378
C	-2.619311367	-1.512590492	-0.013390925
C	-1.236511821	-1.678349469	0.008812873
C	-0.626246746	-2.985818984	0.009618652
O	0.608465912	-3.156404335	0.036872284
C	-1.515209097	-4.205343245	-0.023184790
C	-3.121480450	2.260532974	0.005726511
C	-2.245885937	3.489260198	0.031090147
O	-4.348705433	2.407461695	-0.012910044
N	-4.590002083	-0.191384601	-0.038012028
N	0.884381243	-0.576062557	0.042828932
C	1.846242859	0.408867607	0.022562756
O	1.635866637	1.594529077	-0.015775243
C	4.271255145	0.588536581	-0.017006694
C	4.381150543	1.513438519	1.186015523
C	4.336817006	1.342563531	-1.336764950
C	5.335052013	-0.494720504	0.038350499
O	3.034019224	-0.177757663	0.050926291
H	-0.478895235	1.663630827	0.034827243
H	-3.254068027	-2.382172750	-0.029078454
H	-0.875938767	-5.079530782	-0.040581986
H	-2.162029820	-4.263236415	0.851660986
H	-2.155050096	-4.222421537	-0.904716338
H	-2.890915110	4.359217861	0.039479984
H	-1.608046305	3.510053950	0.912327051
H	-1.595530169	3.536324293	-0.839976376
H	-4.999852503	0.737936953	-0.037585703
H	-5.151377297	-1.020190761	-0.053161771
H	1.197348056	-1.552165317	0.052587759
H	3.626495837	2.289230968	1.159566022
H	5.362428649	1.981485169	1.190847676
H	4.274244822	0.946618182	2.107468957
H	3.568795363	2.103947680	-1.395357657
H	5.308063306	1.821580953	-1.431164873
H	4.217055580	0.653730442	-2.169185354
H	5.261125057	-1.056893796	0.964771368
H	6.323206224	-0.046502569	-0.016813499
H	5.220451727	-1.186017846	-0.791471206

Supplementary Table 39. Cartesian coordinates of the optimized geometry of **10** at $S_{0,min}$.

Atom	x	y	z
C	-0.185386002	0.240976644	-0.004034385
C	0.722280728	-0.808747758	-0.018682170
C	2.099948404	-0.617017822	-0.022578110
C	2.626060138	0.695370614	-0.024846170
C	1.712159937	1.742769342	-0.005374630
C	0.333539696	1.558440820	0.006717484
N	3.966371220	0.961292966	-0.092478462
N	-1.563707654	0.044447610	-0.001114045
C	-0.539155714	2.758123928	0.024038852
O	-1.754491733	2.680339401	0.022633638
C	0.085381611	4.132705879	0.043322872
C	2.988959211	-1.803559830	0.006236147
O	4.197900828	-1.695030842	0.074251795
C	2.376848389	-3.181974786	-0.039651846
C	-2.239951898	-1.151094337	0.010130127
O	-1.690294058	-2.238536140	0.049702174
N	-3.599553935	-1.021155905	-0.030059953
C	-4.276925132	0.254747201	-0.091378272
C	-4.412124758	-2.212855975	0.051318565
H	0.318648257	-1.799426875	-0.016824482
H	2.108127816	2.743172598	-0.010407602
H	4.265842476	1.866301469	0.209714394
H	4.580472625	0.192390982	0.097170983
H	-2.080017102	0.904637791	0.013264066
H	-0.714617557	4.862027598	0.059283626
H	0.703290186	4.297184945	-0.835514567
H	0.712635595	4.269275259	0.920415660
H	3.180660639	-3.907584272	-0.028156287
H	1.777122260	-3.317786082	-0.935894803
H	1.724335862	-3.352491181	0.812794923
H	-5.336207670	0.072448263	-0.226618662
H	-3.936806281	0.854863738	-0.932342839
H	-4.150806329	0.838796701	0.820033676
H	-5.034045185	-2.316278105	-0.836429397
H	-3.759123915	-3.069673418	0.127195342
H	-5.061253468	-2.178846449	0.925277710

Supplementary Table 40. Cartesian coordinates of the optimized geometry of **10** at $S_{1,min}$.

Atom	x	y	z
C	-0.171327453	-0.210675518	-0.009792129
C	0.777153186	0.816842744	-0.000007115
C	2.149906970	0.594260859	0.005578233
C	2.564964742	-0.777237010	0.000703552
C	1.614211594	-1.812943030	-0.008319277
C	0.239928762	-1.603668308	-0.012444016
N	3.867637743	-1.071593977	0.004716417
N	-1.515816805	0.013441617	-0.009159161
C	-0.691589046	-2.704167062	-0.016359638
O	-1.933719223	-2.544491341	-0.023631900
C	-0.158097422	-4.115377184	-0.013280053
C	3.106278190	1.686425170	0.015343979
O	4.327202035	1.499846367	0.022937277
C	2.589713033	3.103873469	0.018374679
C	-2.188791091	1.229313597	-0.015205297
O	-1.619764076	2.302101679	-0.075941709
N	-3.540488196	1.105541925	0.055795416
C	-4.238058820	-0.164681580	0.116363714
C	-4.346084193	2.301423772	-0.053440329
H	0.394351287	1.816932245	-0.000232840
H	1.993955253	-2.820588746	-0.011124710
H	4.181691202	-2.021881479	0.002675410
H	4.515258830	-0.290042097	0.012276177
H	-2.035381378	-0.868643017	-0.025719784
H	-1.005089242	-4.790500822	0.005924566
H	0.467902584	-4.316752048	0.855472828
H	0.437277141	-4.330417307	-0.900493159
H	3.441792172	3.772464027	0.009375852
H	1.986568213	3.306736182	0.900821761
H	1.966975214	3.306110084	-0.850181928
H	-5.276834850	0.034832573	0.351277885
H	-3.840546477	-0.808173132	0.894156570
H	-4.199538201	-0.705228954	-0.827823435
H	-4.992860554	2.401601840	0.815755543
H	-3.691924470	3.158487138	-0.111320817
H	-4.969521021	2.264844243	-0.945709785

Supplementary References

1. Bryant, J. J. et al. Alkynylated phenazines: synthesis, characterization, and metal-binding properties of their bis-triazolyl cycloadducts. *J. Org. Chem.* **77**, 7479–7486 (2012).

2. Clentsmith, G. K. B., Field, L. D., Messerle, B. A., Shasha, A. & Turner, P. Intramolecular cyclization of ortho-alkynylanilines by Rh(I)-catalyzed hydroamination to yield benzo(dipyrroles) *Tetrahedron Lett.* **50**, 1469–1471 (2009).

3. Nakamura, T., Furukawa, S. & Nakamura, E. Benzodipyrrole-based donor–acceptor-type boron complexes as tunable near-infrared-absorbing materials. *Chem. Asian J.* **11**, 2016–2020 (2006).

4. M. Ramadan, R. & K. Abu Al-Nasr, A. Unusual catalytic process involving OH and NH exchange. *Int. J. Org. Chem.* **2**, 64–70 (2012).

5. Sheldrick, G. M. *SHELXT* - Integrated space-group and crystal-structure determination, *Acta Cryst.* **A71**, 3–8, (2015).

6. Sheldrick, G. M. Crystal structure refinement with *SHELXL*, *Acta Cryst.* **C71**, 3–8 (2015).

7. Dolomanov, O. V., Bourhis, L. J., Gildea, R. J., Howard, J. A. K. & Puschmann, H. *OLEX2*: a complete structure solution, refinement and analysis program, *J. Appl. Cryst.* **42**, 339–341 (2009).

8. Shin, J. W., Eom, K. & Moon, D. BL2D-SMC, the supramolecular crystallography beamline at the Pohang Light Source II, Korea. *J. Synchrotron Rad.* **23**, 369–373 (2016).

9. Otwinowski, Z. & Minor, W. Processing of X-ray diffraction data collected in oscillation mode. *Methods Enzymol.* **276**, 307–326 (1997).

10. Hansch, C., Leo, A. & Taft, R. W. A survey of Hammett substituent constants and resonance and field parameters. *Chem. Rev.* **91**, 165–195 (1991).

11. Shao, Y., Head-Gordon, M. & Krylov, A. I. The spin-flip approach within time-dependent density functional theory: Theory and applications to diradicals. *J. Chem. Phys.* **118**, 4807–4818 (2003).

12. Casida, M. E. & Huix-Rotllant, M. Progress in time-dependent density-functional theory. *Annu. Rev. Phys. Chem.* **63**, 287–323 (2012).

13. Lee, S., Shostak, S., Filatov, M. & Choi, C. H. Conical intersections in organic molecules: benchmarking Mixed-Reference Spin–Flip Time-Dependent DFT (MRSF-TD-DFT) vs Spin–Flip TD-DFT. *J. Phys. Chem. A* **123**, 6455–6462 (2019).

14. S. Lee, S., Filatov, M., Lee, S. & Choi, C. H. Eliminating spin-contamination of spin-flip time dependent density functional theory within linear response formalism by the use of zeroth-order mixed-reference (MR) reduced density matrix. *J. Chem. Phys.* **149**, 104101, (2018).

15. Lee, S., Kim, E. E., Nakata, H., Lee, S. & Choi, C. H. Efficient implementations of analytic energy gradient for mixed-reference spin-flip time-dependent density functional theory (MRSF-TDDFT). *J. Chem. Phys.* **150**, 184111, (2019).

16. McWeeny, R. *Methods of molecular quantum mechanics*, (Academic press, 1992).

17. Tamm, I. Relativistic interaction of elementary particles. *J. Phys.(USSR)* **9**, 449 (1945).

18. Dancoff, S. Non-adiabatic meson theory of nuclear forces. *Phys. Rev* **78**, 382 (1950).

19. Lu, T. & Chen, F. Multiwfn: a multifunctional wavefunction analyzer. *J. Comput. Chem.* **33**, 580–592 (2012).

20. E. Espinosa, E., Molins, E. & Lecomte, C. Hydrogen bond strengths revealed by topological analyses of experimentally observed electron densities. *Chem. Phys. Lett* **285**, 170–173 (1998).

21. Taniguchi, M. & Lindsey, J. S. Database of absorption and fluorescence spectra of >300 common compounds for use in PhotochemCAD. *Photochem. Photobiol.* **94**, 290–327 (2018).

22. Beppu, T., Tomiguchi, K., Masuhara, A., Pu, Y. J. & Katagiri, H. Single benzene green fluorophore: solid-state emissive, water soluble, and solvent- and pH-independent fluorescence with large Stokes shifts. *Angew. Chem. Int. Ed.* **54**, 7332–7335 (2015).

23. Tang, B., Wang, C., Wang, Y. & Zhang, H. Efficient red-emissive organic crystals with amplified spontaneous emissions based on a single benzene framework. *Angew. Chem. Int. Ed.* **56**, 12543–12547 (2017).
